# A Low‐Symmetry Fe^II^ Tetrahedron From a Flexible Tritopic Ligand

**DOI:** 10.1002/chem.71043

**Published:** 2026-04-22

**Authors:** Rosemary J. Goodwin, Nina R. Lawson, Michael G. Gardiner, Jack D. Evans, Paul E. Kruger, Dan Preston

**Affiliations:** ^1^ Research School of Chemistry Australian National University Canberra ACT Australia; ^2^ Department of Chemistry School of Physics Chemistry and Earth Sciences University of Adelaide Adelaide SA Australia; ^3^ MacDiarmid Institute For Advanced Materials and Nanotechnology School of Physical and Chemical Sciences University of Canterbury Christchurch New Zealand

**Keywords:** iron(II), low symmetry, metallosupramolecular, solvent dependent, tetrahedron

## Abstract

Most high yielding low symmetry cages formed by metal–ligand coordination are assembled through strictly defined ligand and/or metal ion geometries which have been manipulated to form systems with complex architectures. In this work, we report the near‐quantitative synthesis of a new *S_4_*‐symmetry *meso* achiral [Fe_4_L_4_]^8+^ homoleptic face‐capped tetrahedron from a symmetric and flexible tris‐bidentate tritopic ligand. The symmetry of the tetrahedron is solvent mediated, with the *meso* cage forming near‐quantitatively in acetonitrile, while nitromethane causes the partial isomerisation to the racemic homochiral tetrahedron. The formation of this low symmetry tetrahedron is driven by ligand flexibility, which we attribute to the *meso* isomer effectively minimising its solvent shell and the ligand strongly favouring negative stereochemical coupling between metal centres, compared to the homochiral cage, thus being the dominant cage when in acetonitrile.

## Graphical abstract

A highly symmetric and flexible tris‐bidentate tritopic ligand forms an *S_4_ meso* homoleptic face‐capped Fe^II^ tetrahedron near‐quantitatively in acetonitrile. The addition of nitromethane solvent causes partial isomerization of this meso cage to the racemic homochiral tetrahedron, with the complexity of the system being moderated through solvophobic effects, aided by the flexibility of the symmetrical ligand.

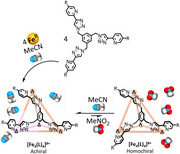

## 1 Introduction

The self‐assembly of rigid components has allowed the establishment of a simple but powerful set of rules that have provided predictable outcomes. This has given ready access to an impressive array of self‐assembled metallo‐supramolecular architectures from relativity simple components, with some remarkable examples emulating the size and function of biological assemblies. [1, 2, 3, 4, 5] These structures have the potential to serve as hosts for pollutants [6], reactive [7, 8] and therapeutic species [9], and catalytic processes [10, 11, 12], and in general show the capacity to act as analogues for naturally occurring molecules. A variety of approaches have been taken to vary the conformation and/or symmetry of these architectures [13, 14, 15, 16, 17]. Whilst significant advances have been made [18, 19, 20, 21, 22, 23, 24, 25, 26, 27, 28, 29], the potential to form complex, low symmetry structures remains a significant challenge.

An important class of metallo‐supramolecular cages are those formed from six‐coordinate octahedral metal ions, and with a subset of these architectures being tetrahedra, where the cage is either an edge‐capped [M_4_(L)_6_]^n+^ or face‐capped [M_4_(L)_4_]^n+^ structure depending on whether their ligands are *bis*‐bidentate ditopic [30] or *tris*‐bidentate tritopic [31, 32]. Upon formation of these tetrahedra, there is the possibility to obtain a mixture of the homochiral system (where each metal stereocentre is either *fac‐*ΔΔΔΔ or *fac‐*ΛΛΛΛ, forming an overall *T‐*symmetry), or the lower symmetry diastereomers, *C_3_* (*fac‐*ΔΔΔΛ or *fac‐*ΛΛΛΔ) or the *meso* system *S_4_* (*fac‐*ΔΔΛΛ), as well as low symmetry structures with both *mer* and *fac* metal centers, as reported by Ward [33] and Hooley [34, 35, 36]. Specifically for tetrahedron where all metal centers are *fac*, the expected statistical distribution of each isomer is 12.5%:50%:37.5% (*T*:*C_3_*:*S_4_*) [37] resulting in systems that either have strong or weak positive stereochemical coupling (i.e. favoring homochiral isomers, generally facilitated by rigid and/or shorter ligands, as highlighted by work from Lindoy [38, 39, 40], Ward [41, 42, 43, 44], and Raymond [45, 46]), or strong or weak negative stereochemical coupling (i.e. favoring achiral isomers, generally facilitated by flexible, longer ligands, as demonstrated by work reported by Saalfrank [47, 48, 49]) [50]. Deviations from this ratio may occur though depending on the relative stability of the isomers [51, 52, 53] with various reported systems showing interconversion between either the diastereomers or enantiomers/meso structures at room temperature [54] or by heating [47, 55], as well as reported examples inducing this rearrangement by solvent [56], guest templation [30, 57, 58, 59] or even pH [47, 49], highlighting the nuances of these systems. Despite the ability to bias these systems though ligand design and environmental stimuli, throughout the literature the homochiral *T* isomer still predominates [60, 61, 62, 63], with the quantitative formation of the lower symmetry architectures still being a challenge, as highlighted by Clegg [51, 52] and similarly, Nitschke and co‐workers [64] who found that by systematically increasing steric bulk within their family of [Fe_4_(L)_6_]^8+^ tetrahedral cages, they could favor the *T* isomer, while with less sterically demanding ligands they formed *S_4_* and *C_3_* isomers along with the *T* isomer.

The influence of environmental stimuli in selectively isolating specific isomers has been less widely investigated, despite solvophobic associations and collapse being a well‐known phenomenon in Nature, with a prime example being that of proteins and peptides, where the tertiary structure generally compresses to sequester hydrophobic elements away from polar solvent and is critical in folding processes and peptidic structure [65, 66]. This arises from both enthalpic and entropic contributions: polar solvents generally form stronger enthalpic interactions in the bulk rather than with the solute, while in the clathrate surrounding the solute has less freedom of movement. Both considerations lead to a minimization of the interface between solvent and solute and is energetically favorable. Solvophobic effects are also contributors to the folding of synthetic foldamers [24, 67, 68, 69, 70, 71], but have not been as extensively explored with regards to flexible ligands in coordination cages [1, 72, 73, 74, 75]. Toste and coworkers demonstrated the potential of solvophobic collapse with their parent face‐capped [Ga_4_(L)_4_]^12–^ complex, which due to the flexibility of the *tris*‐bidentate tritopic ligand initially adopts *S_4_* symmetry in D_2_O/CD_3_OD but upon increasing DMSO content, they found that the cage would rearrange and form the homochiral *T* diastereomer [56].

**FIGURE 1:**
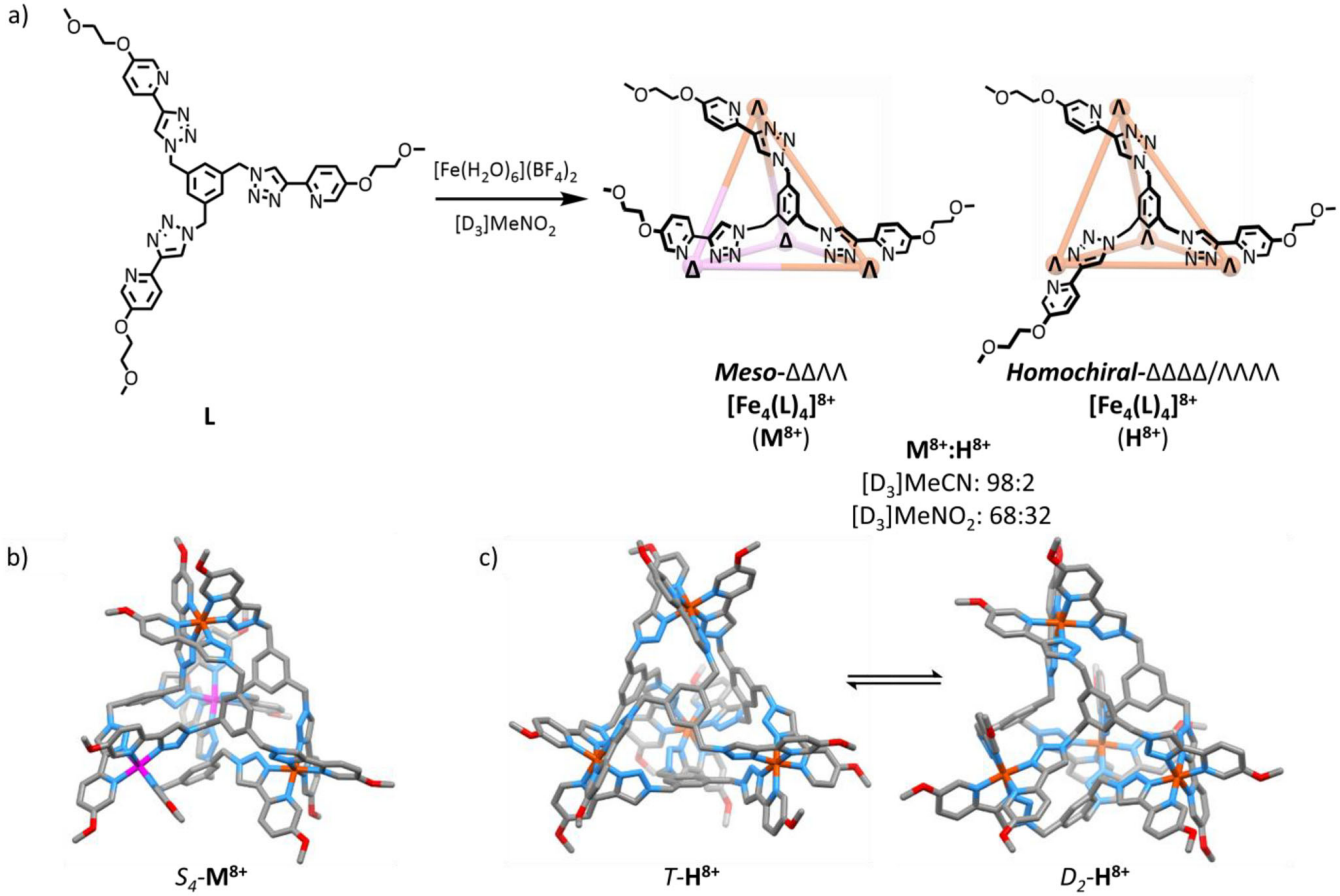
(a) Self‐assembly of solvent dependent face‐capped tetrahedra [Fe_4_(L)_4_]^8+^ (meso‐**M^8+^** and homochiral‐**H^8+^**) from tritopic tris‐bidentate ligand L. Normalized ratios of M:H in [D_3_]MeCN and [D_3_]MeNO_2_ determined from ^1^H NMR experiments; (b‐c) r^2^SCAN‐3c/CPCM(MeCN)‐optimized structures of cages reported in this work; b) known structure of S_4_‐**M^8+^**; (c) proposed structures of homochiral cages in equilibrium: T‐**H^8+^** (middle) and D_2_‐**H^8+^** (right). Colors: carbon: grey, nitrogen: blue, oxygen: red, Λ−Fe^II^: orange, Δ−Fe^II^: pink. Hydrogen atoms omitted for clarity.

In the current work, we were interested in how the use of flexible *tris‐*bidentate ligands might influence the structural identity and conformation of the resulting architectures when allowed to self‐assemble with Fe^II^. We report here a *C_3_* symmetric tritopic ligand (**L**, Figure 1a), that couples both high symmetry *and* flexibility provided by three methylene groups connecting the phenyl core to the 2‐pyridyl‐1,2,3‐triazole bidentate chelators. Upon combination with Fe^II^, a *meso* face‐capped tetrahedron with *S_4_* symmetry forms near‐quantitatively (Figure 1b). Interestingly, the complexity of the system is solvent moderated, with the formation of both the *meso S_4_* (**M**) isomer as the major species and an apparent (racemic) *homochiral T* (**H**) species being realized through introduction of nitromethane as solvent. We speculate that this apparent *T* isomer is predominantly collapsed into a conformation with *D_2_* symmetry (Figure 1c).

## 2 Results and Discussion

### 2.1 Synthesis of [Fe_4_(L)_4_](BF_4_)_2_

**FIGURE 2:**
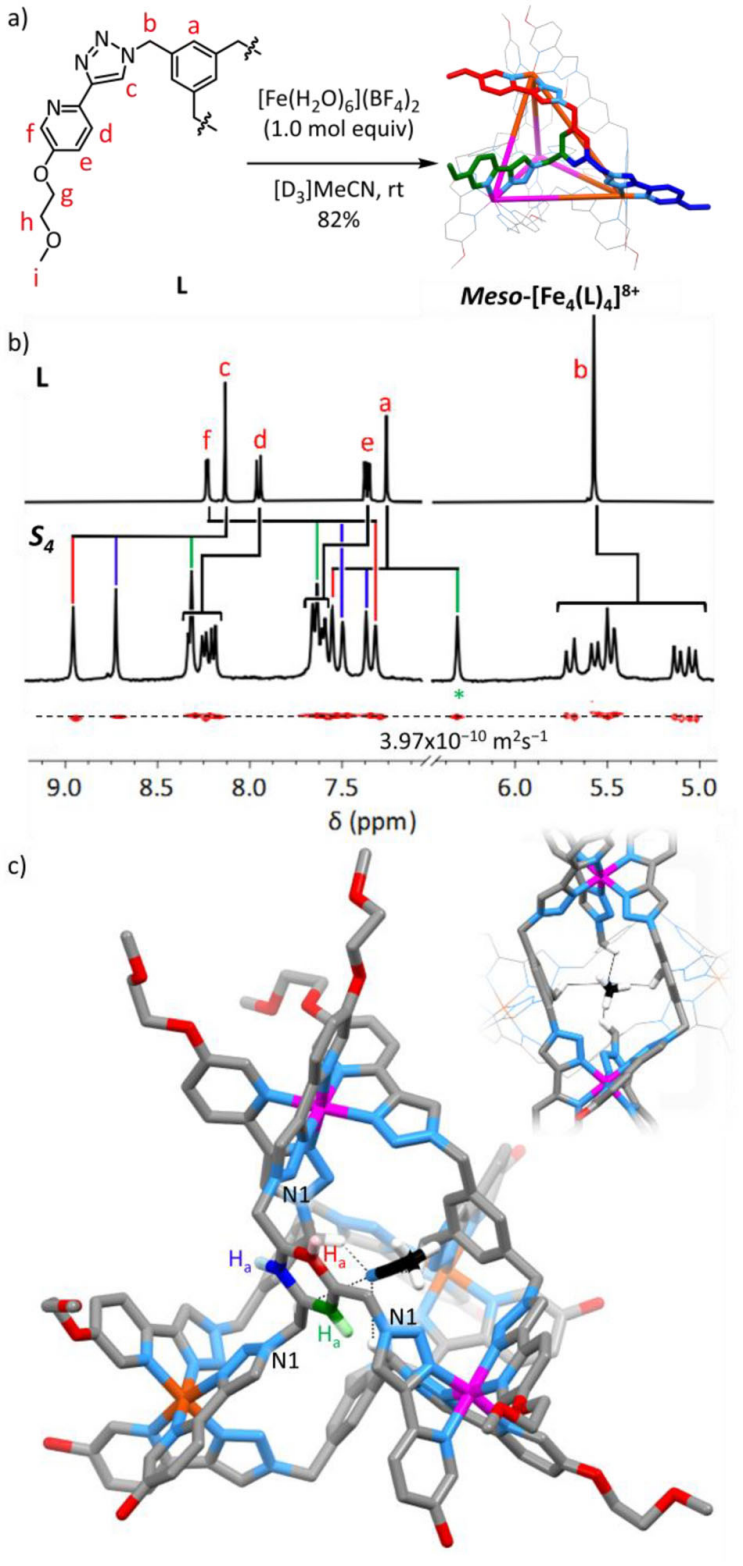
(a) Synthesis of ***M*‐[Fe_4_(L)_4_](BF_4_)_8_** (lettering of **L** and coloring of ***M*‐[Fe_4_(L)_4_](BF_4_)_8_** (Arm 1, Arm 2, Arm 3) corresponds to labelling in partial ^1^H NMR spectra in Figure 2b; theoretical structure of ***M*‐[Fe_4_(L)_4_]^8+^** used for clarity); (b) partial ^1^H NMR and ^1^H DOSY NMR spectra showing addition of [Fe(H_2_O)_6_](BF_4_)_2_ to form ***M*‐[Fe_4_(L)_4_](BF_4_)_8_** with colors corresponding to desymmetrized protons on ***M*‐[Fe_4_(L)_4_](BF_4_)_8_** (Arm 1, Arm 2, Arm 3; (*) indicates highly shielded methylene C─H_a_ on Arm 3; 400 MHz, [D_3_]acetonitrile, 298 K); (c) single X‐ray crystal structure ***M*‐[Fe_4_(L)_4_](BF_4_)_8_** with insert showing encapsulation of solvent guest. Dotted lines indicate a close contact shorter that the van der Waals’ radii of H and N [76]. Colors: methylene C─H_a_ on Arm 1: burgundy/light red, methylene C─H_a_ on Arm 2: royal blue/light blue, methylene C─H_a_ on Arm 3: green/light green, cage carbon: grey, solvent carbon: black, nitrogen: blue, oxygen: red, Λ−Fe^II^: orange, Δ−Fe^II^: pink. Non‐coordinating anions, cations and most hydrogens and solvent molecules are omitted for clarity, solvent mask feature was used (implemented within the Olex2 package [77]).

The ligand **L** (Figure 1) was made through three simple steps from 4‐hydroxy‐2‐bromopyridine: a Williamson ether synthesis, a Sonogashira coupling, and then a CuAAC “click” reaction, and obtained in 82% overall yield (see Supporting Information for further information, Section 1.3). The ligand was combined in a 1:1 ratio with [Fe(H_2_O)_6_](BF_4_)_2_ in [D_3_]acetonitrile, with equilibration occurring immediately. The resulting spectrum showed three sets of peaks per ligand environment, of equal intensity diffusing together as indicated by ^1^H DOSY NMR (Figure 2b). Mass spectral analysis confirmed the presence of a [Fe_4_(**L**)_4_]^8+^ structure or structures, that is, a face‐capped tetrahedron, with peaks corresponding to [Fe_4_(**L**)_4_ + nBF_4–_]^(8 – n)+^ for *n* = 3–5, for example for *n* = 4, *m*/*z* = 917.5266 (see Supporting Information for further information, Section 1.4).

2D NOESY and TOCSY NMR spectroscopy showed correlation between all three environments, with all three proton environments from the trimesityl core being within the same spin system suggesting formation of a single structure (Figure 2a). Hence, the splitting into three environments was consistent with each of the three arms of the ligand being in a different environment, with each of the four ligands in the tetrahedron being identically desymmetrized in terms of NMR analysis, indicating that the *C_3_* symmetry of **L** is broken during complexation to Fe^II^ (see Supporting Information for further analysis, Section 4). Lastly, it appeared from the upfield shift of one of the core protons (resonance H_a_ from Arm 3 (green *) at 6.30 ppm, Figure 2b) that one arm out of the three was in a significantly different environment than the other two. In contrast to previously reported metallo‐supramolecular cages with flexible linkers, heating the solution to 350 K showed no significant broadening or coalescence of these peaks, highlighting the thermodynamic stability of the obtained isomer, with no evidence of chirality flipping at individual metal centers (see Supporting Information for further analysis, Sections 1.4 and 2.6).

We were able to obtain single crystals of the compound in which connectivity could be observed, though the solubilizing chains were unable to be modelled due to disorder (*P*2_1_/*n*, R_1_ = 12.66, Figure 2c). Despite this, the core cationic structure was clear, with the structure being *meso* at the metal centers with *S_4_* symmetry. Consistent with the ^1^H NMR analysis, the methylene groups of the first two arms (C─H_a_ (red) and C─H_a_ (blue) Figure 2c) were orientated with their hydrogen atoms towards the exterior of the molecule, while the methylene group of the third arm was rotated so that the hydrogen atoms were orientated towards the center of the molecule (C−H_a_ (green)), in close proximity to those of the ‘other’ third arms from the other ligands. It is clear therefore that the *meso S_4_*−[Fe_4_(**L**)_4_](BF_4_)_2_ (**M·(BF_4_)_8_**) solid‐state structure persists in solution on the ^1^H NMR time scale.

Interestingly, one of the two edges between two homochiral metal ions was open in the X‐ray structure, creating a single niche‐like cavity into which an acetonitrile solvent molecule had inserted, hydrogen bonding in an octafurcated fashion to the eight methylene protons at the terminus of the cavity (C─H_a_ (green), C─H···N distances ranged from 2.48 to 3.07 Å, with an average of 2.81 Å, Figure 2c) [76]. While methylene protons are generally not particularly acidic, these are all adjacent to N1 within the 1,2,3‐triazole, and binding affinities combine multiplicatively with increasing number of interactions. Whilst one side of **M·(BF_4_)_8_** maintains this enlarged cavity with a solvent guest, the other homochiral edge on the opposite side of the tetrahedron appears to collapse with no potential for guest encapsulation (see Supporting Information for further information, Section 3). Whilst not all anions could be resolved, the BF_4_^−^ anions that were observed within the solid state structure appear to have no significant interaction with the cage cavity, rather existing on the periphery of the complex, consistent with analysis of the ^19^F NMR spectrum, which only shows free BF_4_^−^. Though there are several literature examples where charge dense anions bind strongly to the cage architecture [30], we see no such evidence in this system despite testing a range of different guests, potentially due to the compressed nature of **M·(BF_4_)_8_**, as observed within similarly compressed systems [56] (see later discussion and Supporting Information for further information, Section 2).

The presence of only three environments in the ^1^H NMR spectrum indicates either that solution‐phase ingress of acetonitrile in this cavity is under fast exchange conditions, or that, in solution, acetonitrile simultaneously enters both edges. Low temperature VT NMR studies in [D_3_]acetonitrile to 233 K (or below in mixed solvent systems, see below) and 2D DOSY NMR showed no evidence either way. To further interrogate whether solvent persists within the pocket computational interaction site screening (aISS//GFN1‐FF [78]) calculations were undertaken using the intermolecular force‐field xTB‐IFF [79], with the full method implemented in xTB 6.7.1. The obtained solvent included systems were optimized using the semi‐empirical GFN1‐xTB [80] method, with these structures indicating that only one solvent molecule can favorably enter the structure, while any extra solvent molecules exist outside of the cage cavity (see Supporting Information for further information, Section 5.3). This is consistent with the crystal structure (see Supporting Information for structure comparisons, Section 5.4.2) and appears to be due to the cage deforming and enlarging one side of the tetrahedron to encapsulate a solvent molecule (see below for cavity volume analysis). Density functional theory (DFT) calculations (r^2^SCAN‐3c/CPCM(MeCN) [81, 82], with one explicit acetonitrile encapsulated within the cage, indicate that these interactions between the tetrahedron cavity and a bound solvent are favorable, and as such, we suggest that, within solution, the cage does exist with one solvent molecule under fast exchange conditions.

### 2.2 Solvent Mediated Perturbations of the Symmetry of [Fe_4_(L)_4_](BF_4_)_2_

**FIGURE 3:**
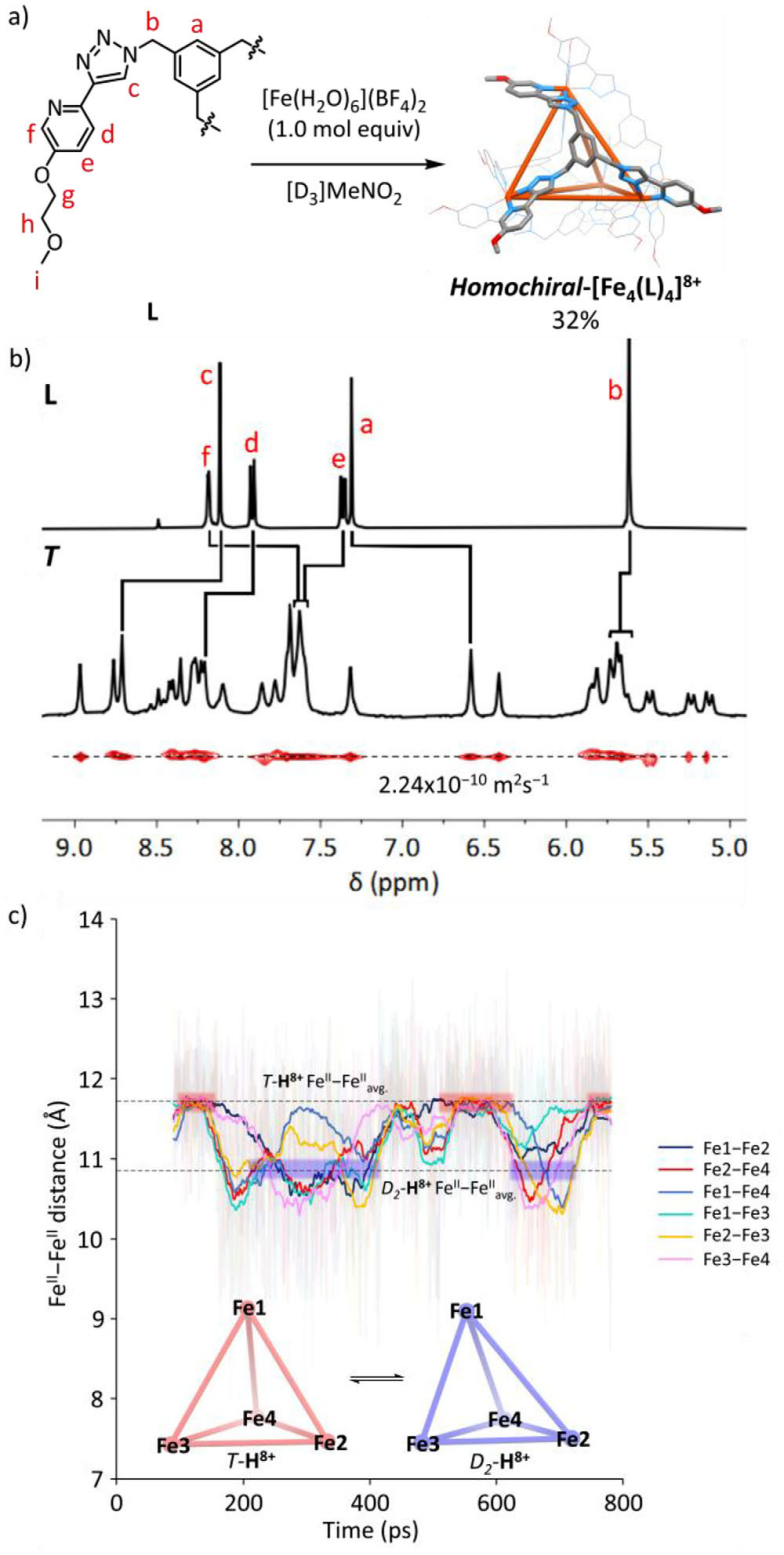
(a) Self‐assembly of ***H*‐[Fe_4_(L)_4_](BF_4_)_8_** (lettering of **L** corresponds to labelling in partial ^1^H NMR spectra in Figure 3b; theoretical structure of *D_2_*‐**[Fe_4_(L)_4_]^8+^** used for clarity); (b) partial ^1^H NMR and ^1^H DOSY NMR spectra showing addition of [Fe(H_2_O)_6_](BF_4_)_2_ to form both ***M*‐[Fe_4_(L)_4_](BF_4_)_8_** and ***H*‐[Fe_4_(L)_4_](BF_4_)_8_** in 68:32 ratio. Only proton resonances corresponding to ***H*‐[Fe_4_(L)_4_](BF_4_)_8_** are identified, all other non‐labelled resonances correspond to ***M*‐[Fe_4_(L)_4_](BF_4_)_8_** (400 MHz, [D_3_]nitromethane, 298 K). Colors: cage carbon: grey, nitrogen: blue, oxygen: red, Fe^II^: orange; (c) MD simulation time trace (1000 K) of **H^8+^** showing changes in intramolecular Fe^II^−Fe^II^ distance (Å). Pale lines show full data, bold trend lines show rolling average for each Fe^II^−Fe^II^ distance (intervals of 50 ps). Dotted lines at 10.8 Å and 11.7 Å represent the average Fe^II^−Fe^II^ distance of *D_2_‐* **H^8+^** and *T‐* **H^8+^** calculated from initial starting geometries. Transparent red and blue boxes along these dotted lines indicate when **H^8+^** is predominantly a *T‐* **H^8+^** or *D_2_‐* **H^8+^** isomer, respectively.

We hypothesized that the interaction between the acetonitrile guest and **M·(BF_4_)_8_** helped drive the formation of the *meso* architecture. Acetonitrile is a ‘thin’ linear molecule, and we were interested to see the influence of solvents with a similar profile, such as nitromethane, on the self‐assembly process. A broader comparison of the solvents shows they both have similar dielectric constants (MeCN = 36.0, MeNO_2_ = 35.9 [83]), while nitriles are better hydrogen bond acceptors (β = 4.7) than nitro groups (β = 3.7) [84]. Accordingly, we combined a 1:1 ratio of [Fe(H_2_O)_6_](BF_4_)_2_ and **L** in [D_3_]nitromethane. The system equilibrated after heating at 328 K for 30 min (Figure 3a), with a new species being present within the ^1^H NMR, along with the previously identified **M·(BF_4_)_8_** isomer, with all species diffusing at the same rate by DOSY NMR (Figure 3b).

Mass spectrometry of the sample was effectively identical to **M·(BF_4_)_8_**, indicating that all species present were [Fe_4_(**L**)_4_]^8+^ face‐capped tetrahedra. However, 2D NOESY and TOCSY spectra indicated an assembly with higher symmetry (since there was only one proton environment per arm on the NMR time scale, Figure 3b), and therefore by necessity being a (racemic mixture) of homochiral cages (**H·(BF_4_)_8_**) with apparent *T* symmetry. The normalized ratio between **H·(BF_4_)_8_** and **M·(BF_4_)_8_** was 0.32:0.68. Revisiting the spectrum of **M·(BF_4_)_8_** in acetonitrile, we were now able to detect trace amounts of **H·(BF_4_)_8_** (2:98 **H·(BF_4_)_8_**:**M·(BF_4_)_8_**). Furthermore, by varying the ratio of [D_3_]acetonitrile to [D_3_]nitromethane, we found that as the proportion of nitromethane to total solvent decreased, the ratio of **M·(BF_4_)_8_** to **H·(BF_4_)_8_** increased in a roughly linear relationship (see Supporting Information for further information, Section 1.4.2). The relative ratios of the *meso* and homochiral cages allows calculation of their energies in the two solvents. Assigning **M^8+^** as the ‘product’, in acetonitrile, *K*_298K_ = 50 and Δ*G*_298K_ = −9.6 kJ mol^−1^, while in nitromethane, *K*_298K_ = 2.1 and Δ*G*_298K_ = −1.9 kJ mol^−1^.

We hypothesized that a solvent of comparable length to nitromethane but with more bulk might favor **H·(BF_4_)_8_** over **M·(BF_4_)_8_** and investigated [D_6_]acetone. We repeated the formation of [Fe_4_(**L**)_4_]^8+^ in varying ratios of [D_3_]acetonitrile/[D_6_]acetone and were able to observe the *meso* isomer in the spectra obtained, but at higher proportions of acetone there were also multiple broad peaks and spectral untidiness (see Supporting Information for further information, Section 1.4.3). It is unclear if this is due to solvent size or the lower coordinative ability of acetone meaning that the system could not thermodynamically equilibrate to a single major isomer. The introduction of highly polar solvents ([D_6_]DMSO, D_2_O) into [D_3_]acetonitrile solutions of the cage led to stripping of the metal ions from the structure by the coordinative solvent, that is, spectra showing ‘free’ ligand or broad peaks consistent with fluxionality and/or high spin Fe^II^, in keeping with previous results from Fe^II^ and 2‐pyridyl‐1,2,3‐triazole ligands [29, 85]. Interestingly, in [D_4_]methanol we initially observed no formation of any supramolecular architectures, primarily due to solubility issues. Only with an increasing percentage of [D_3_]acetonitrile did **M·(BF_4_)_8_** start to form, along with minor components of **H·(BF_4_)_8_** (∼2:98 **H·(BF_4_)_8_**:**M·(BF_4_)_8_**), indicating again that the self‐assembly of **M·(BF_4_)_8_** and the strong negative stereochemical communication between the Fe^II^ vertices is primarily driven by the ligand forming favorable solvent interactions [56].

To determine the structure of the proposed homochiral *T* [Fe_4_(**L**)_4_]^8+^ isomer, we again employed DFT calculations (r^2^SCAN‐3c/CPCM(MeCN), Figure 1b), and found that the *T* isomer was lower in calculated energy to the *S_4_* cage with an implicit acetonitrile solvent field, by 49 kJ mol^−1^. Even after considering stabilization through binding a solvent molecule within **M^8+^⊂NCMe**, it was clear that calculations were not accurately capturing the energetics of the system, given the energetic preference of the system for the *S_4_* cage. This turned out to be a common finding across most computational comparisons made in this study. Considering the flexibility of the ligand and the collapsed structure of **M^8+^**, we postulated that this was due to computational underestimation of the solvophobic effect. Whilst significant steps have been made in modelling solvent effects [86, 87, 88], due to the size of the systems reported we were limited to implicit solvent fields. These solvent fields can effectively capture the enthalpic contributions of solvent interacting with the cage, however they do not account for other energetic contributions of solvent [89, 90, 91, 92]. Namely, they cannot account for the higher enthalpic favorability of solvent being in the bulk rather than at the interface [93, 94, 95], or the entropic considerations due to loss of translational and rotational freedom of solvent molecules in clathrates [96, 97, 98]. Both of these can significantly contribute to conformational arrangements which minimize the interface between polar solvent and hydrophobic areas of the solute [99, 100, 101, 102]. With rigid ligands we hypothesize that this is less important as solvophobic collapse is not possible. When comparing the *S_4_* and *T* computational structures (using the *S_4_* **M^8+^⊂NCMe** computational structure derived from computations and matching the X‐ray structure), we found that the *T* structure had greater total surface area (i.e. Connolly surface, calculated by MoloVol v1.1.1 [103], 1992 Å^2^ compared to 1937 Å^2^ for a difference of 55 Å^2^), and greater total cavity volume (196 Å^3^ compared to 85  Å^3^, for a difference of 111 Å^3^, see Supporting Information for further information, Section 5.5.2). Similar results were found when using the *S_4_* structure with nitromethane bound. We therefore attribute the favorability of the *meso* cage over a presumptive *T* structure in part to the entropic contributions to solvent effects, which are not accounted for in these calculated energies.

Given the compressed structure of the **M·(BF_4_)_8_** isomer which we now attributed at least in part to the collapse of the internal cage cavity, we began to question whether **H·(BF_4_)_8_** truly had a predominantly open cage cavity with *T* symmetry. We hypothesized instead that it might be similar to the **M·(BF_4_)_8_** geometry with two methylene environments of each ligand ‘out’ and one ‘in’, and hence have similar small channels to **M·(BF_4_)_8_**, but instead be homochiral. This proposed structure would have *D_2_* symmetry, and we anticipated that it might be able to flip between different methylene environments being ‘out’ or ‘in’ on the ^1^H NMR timescale due to its homochiral character. This would give a time averaged NMR spectrum that appeared higher symmetry than the true complex, with the flexibility and dynamic nature of the ligands allowing conformational interchange between the more open *T* symmetric cage and a compressed cage with approximate *D_2_* symmetry [104].

To investigate the dynamic nature of **H·(BF_4_)_8_**, we again turned to VT NMR spectroscopy in a mixed solvent system (1:3 [D_3_]acetonitrile:[D_3_]nitromethane). As stated earlier, for **M·(BF_4_)_8_**, the greatest variance of chemical shifts for a desymmetrized environment was for the H_a_ proton. Assuming the same trend to be likely true for **H·(BF_4_)_8_**, with decreasing temperature one would expect coalescence to first occur for the H_a_ proton of the homochiral cage. Lowering the temperature indeed saw a broadening of this peak with coalescence observed at 223 K (see Supporting Information for further information, Figure S1.29). Further cooling to 193 K did not reveal decoalescence into three peaks, but the disappearance of the H_a_ resonances suggests underlying structural complexity that is masked at higher temperatures by fast exchange on the NMR time scale. Again using both DFT calculations (r^2^SCAN‐3c/CPCM(MeCN)) and molecular dynamic (MD) simulations (GFN1‐xTB [80], implemented within xTB 6.7.1 in implicit acetonitrile at 1000 K), we were able to identify a speculative homochiral conformation with *D_2_* symmetry (Figure 1b), with MD models demonstrating that over time, the initially open and enlarged *T‐* **H^8+^** cage can collapse to a *D_2_‐* **H^8+^** conformation (and *vice versa*, Figure 3c), causing the Fe^II^ metal ions to come closer in proximity. While this compressed homochiral cage was again higher in energy than the *T* conformer (by 88 kJmol^−1^), as with the comparison to the *S_4_* cage this does not include some solvent effects. Comparing the solvent accessible surface of *D_2_‐* **H^8+^** and *T‐* **H^8+^**, the *D_2_‐* **H^8+^** structure has a smaller solvent shell (for *D_2_* with nitromethane bound, 1962 Å^2^ compared to 1992 Å^2^ for a difference of 30 Å^2^) and a smaller total cavity area (117 Å^3^ compared to 196 Å^3^, for a difference of 79 Å^3^). It should also be noted that, similarly to *S_4_*‐**M^8+^**, *D_2_‐* **H^8+^** maintains a hydrophobic internal niche along the edge between the two Fe^II^ metal ions spanned by two arms of the ligand, that allows close encapsulation of a solvent guest, while *T‐* **H^8+^** cannot form a similar well‐defined cavity due to the more open structure of the cage. The combination of our analysis of computational derived solvent accessible surface areas/cavities, and the coalescence of the H_a_ resonance of **H·(BF_4_)_8_** in VT studies indicating fluxional behavior, leads us to propose that the homochiral cage exists in conformational interchange between states with *T* and *D_2_* symmetry, with the *D_2_* conformation energetically favored. In this, the three environments would interchange through a *T* conformational transition state, rendering a ^1^H NMR spectrum that is high‐symmetry on the NMR time scale, as also indicated by MD analysis.

We emphasize that this hypothesis carries a degree of speculation, based as it is on the coalescence of the H_a_ resonance at low temperature. Nonetheless, the similarities between the *S_4_* and *D_2_* structures are striking. Both have collapsed cavities. Both also have two clefts, each formed along edges between two metal centers of the same stereochemistry. The structures only differ in whether the metal ions at the edge with the cleft have the same chirality or not as the metal ions at the opposite cleft. We therefore speculate that the two metal ions at the edge containing the cleft exhibit high positive stereochemical coupling to one another, but the overall negative stereochemical coupling exhibited in the system in acetonitrile (i.e. the predominance of the *S_4_* isomer) is driven by solvophobic collapse. We note that the most statistically probable tetrahedral isomer, *C_3_*, is not observed in this system, and tentatively propose that it is because a *C_3_* structure cannot form two clefts opposite one another in this manner.

### 2.3 Solvent Shell Changes Between *D_2_‐* **H^8+^** and **M^8+^**

**FIGURE 4:**
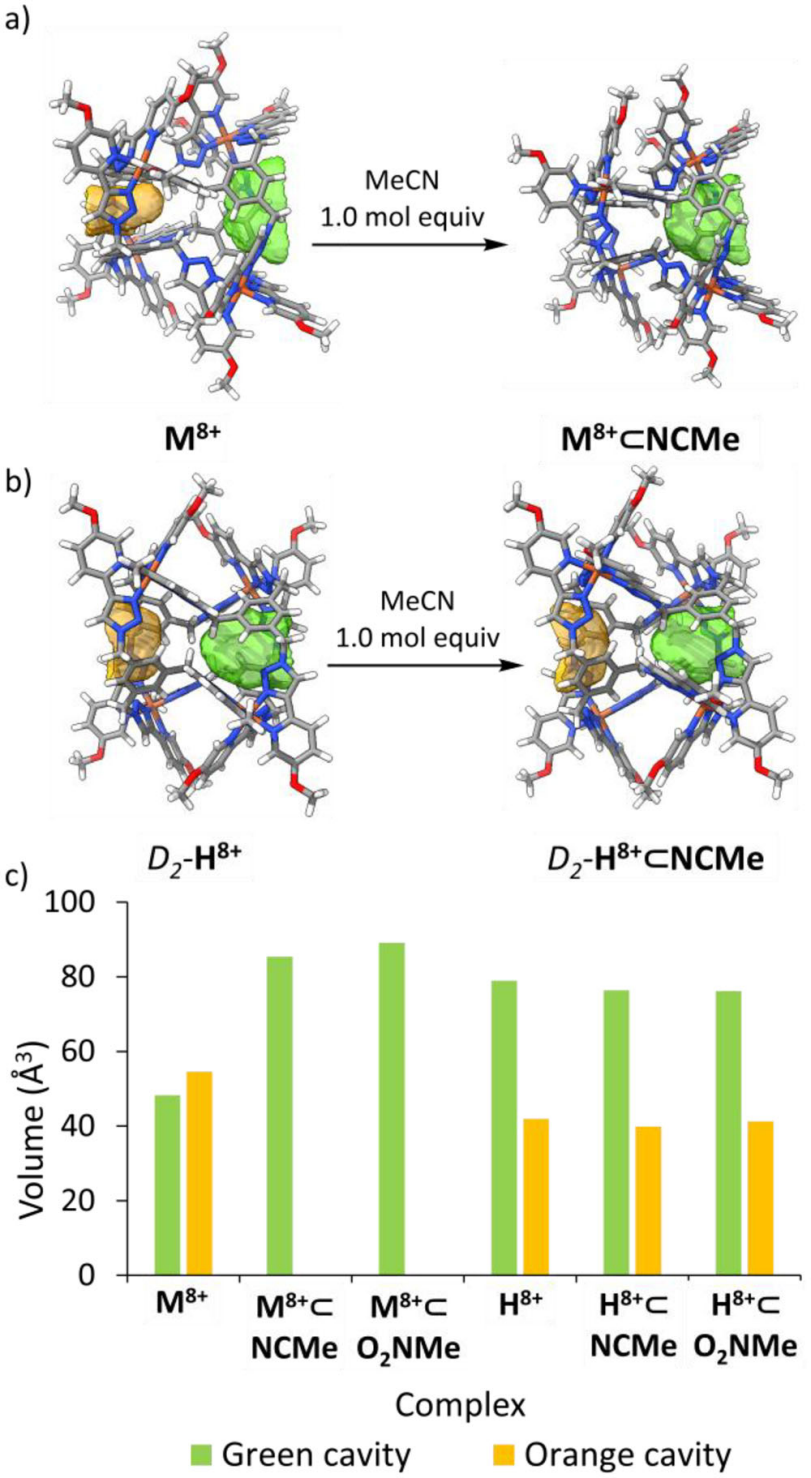
(a) r^2^SCAN‐3c/CPCM(MeCN)‐optimized structures of **M^8+^⊂NCMe** showing enlargement of green internal cavity and collapse of yellow cavity upon encapsulation of an explicit acetonitrile solvent guest; (b) r^2^SCAN‐3c/CPCM(MeCN)‐optimized structures of *D_2_‐* **H^8+^⊂NCMe** showing minimal structural change upon encapsulation of an explicit acetonitrile solvent guest; (c) internal cavity volumes of both **M^8+^**, **M^8+^⊂NCMe** and **M^8+^⊂O_2_NMe**, and *D_2_‐* **H^8+^**, *D_2_‐* **H^8+^⊂NCMe** and *D_2_‐* **H^8+^⊂O_2_NMe**.

Given our hypothesis about the importance of minimization with solvent accessible area and cage structure, we carried out a similar analysis comparing the *S_4_* and *D_2_* cages to investigate their relative favorability. We carried out DFT calculations (r^2^SCAN‐3c/CPCM(MeCN)), comparing the two isomers with either an explicit acetonitrile or nitromethane solvent molecule encapsulated within the cavity of *D_2_‐* **H^8+^** or **M^8+^**. Again, in terms of calculated energies these did not effectively represent the observed equilibrium position, although binding either solvent in the cleft of either cage was enthalpically favored. We therefore investigated their respective solvent shells and cavities, through the programme MoloVol v1.1.1, using the optimized structures **M^8+^** and *D_2_‐* **H^8+^**, **M^8+^⊂NCMe** and *D_2_‐* **H^8+^⊂NCMe**, **M^8+^⊂O_2_NMe** and *D_2_‐* **H^8+^⊂O_2_NMe**. Cavity volume analysis of calculated structures *D_2_‐* **H^8+^** and **M^8+^** indicated that without solvent inclusion, **M^8+^** maintains internal cavities that are effectively equal, whilst *D_2_‐* **H^8+^** has one cavity that is almost double that of the other cavity (Figure 4c). Upon incorporation of a solvent molecule, both **M^8+^⊂NCMe** and **M^8+^⊂O_2_NMe** show an enlargement of one cavity (thus accommodating the encapsulated solvent guest), whilst the secondary internal cavity collapses, disfavoring any inclusion of solvent within this cavity (Figure 4a). Interestingly, inclusion of solvent within *D_2_‐* **H^8+^** seems to have minimal effect on the overall structure and cavity size of the tetrahedron (Figure 4b). In other words, encapsulation of solvent in the *meso* structure brings about complete collapse of the second cavity, while this is not observed in the homochiral structure.

This results in total cavity volume for the *meso* isomer that is 28 – 31 Å^3^ smaller compared to the homochiral *D_2_* cage, depending on whether acetonitrile or nitromethane is bound. Similarly, analysis of the effective solvent accessible surface area (i.e. Connolly surface) indicates upon inclusion of a solvent molecule, both **M^8+^⊂NCMe** and **M^8+^⊂O_2_NMe** show a smaller solvent shell compared to *D_2_‐* **H^8+^⊂NCMe** and *D_2_‐* **H^8+^⊂O_2_NMe** respectively, with the Connolly surface of *D_2_‐* **H^8+^⊂solvent** being on average 26 Å^2^ larger than **M^8+^⊂solvent**. Our calculations suggest that the *meso* isomer is able to compress solvent accessible cavities and total surface area more effectively than the homochiral isomer, and thereby reduce interface between solvophobic regions of the cage and the solvent. We therefore attribute the differences in isomer populations between acetontrile and nitromethane to the relative differences in the hydrogen‐bonding capability. While both solvents are poor hydrogen bond donors, acetonitrile is a significantly better hydrogen bond acceptor (see above). Acetontirile solvent should therefore lead to higher enthalpic unfavourablility at the interface rather than the bulk, as well as entropic unfavourability due to formation of a more rigid hydrogen‐bonding framework in the solvent shell. Conversely, in nitromethane, this cost is reduced, and accordingly the predominance of the *meso* cage with the smaller surface area should decrease as observed.

While the above calculations are consistent with what is observed in solution, we acknowledge again the degree of speculation about the existence of the *D_2_* conformation. Statistically we expect a 3:1 ratio between *S_4_* and presumptive *T* isomers. This is close to what is observed in nitromethane, but not in acetonitrile. While the change in isomer distibtuion between the two solvents is not immense, the computational arguments as to its source seem reasonable to us.

### 2.4 Guest Binding Investigations

As already discussed, **M·(BF_4_)_8_** maintains a small cavity and appears to have conformational flexibility, and so we were interested to analyze whether this is a favorable cavity for guests other than solvents. We analyzed a variety of anionic guests (point charge and linear geometries), however saw no encapsulation within the cavity, with the guests generally associating on the exterior of the cage with the acidic triazole protons (see Supporting Information for further information, Section 2).

## 3 Conclusion

The controlled formation of low symmetry tetrahedra is still relatively nuanced, especially when derived from a single, flexible, high‐symmetry ligand. In this work, we have reported the near‐quantitative synthesis in acetonitrile of a *S_4_ meso* homoleptic face‐capped tetrahedron from a symmetric and flexible tris‐bidentate tritopic ligand. The addition of nitromethane solvent causes partial isomerization of this *meso* architecture to the racemic homochiral tetrahedron. The flexibility of the ligand allows collapse of the cages. For the *meso* cage, collapse results in reduced surface area and a single niche cavity in which solvent binds. In contrast, the proposed homochiral cage structure cannot undergo the same degree of collapse, and therefore maintains a slightly larger surface and a total cavity volume. The partial emergence of the homochiral isomer in nitromethane appears to be due in part to the reduced energetic costs of the larger solvent shell in this solvent, likely due to the lower hydrogen bonding acceptor capability of nitro groups compared to nitriles. We are now systematically modifying ligand cores to tune the character of these unique cavities formed from solvent induced collapse, opening new avenues for targeting host‐guest function in this class of materials.

## 4 Experimental Section

Synthetic procedures, characterisation data, metal binding studies, details of X‐ray crystallography and computational chemistry methods are provided in the Supporting Information.

## Conflicts of Interest

The authors declare no conflicts of interest.

## Supporting information



The authors have cited additional references within the Supporting Information [105–126].

## Data Availability

The data that support the findings of this study are available in the supplementary material of this article. Crystal data for *S_4_*−[Fe_4_(**L**)_4_](BF_4_)_2_ (CCDC: 2504227) has been deposited with the Cambridge Crystallographic Data Centre.
