# Supplementary material for: A Low‐Symmetry FeII Tetrahedron From a Flexible Tritopic Ligand
Source: Chemistry. 2026 Apr 22;32(26):e71043. doi: 10.1002/chem.71043 (PMC13356432; doi:10.1002/chem.71043)
Supplement: Supplementary file 1 — The authors have cited additional references within the Supporting Information [105–126]. [file CHEM-32-e71043-s001.pdf]

Supporting Information

A Low-symmetry Fe^{II} Tetrahedron from a Flexible Tritopic Ligand

Rosemary J. Goodwin,[a] Nina R. Lawson,[a] Michael G. Gardiner,[a] Jack D. Evans,[b] Paul E. Kruger,[c] and Dan Preston[a]

a] Dr. R. J. Goodwin, N. R. Lawson, Dr. M. G. Gardiner, [*] Dr. D. Preston
Research School of Chemistry, Australian National University
Canberra ACT 2601 (Australia)

b] Dr. J. D. Evans
Department of Chemistry, School of Physics, Chemistry and Earth Sciences, University of Adelaide, Adelaide SA 5000 (Australia)

c] Prof. P. E. Kruger
MacDiarmid Institute for Advanced Materials and Nanotechnology, School of Physical and Chemical Sciences
University of Canterbury
Christchurch 8140 (New Zealand)

***daniel.preston@anu.edu.au**

Contents

1. Experimental 5
   1.1. General 5
      1.1.1. General complexation details 5
      1.1.2. NMR pulse programs and parameters 5
   1.2. Precursors 6
      1.2.1. A 6
      1.2.2. B 7
   1.3. Ligand 8
      1.3.1. L 8
   1.4. Complexes 10
      1.4.1. $[Fe_4(L)_4](BF_4)_8$ (S_4 complex) in acetonitrile 10
      1.4.2. Complexations in nitromethane 16
      1.4.3. Complexations in $[D_6]$acetone 21
      1.4.4. Complexations in $[D_6]$DMSO 22
      1.4.5. Complexations in D_2O 23
      1.4.6. Complexations in CD_3OD 23
2. NMR studies of guest binding 24
   2.1. Procedure for ^{1}H NMR titration experiments 24
   2.2. Anion binding data, NMR spectra and isotherms 24
   2.3. N_3^- binding to $M\cdot(BF_4)_8$ 25
   2.4. NO_2^- binding to $M\cdot(BF_4)_8$ 25
   2.5. Cl^- binding to $M\cdot(BF_4)_8$ 26
   2.6. Br^- binding to $M\cdot(BF_4)_8$ 27
      2.6.1. Br^- binding to $M\cdot(BF_4)_8$ at 298 K 27
      2.6.2. Br^- binding to $M\cdot(BF_4)_8$ at 333 K 28
3. Crystallography 29
   3.1. Structure of $[S_4\text{-}[Fe_4(L)_4\subset NCMe]Na\cdot(BF_4)_6\cdot MeCN]^{3+}$ 29
4. Symmetry elements 33
5. Calculations 35
   5.1. General 35
      5.1.1. Initial structure optimisation 35
      5.1.2. Molecular dynamic simulations 35
      5.1.3. Solvent docking calculations 35

5.2. Dynamic equilibrium of D_2–H^{8+} and T–H^{8+} 36
5.3. Docking studies for $M^{8+}\subset solvent_x$ and D_2–$H^{8+}\subset solvent_x$ 37
5.3.1. Docking between M^{8+} and $MeCN_x$ 38
5.3.1.1. Docking between $M^{8+}\subset NCMe$ 38
5.3.1.2. Docking between $[M^{8+}\subset NCMe]MeCN$ 38
5.3.2. Docking between M^{8+} and $(MeNO_2)_x$ 39
5.3.2.1. Docking between $M^{8+}\subset O_2NMe$ 39
5.3.2.2. Docking between $[M^{8+}\subset O_2NMe]MeNO_2$ 39
5.3.3. Docking between D_2–H^{8+} and $MeCN_x$ 40
5.3.3.1. Docking between D_2–$H^{8+}\subset NCMe$ 40
5.3.3.2. Docking between D_2–$H^{8+}\subset NCMe_2$ 40
5.3.4. Docking between D_2–H^{8+} and $(MeNO_2)_x$ 41
5.3.4.1. Docking between D_2–$H^{8+}\subset O_2NMe$ 41
5.3.4.2. Docking geometries between $[D_2$–$H^{8+}\subset O_2NMe]MeNO_2$ 41
5.4. Tetrahedra energies and solvent interaction energy 41
5.4.1. General 41
5.4.2. Structural comparison between $M^{8+}\subset NCMe$ and crystal structure $[S_4$-$[Fe_4(L)_4\subset NCMe]Na\cdot(BF_4)_6\cdot MeCN]^{3+}$ 43
5.4.3. Interaction between $M^{8+}\subset NCMe$ 44
5.4.4. Interaction between $M^{8+}\subset O_2NMe$ 44
5.4.5. Interaction between D_2–$H^{8+}\subset NCMe$ 45
5.4.6. Interaction between D_2–$H^{8+}\subset O_2NMe$ 45
5.5. Cavity calculations 46
5.5.1. General 46
5.5.2. Cavity volume and surface area of tetrahedra 46
5.5.2.1. Tetrahedra interior cavity volume 46
5.5.2.1. Tetrahedra surface area 46
5.5.3. Interior cavity volume and probe excluded surface area of M^{8+} 48
5.5.4. Interior cavity volume and probe excluded surface area of $M^{8+}\subset NCMe^{ghost}$ 49
5.5.5. Interior cavity volume and probe excluded surface area of $M^{8+}\subset O_2NMe^{ghost}$ 50
.... 50
5.5.6. Probe excluded surface area of $M^{8+}\subset NCMe$ 51
5.5.7. Probe excluded surface area of $M^{8+}\subset O_2NMe$ 51
5.5.8. Interior cavity volume and probe excluded surface area of D_2–H^{8+} 52

5.5.9. Interior cavity volume and probe excluded surface area of D_2–H^{8+}⊂NCMe^{ghost} 53
.... 53
5.5.10. Interior cavity volume and probe excluded surface area of D_2–H^{8+}⊂O_2NMe^{ghost}........ 54
.... 54
5.5.11. Interior cavity volume and probe excluded surface area of D_2–H^{8+}⊂NCMe 55
.... 55
5.5.12. Interior cavity volume and probe excluded surface area of *D2*–H^{8+}⊂O_2NMe............. 55
5.5.13. Interior cavity volume and probe excluded surface area of *T*–H^{8+} 56
6. References 57

1. Experimental

1.1. General

Unless otherwise stated, all reagents were purchased from commercial sources and used without further purification, except for:

2-Methoxyethyl p-toluenesulfonate[105]

which were synthesised according to literature procedures. Solvents were laboratory reagent grade. Petroleum ether refers to the fraction of petrol boiling in the range 40 – 60 °C. Abbreviations: dichloromethane (DCM), ethylenediaminetetraacetate (EDTA), tetrahydrofuran (THF), triethylamine (TEA), dimethyl sulfoxide (DMSO), dimethylformamide (DMF). ^{1}H and ^{13}C NMR spectra were recorded on either a Bruker Avance 400 MHz, 600 MHz or 700 MHz spectrometer. Chemical shifts are reported in parts per million and referenced to *residual solvent peaks*, labelled in spectral figures for the fully deuterated solvent used ($CDCl_3$: ^{1}H δ 7.26 ppm, ^{13}C δ 77.16 ppm; [D_3]acetonitrile: ^{1}H δ 1.94 ppm; [D_3]nitromethane: ^{1}H δ 4.30 ppm).[106] Coupling constants (*J*) are reported in Hertz (Hz). Standard abbreviations indicating multiplicity were used as follows: m = multiplet, q = quartet, quin = quintet, t = triplet, dt = double triplet, d = doublet, dd = double doublet, s = singlet, br = broad. Electrospray mass spectra (HR ESI-MS) were collected on a Waters Synapt G2-S1 HDMS spectrometer or an Orbitrap Elite spectrometer and Nanospray mass spectra (Nanospray MS) were collected on an Orbitrap Elite spectrometer.

CAUTION: WHILE NO PROBLEMS WERE ENCOUNTERED DURING THIS WORK, AZIDES ARE EXPLOSIVE AND CARE SHOULD BE TAKEN WHEN DEALING WITH THEM.

1.1.1. General complexation details

For NMR studies, the complex was generated *in situ*.

In *[D_3]acetonitrile*: the ligand and $[Fe(H_2O)_6](BF_4)_2$ were combined in the correct stoichiometries using prepared stock solutions to enable accuracy, and administered using micropipette. In *[D_3]nitromethane*: using a prepared stock solution of the ligand, the correct amount was added to a pre-weighed mass of $[Fe(H_2O)_6](BF_4)_2$, and the mixture sonicated, before heating at 55 °C for 30 minutes. In *[D_6]acetone*: using a prepared stock solution of the ligand, the correct amount was added to a pre-weighed mass of $[Fe(H_2O)_6](BF_4)_2$, and the mixture sonicated, before heating at 45 °C for 30 minutes. In *CD_3OD*: using a prepared stock solution of the ligand, the correct amount was added to a pre-weighed mass of $[Fe(H_2O)_6](BF_4)_2$, and the mixture sonicated, before heating at 55 °C for 30 minutes. In *[D_6]DMSO and D_2O*: using a prepared stock solution of the ligand in [D_3]acetonitrile, the correct amount was added to a pre-weighed mass of $[Fe(H_2O)_6](BF_4)_2$, and the mixture sonicated. Either [D_6]DMSO or D_2O were then added however complexation trials were discontinued due to deterioration of the spectra.

1.1.2. NMR pulse programs and parameters

All ^{1}H 1D TOCSY NMR spectra were obtained using the selmlgp pulse program[107] within Topspin. All ^{1}H 2D NOESY NMR spectra were obtained using the noesygpphpp pulse program[108,109] within Topspin. Relaxation time was set to 200 ms. All ^{1}H DOSY NMR spectra were obtained using the convection compensated dstebpgp3s pulse sequence[110,111] within Topspin. The spectra were obtained with δ = 2.0 ms, Δ = 100 ms, and g = 2% – 65% in [D_3]acetonitrile and δ = 2.0 ms, Δ = 100 ms, and g = 2% – 95% in [D_3]nitromethane. Processing was carried out in MestreNova.

1.2. Precursors

1.2.1. A

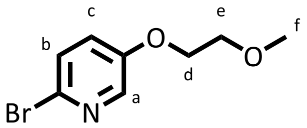

A combination of 2-bromo-5-hydroxypyridine (504 mg, 2.90 mmol), K_2CO_3 (1000 mg, 7.25 mmol) and 2-methoxyethyl p-toluenesulfonate[105] (800 mg, 3.48 mmol) in DMF (40 mL) was heated at 75 °C overnight. After filtration through cotton wool, the solvent was removed under vacuum. The residue was taken up in DCM (50 mL) and washed with water (3 x 100 mL) and brine (50 mL). After removal of the solvent under vacuum, purification through chromatography on silica (DCM to 1:10 acetone/DCM) gave the product as a golden oil (650 mg, 2.83 mmol, 98%). ^{1}H NMR (400 MHz, $CDCl_3$, 298 K) δ: 8.08 (1H, d, J = 2.7 Hz, H_a), 7.36 (1H, d, J = 8.8 Hz, H_b), 7.14 (1H, dd, J = 8.8, 3.2 Hz, H_c), 4.16 – 4.13 (2H, m, H_d), 3.76 – 3.74 (2H, m, H_e), 3.44 (3H, s, H_f). ^{13}C NMR (100 MHz, $CDCl_3$, 298 K) δ: 155.4, 143.2, 136.5, 124.0, 120.4, 70.7, 68.0, 59.4. HR ESI-MS (DCM/methanol) m/z = 231.9970 $[MH]^+$ (calc. for $C_8H_{11}BrO_2$, 231.9968). IR v (cm^{-1}) 2958, 2933, 2872, 1577, 1562, 1449, 1378, 1365, 1270, 1222, 1089, 1009, 824.

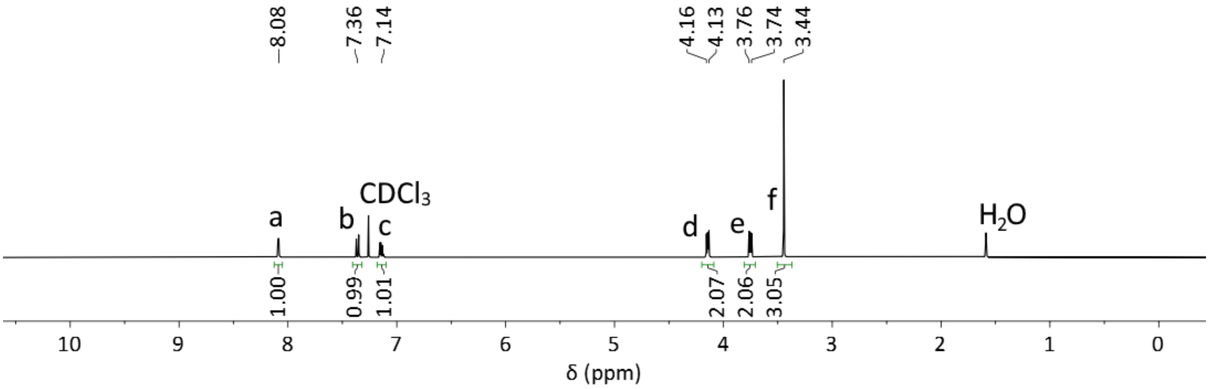

Figure S1.1 ^{1}H NMR spectrum (400 MHz, $CDCl_3$, 298 K) of **A**.

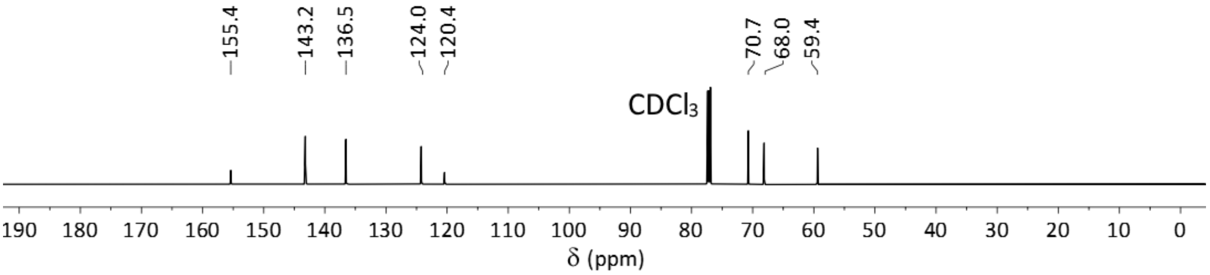

Figure S1.2 ^{13}C NMR spectrum (100 MHz, $CDCl_3$, 298 K) of **A**.

1.2.2. B

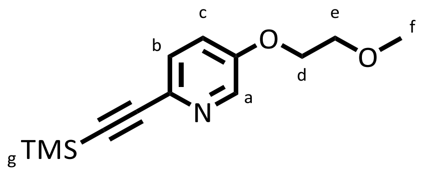

A sealed tube with [Pd(PPh$_3$)$_2$Cl$_2$] (76 mg, 0.11 mmol) and CuI (41 mg, 0.22 mmol) was purged with nitrogen. To the tube was added **A** (500 mg, 2.15 mmol), diisopropylamine (0.6 mL) and TMS-acetylene (317 mg, 459 µL, 3.23 mmol). The tube was capped and heated at 65 °C overnight. After addition of DCM (100 mL) and aqueous 0.1 M EDTA/NH$_4$OH solution (100 mL) and stirring for one hour, the organic phase was washed with water (100 mL) and brine (100 mL). The solvent was removed under vacuum, and purification through chromatography on silica (DCM to 1:10 acetone/DCM) gave the product as a colourless oil (530 mg, 2.12 mmol, 99%). ^{1}H NMR (400 MHz, CDCl$_3$, 298 K) δ: 8.29 (1H, br, H$_a$), 7.39 (1H, d, J = 8.3 Hz, H$_b$), 7.39 (1H, dd, J = 8.7 Hz, 3.0 Hz, H$_c$), 4.18 – 4.16 (2H, m, H$_d$), 3.77 – 3.75 (2H, m, H$_e$), 3.46 (3H, s, H$_f$), 0.25 (9H, s, H$_g$). ^{13}C NMR (100 MHz, CDCl$_3$, 298 K) δ: 154.5, 138.4, 135.3, 127.7, 121.1, 103.6, 93.2, 70.8, 67.8, 59.4, -0.1. HR ESI-MS (DCM/methanol) m/z = 250.1272 [MH]$^+$ (calc. for $C_{13}H_{20}NO_2Si$, 250.1258). IR ν (cm^{-1}) 2975, 2956, 2923, 2896, 2805, 2162, 1582, 1561, 1485, 1466, 1450, 1126, 835, 758.

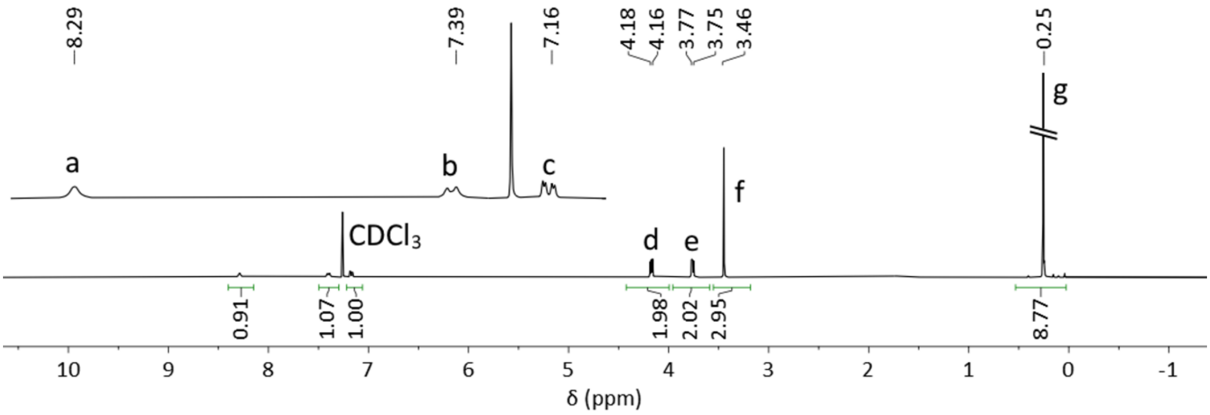

Figure S1.3 ^{1}H NMR spectrum (400 MHz, CDCl$_3$, 298 K) of **B**.

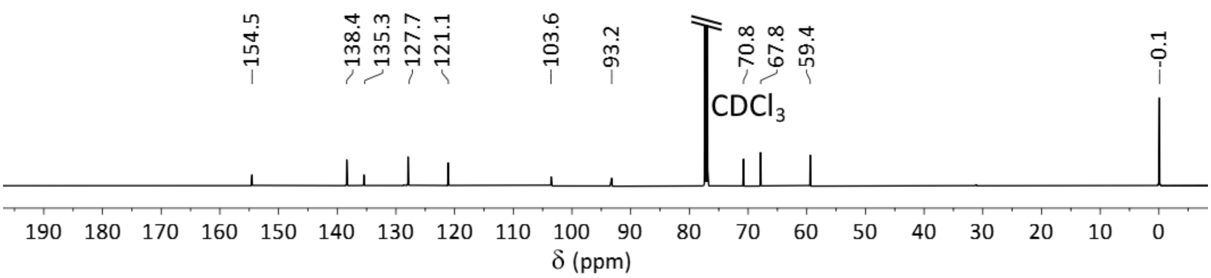

Figure S1.4 ^{13}C NMR spectrum (100 MHz, CDCl$_3$, 298 K) of **B**.

1.3. Ligand

1.3.1. L

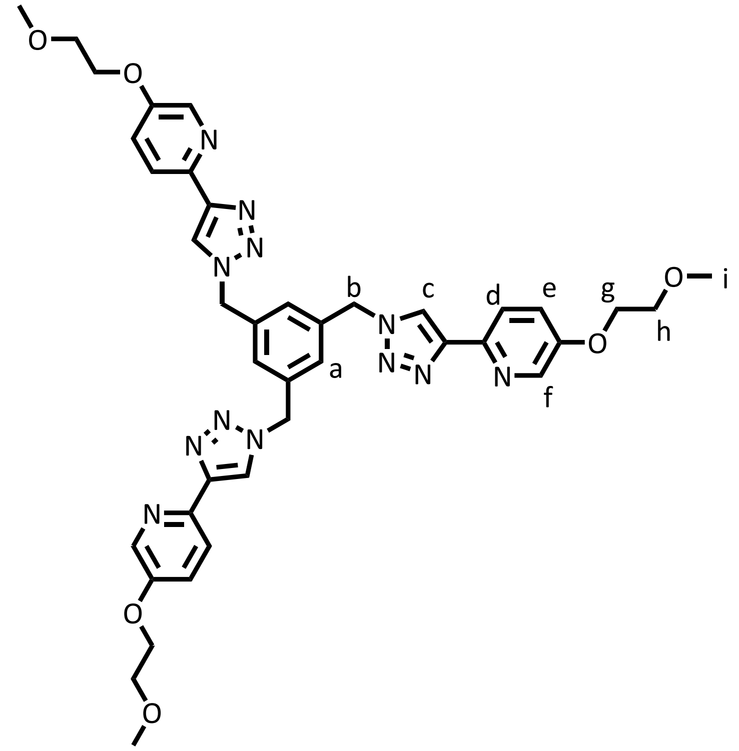

The combination of 1,3,5-tris(bromomethyl)benzene (115 mg, 0.322 mmol) and sodium azide (70 mg, 1.0 mmol) in DMF (7 mL) was stirred for two hours at room temperature. To the mixture was added **B** (250 mg, 1.00 mmol), sodium carbonate (150 mg, 1.58 mmol), $CuSO_4\cdot5H_2O$ (40 mg, 1.0 mmol), sodium ascorbate (60 mg, 1.0 mmol) and water (3 mL) and the mixture was stirred overnight at room temperature. After addition of DCM (100 mL) and aqueous 0.1 M EDTA/NH_4OH solution (100 mL) and stirring for one hour, the organic phase was washed with water (3 × 100 mL) and brine (100 mL). The solvent was removed under vacuum, and purification through chromatography on silica (DCM to 1:2 acetone/DCM to 1:1 acetone/DCM) gave the product as a white powder (210 mg, 0.274 mmol, 85%). ^{1}H NMR (400 MHz, [D$_3$]acetonitrile, 298 K) δ: 8.22 (3H, d, J = 2.4 Hz, H$_f$), 8.11 (3H, s, H$_c$), 7.94 (3H, d, J = 8.7 Hz, H$_d$), 7.34 (3H, dd, J = 8.7, 3.0 Hz, H$_e$), 7.24 (3H, s, H$_a$), 5.55 (6H, s, H$_b$), 4.20 – 4.17 (6H, m, H$_g$), 3.73 – 3.70 (6H, m, H$_h$), 3.37 (9H, s, H$_i$). D (× 10^{-10} m^2 s^{-1}, 400 MHz, [D$_3$]acetonitrile, 298 K) = 7.47. ^{13}C NMR (100 MHz, $CDCl_3$, 298 K) δ: 154.7, 148.6, 142.7, 137.3, 137.0, 128.0, 122.3, 121.5, 121.0, 70.9, 67.9, 59.4, 53.6. HR ESI-MS (DCM/methanol) m/z = 775.3422 [MH]$^+$ (calc. for $C_{39}H_{43}N_{12}O_6$, 775.3423), 797.3238 [MNa]$^+$ (calc. for $C_{39}H_{42}N_{12}O_6Na$, 797.32.43). IR ν (cm^{-1}) 2923, 2883, 2822, 1578, 1480, 1454, 1280, 1238, 1125, 1036, 806, 744.

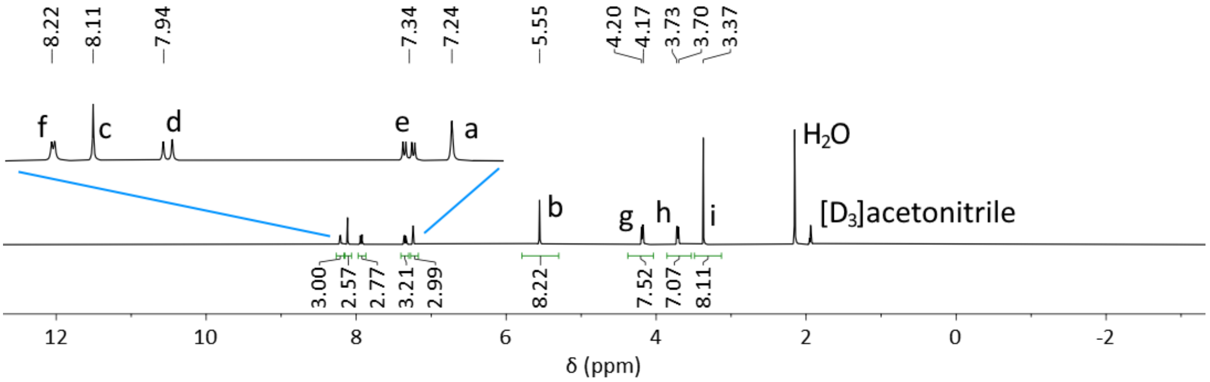

Figure S1.5 ^{1}H NMR spectrum (400 MHz, [D3]acetonitrile, 298 K) of **L**.

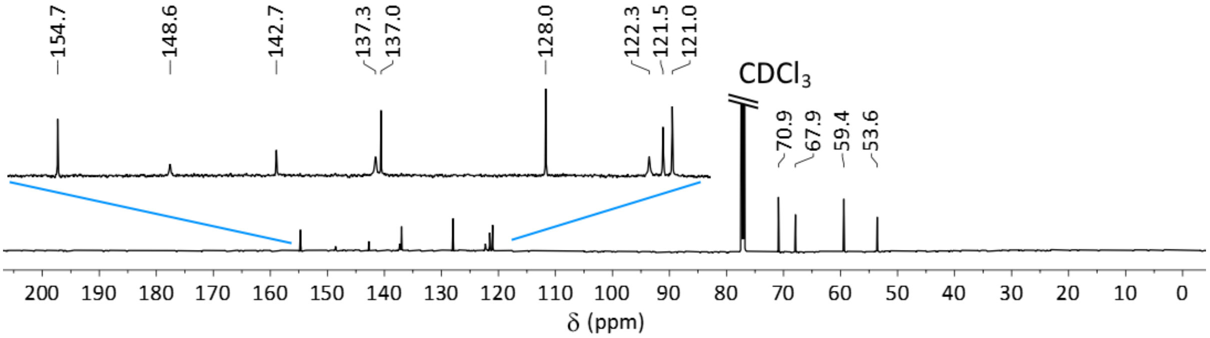

Figure S1.6 ^{13}C NMR spectrum (100 MHz, $CDCl_3$, 298 K) of **L**.

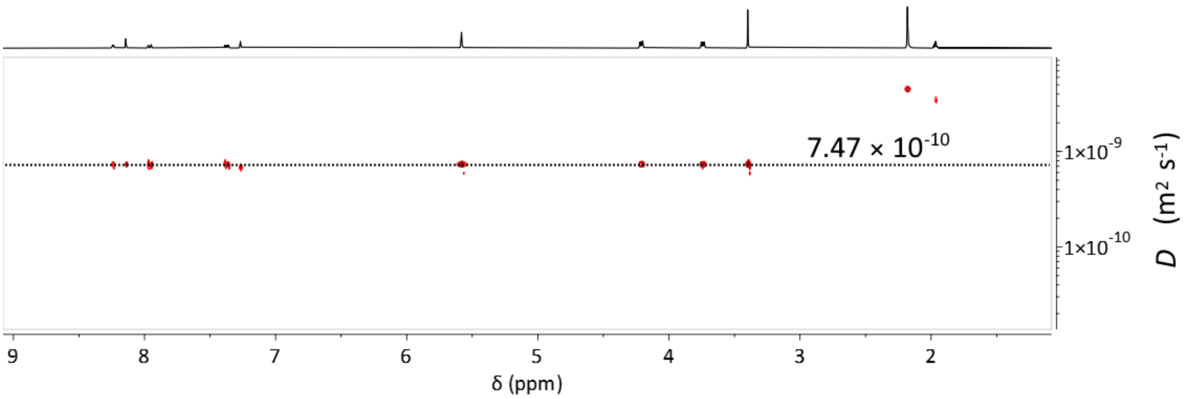

Figure S1.7 ^{1}H DOSY NMR spectrum (400 MHz, [D3]acetonitrile, 298 K) of **L**.

1.4. Complexes

1.4.1. $[Fe_4(L)_4](BF_4)_8$ (*S_4* complex) in acetonitrile

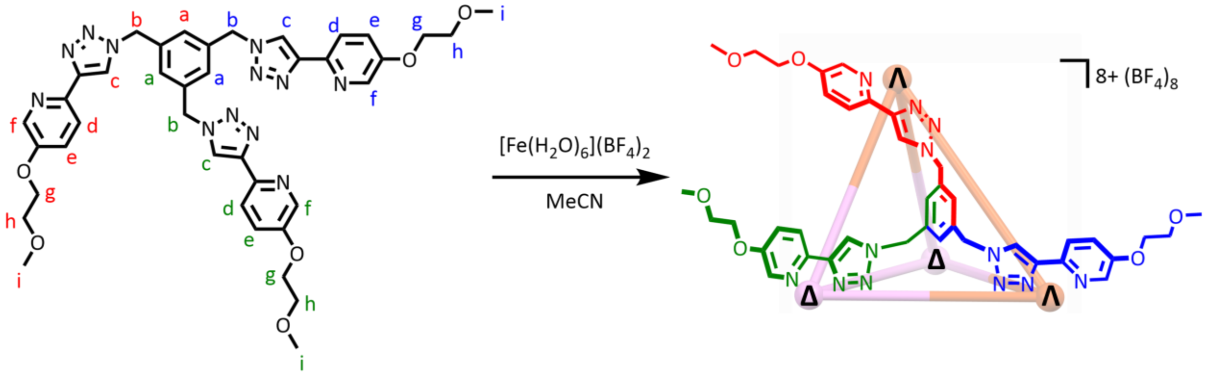

L

***S_4*-$[Fe_4(L)_4](BF_4)_8$**

The combination of **L** (23.6 mg, 30.5 mmol) and $[Fe(H_2O)_6](BF_4)_2$ (10.3 mg, 30.5 mmol) in acetonitrile (2 mL) led to the instantaneous formation of a red/yellow solution. Precipitation with diethyl ether and collection via centrifugation (13K rpm, 10 min) gave the product as a red solid after oven drying (25.6 mg, 6.29 mmol, 82%).

The ^{1}H NMR showed desymmetrisation of the ligand in the complex into three different environments. These have been denoted as Arm 1, Arm 2 and Arm 3, with listings of peaks here identified by their corresponding colours. Arms 1 and 2 share the same chirality at their respective metal ions, Arm 3 has the opposite chirality. The identity of each arm within the solid state structure is given in a subsequent figure (S1.13). In the following listing and in the accompanying figure (S1.8), environment integration is given *per ligand*. ^{1}H NMR (400 MHz, $[D_3]$acetonitrile, 298 K) δ: 8.94 (1H, s, H_c), 8.71 (1H, s, H_c), 8.31 – 8.30 (2H, m, H_c, H_d). 8.22 (1H, d, J = 8.6 Hz, H_d), 8.17 (1H, d, J = 8.9 Hz, H_d), 7.64 – 7.57 (4H, m, H_e, H_e, H_e, H_f), 7.54 (1H, s, H_a), 7.48 (1H, s, H_f), 7.35 (1H, s, H_a), 7.30 (1H, s, H_a), 5.66 (2H, d, J = 17.3 Hz, H_b), 5.53 (1H, d, J = 17.4 Hz, $H_{b'}$), 5.48 – 5.45 (2H, m, $H_{b'}$, H_b), 5.09 (1H, d, J = 14.2 Hz, $H_{b'}$), 5.00 (1H, d, J = 14.4 Hz, H_b), 4.10 – 4.07 (4H, m, H_g, H_g), 4.07 – 4.04 (2H, m, H_g), 3.62 – 3.58 (4H, m, H_h, H_h), 3.58 – 3.55 (2H, m, H_h), 3.28 (3H, s, H_i or H_i), 3.26 (3H, s, H_i or H_i), 3.23 (3H, s, H_i). D ($\times 10^{-10}$ m^2 s^{-1}, 400 MHz, $[D_3]$acetonitrile, 298 K) = 3.97. ^{19}F NMR (376 MHz, $[D_3]$acetonitrile, 298 K) δ: -150.69. HR ESI-MS (acetonitrile, high concentration) m/z = 716.6206 $[Fe_4(\mathbf{L})_4(BF_4)_3]^{5+}$ (calc. for $Fe_4(C_{39}H_{42}N_{12}O_6)_4(BF_4)_3$, 716.6183), 917.5266 $[Fe_4(\mathbf{L})_4(BF_4)_4]^{4+}$ (calc. for $Fe_4(C_{39}H_{42}N_{12}O_6)_4(BF_4)_4$, 917.5240), 1252.3702 $[Fe_4(\mathbf{L})_4(BF_4)5]^{3+}$ (calc. for $Fe_4(C_{39}H_{42}N_{12}O_6)_4(BF_4)_5$, 1252.3668). IR v (cm^{-1}) 3133, 2941, 2888, 2825, 1580, 1448, 1271, 1237, 1027, 1001, 836, 757.

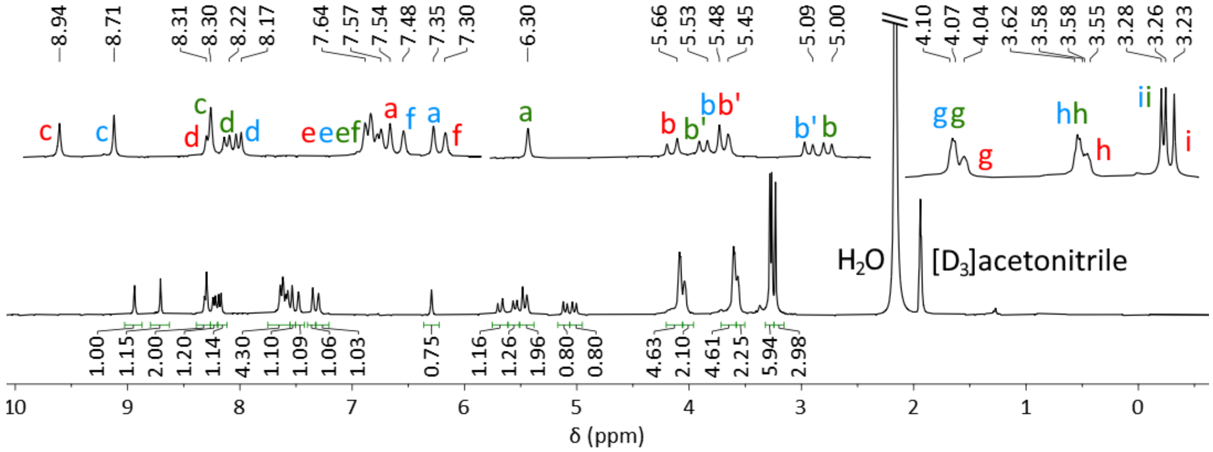

Figure S1.8 ^{1}H NMR spectrum (400 MHz, [D$_3$]acetonitrile, 298 K) of ***S$_4$*-[Fe$_4$(L)$_4$](BF$_4$)$_8$**. Colours: Arm 1, Arm 2, Arm3.

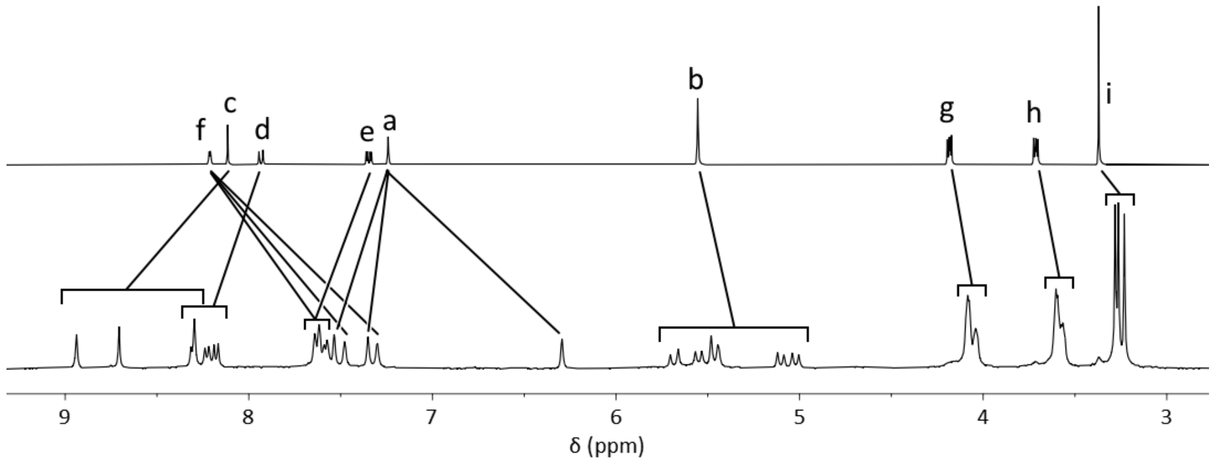

Figure S1.9 Stacked ^{1}H NMR spectra (400 MHz, [D$_3$]acetonitrile, 298 K) of **L** (*top*) and ***S$_4$*-[Fe$_4$(L)$_4$](BF$_4$)$_8$** (*bottom*).

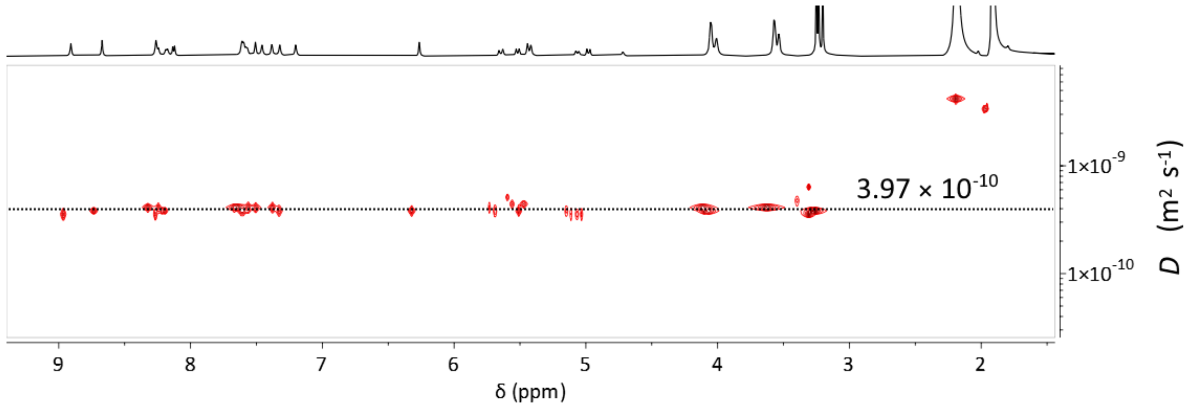

Figure S1.10 ^{1}H DOSY NMR spectrum (400 MHz, [D$_3$]acetonitrile, 298 K) of ***S$_4$*-[Fe$_4$(L)$_4$](BF$_4$)$_8$**.

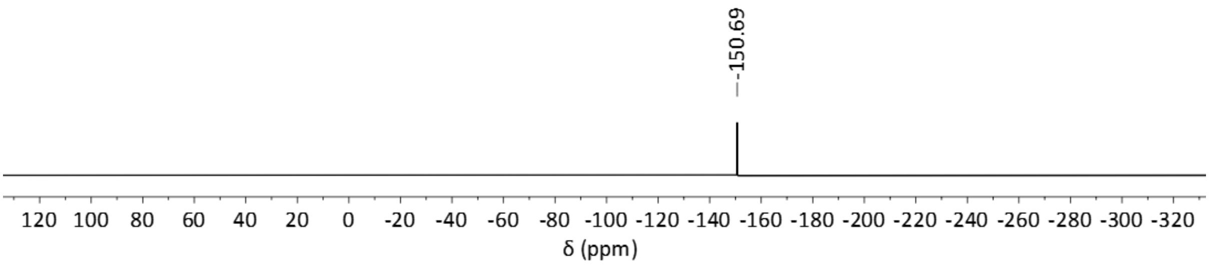

Figure S1.11 ^{19}F NMR spectrum (376 MHz, [D$_3$]acetontrile, 298 K) of ***S$_4$*-[Fe$_4$(L)$_4$](BF$_4$)$_8$**.

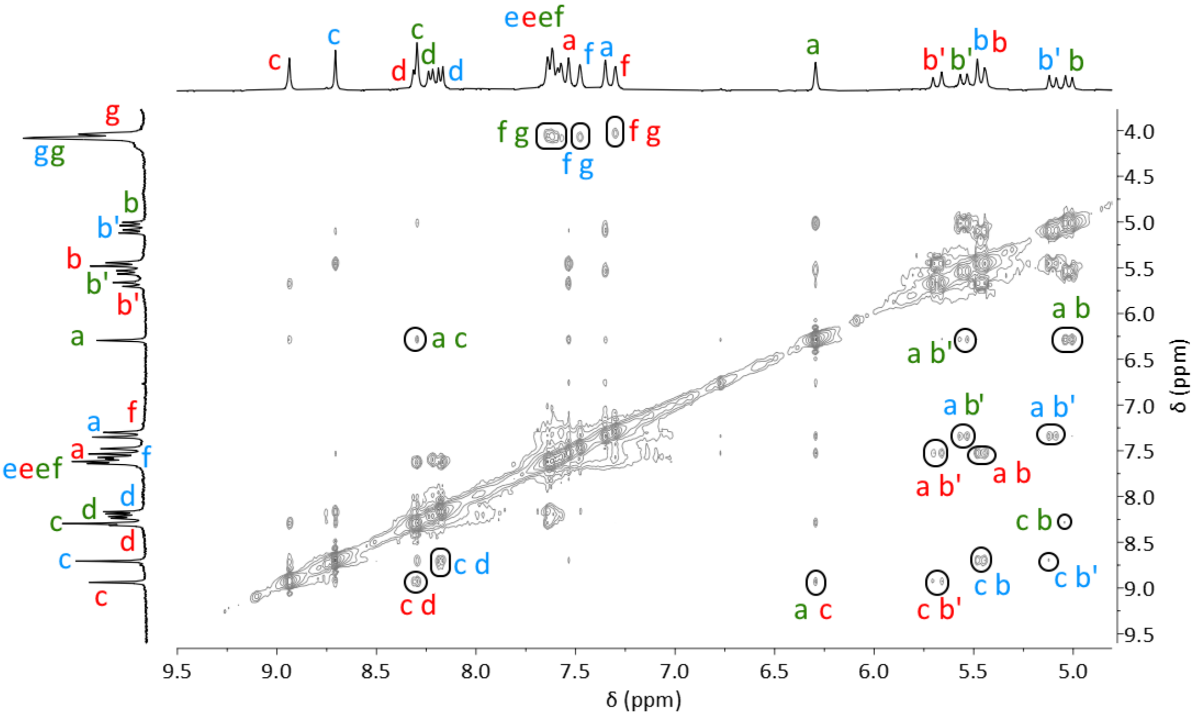

Figure S1.12 ^{1}H NOESY 2D NMR spectrum (400 MHz, [D$_3$]acetonitrile, 298 K, 200 ms) of ***S$_4$*-[Fe$_4$(L)$_4$](BF$_4$)$_8$**. Colours: Arm 1, Arm 2, Arm3.

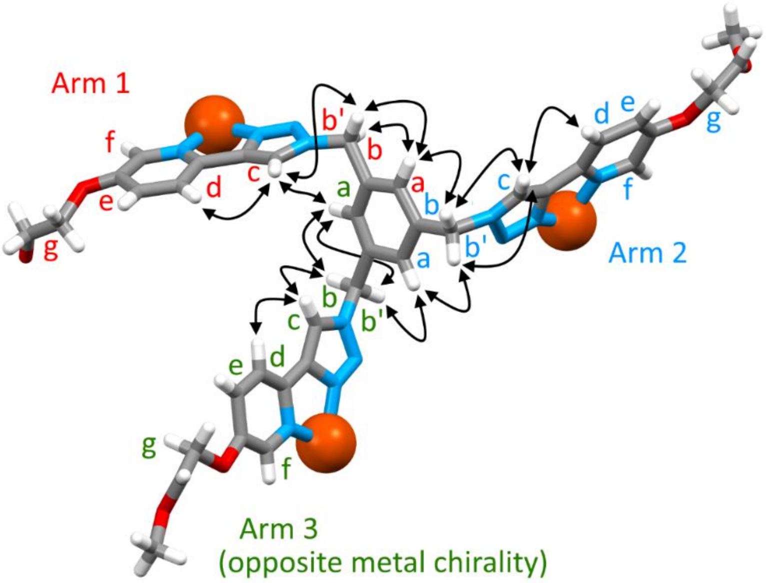

Figure S1.13 View of one of the ligands from the X-ray crystal structure of ***S$_4$*-[Fe$_4$(L)$_4$](BF$_4$)$_8$**, with labelling of hydrogen atoms and observed NOE correlations overlaid.

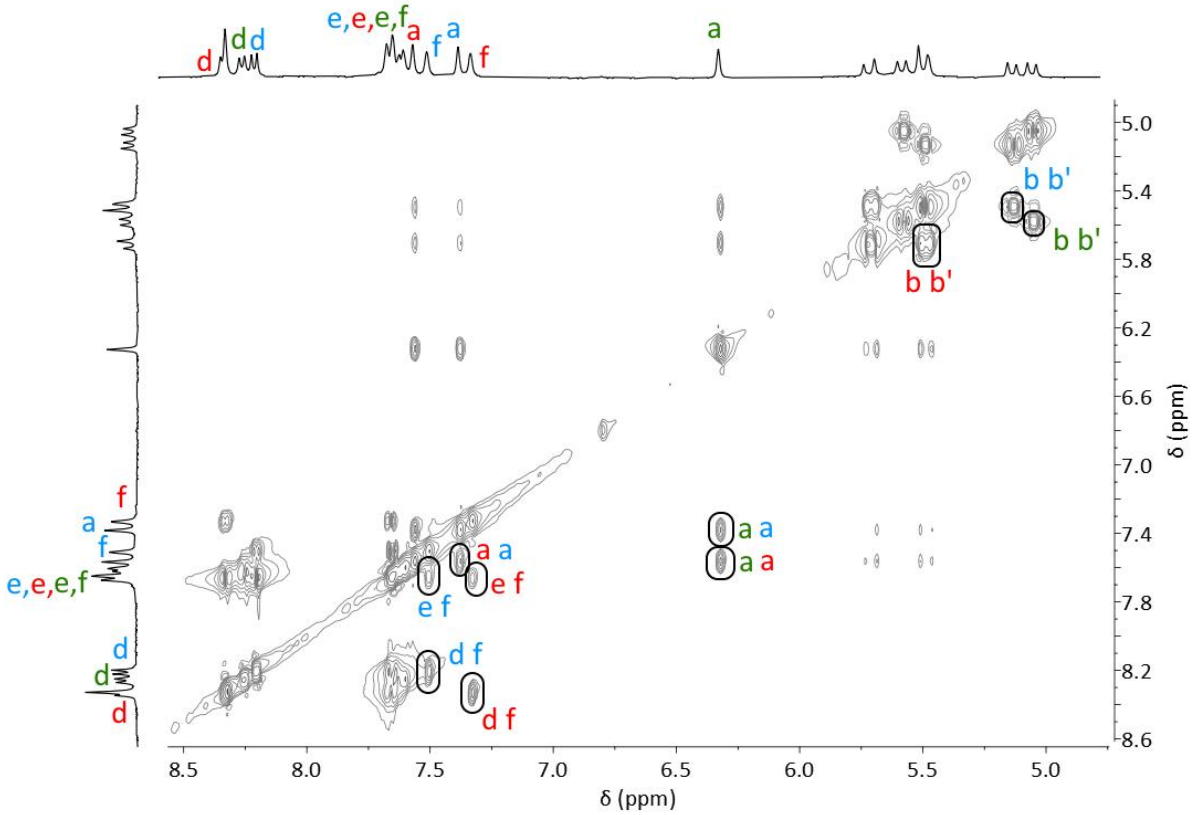

Figure S1.14 Partial ^{1}H TOCSY 2D NMR spectrum (400 MHz, [D$_3$]acetonitrile, 298 K) of ***S$_4$*-[Fe$_4$(L)$_4$](BF$_4$)$_8$**. Colours: Arm 1, Arm 2, Arm3.

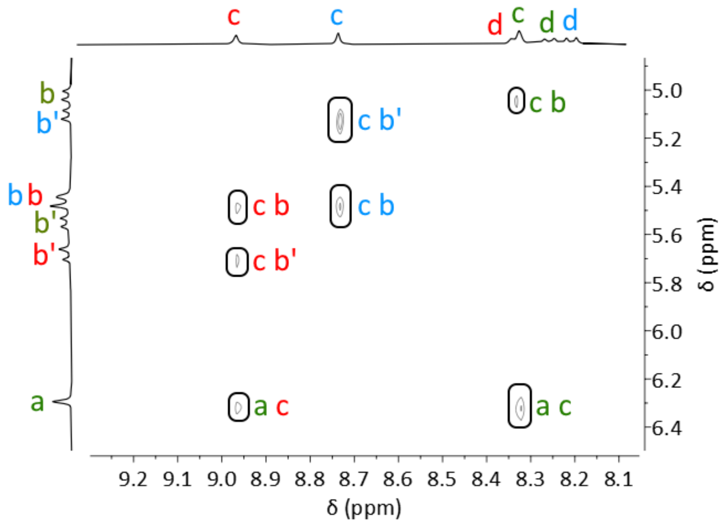

Figure S1.15 Partial ^{1}H TOCSY 2D NMR spectrum (400 MHz, [D$_3$]acetonitrile, 298 K) of ***S$_4$*-[Fe$_4$(L)$_4$](BF$_4$)$_8$**. Colours: Arm 1, Arm 2, Arm3.

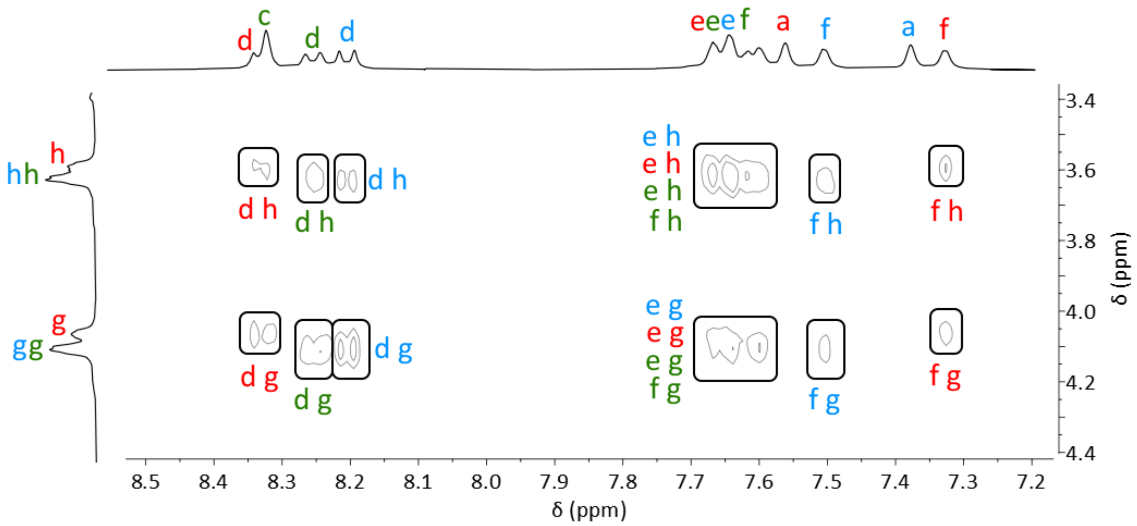

Figure S1.16 Partial ^{1}H TOCSY 2D NMR spectrum (400 MHz, [D_3]acetonitrile, 298 K) of ***S_4*-[$Fe_4(L)_4$](BF_4)$_8$**. Colours: Arm 1, Arm 2, Arm3.

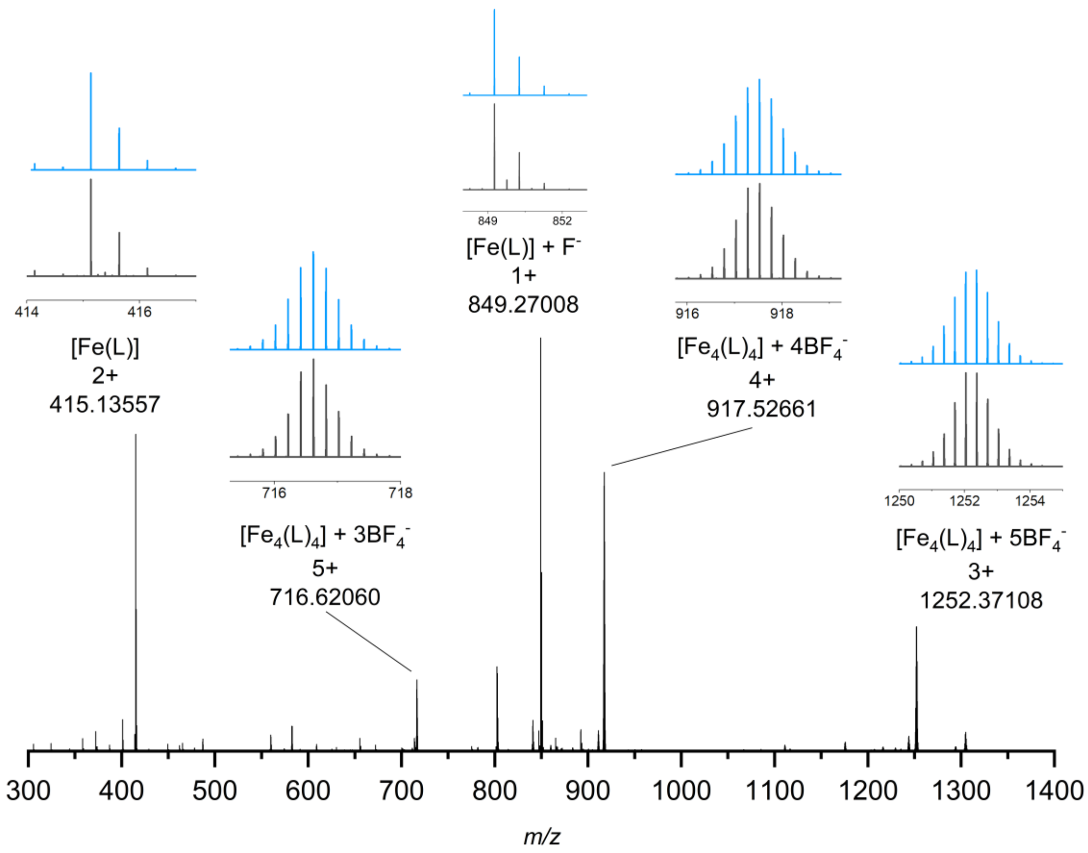

Figure S1.17 Partial high concentration Nanospray MS mass spectrum (acetonitrile) of ***S_4*-[$Fe_4(L)_4$](BF_4)$_8$**.

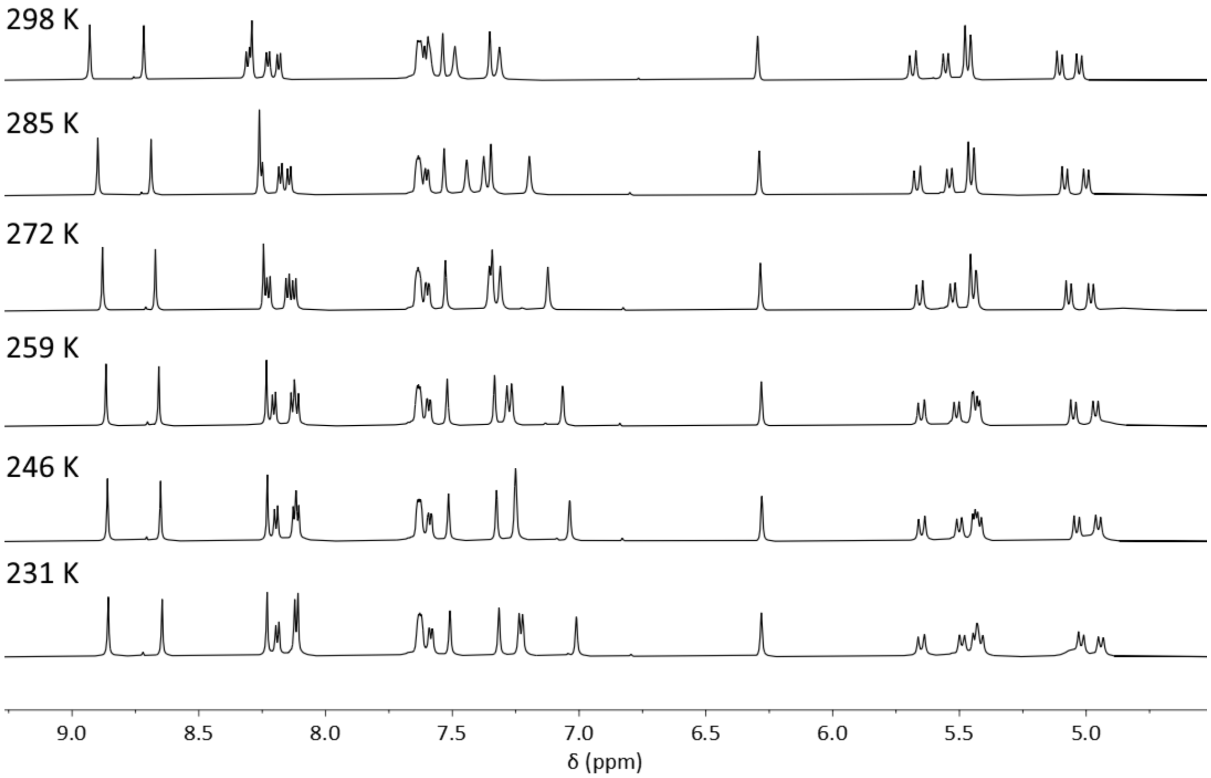

Figure S1.18 Stacked ^{1}H NMR spectra (400 MHz, [D_3]acetonitrile, 298 K) of ***S_4*-[$Fe_4(L)_4$](BF_4)$_8$** at various temperatures from 298 K to 231 K, showing no move to coalescence or desymmetrisation over this temperature.

1.4.2. Complexations in nitromethane

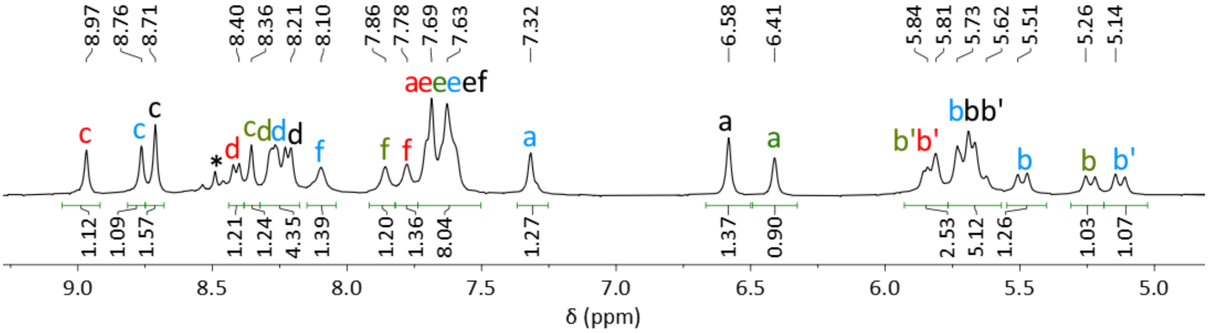

Figure S1.19 ^{1}H NMR spectrum (400 MHz, [D$_3$]nitromethane, 298 K) of the two **[Fe$_4$(L)$_4$](BF$_4$)$_8$** species. Colours: ***S$_4$*-[Fe$_4$(L)$_4$](BF$_4$)$_8$**: Arm 1, Arm 2, Arm3, ***D$_2$*-[Fe$_4$(L)$_4$](BF$_4$)$_8$**: black. *Asterisk denotes common impurity in [D$_3$]nitromethane.

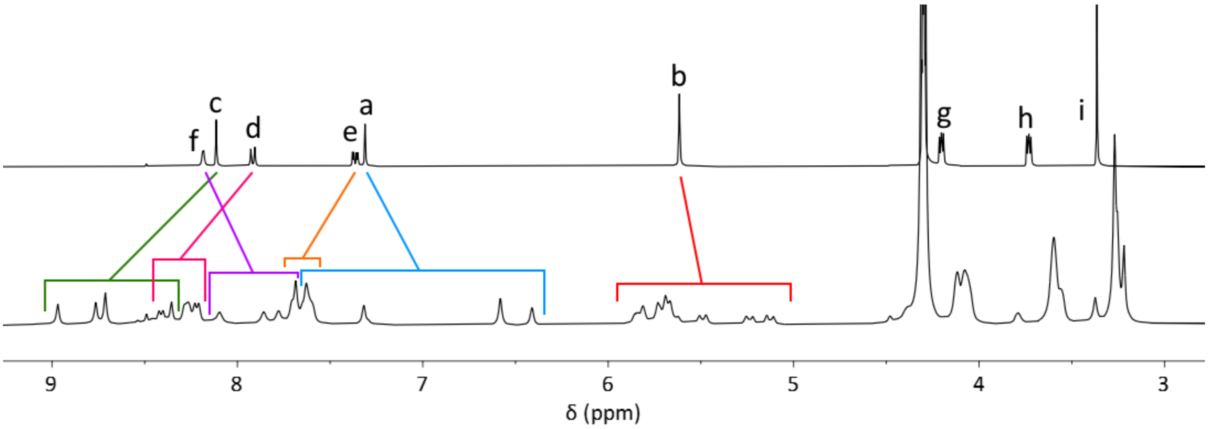

Figure S1.20 Stacked ^{1}H NMR spectra (400 MHz, [D$_3$]nitromethane, 298 K) of **L** (*top*) and the two **[Fe$_4$(L)$_4$](BF$_4$)$_8$** species (*bottom*).

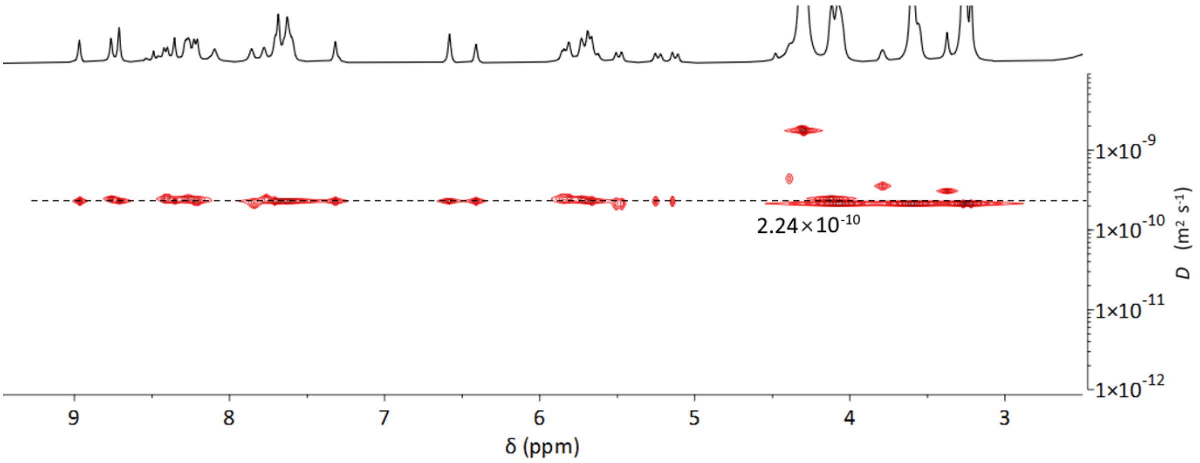

Figure S1.21 ^{1}H DOSY NMR spectrum (400 MHz, [D$_3$]nitromethane, 298 K) of the mixture of the two **[Fe$_4$(L)$_4$](BF$_4$)$_8$** species.

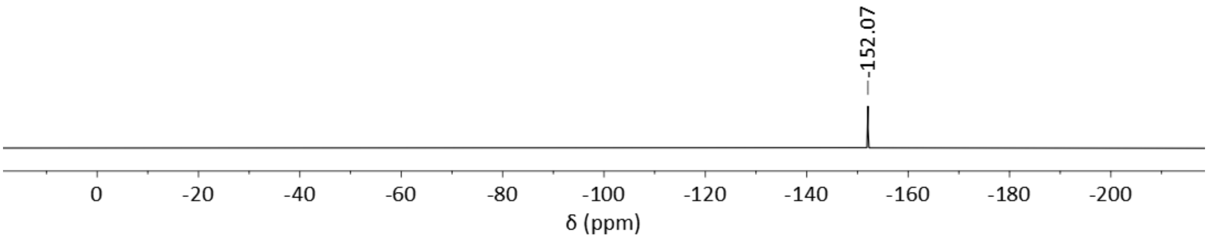

Figure S1.22 ^{19}F NMR spectrum (376 MHz, [D$_3$]nitromethane, 298 K) of the mixture of the two **[Fe$_4$(L)$_4$](BF$_4$)$_8$** species.

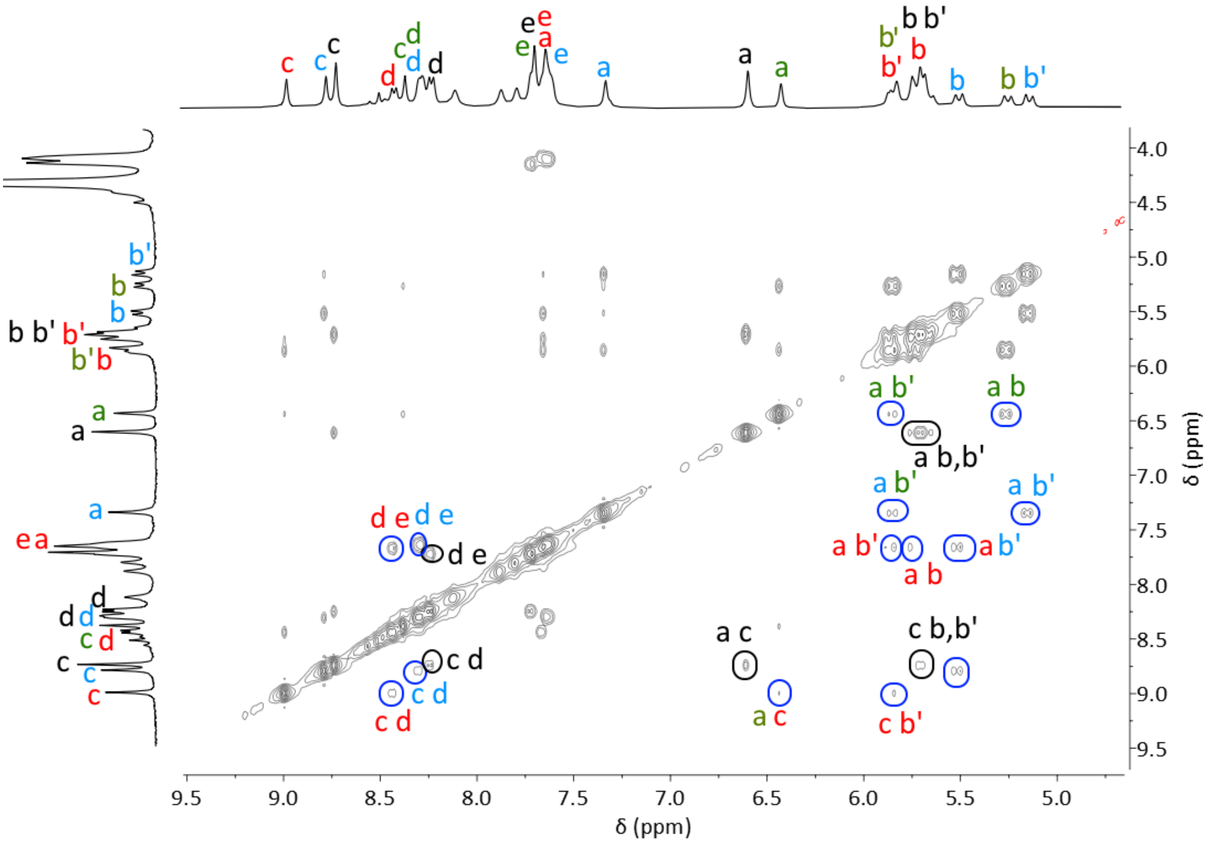

Figure S1.23 ^{1}H NOESY 2D NMR spectrum (400 MHz, [D$_3$]nitromethane, 298 K, 200 ms) of the mixture of the two **[Fe$_4$(L)$_4$](BF$_4$)$_8$** species. Colours: ***S$_4$*-[Fe$_4$(L)$_4$](BF$_4$)$_8$**: Arm 1, Arm 2, Arm3, ***D$_2$*-[Fe$_4$(L)$_4$](BF$_4$)$_8$**: black.

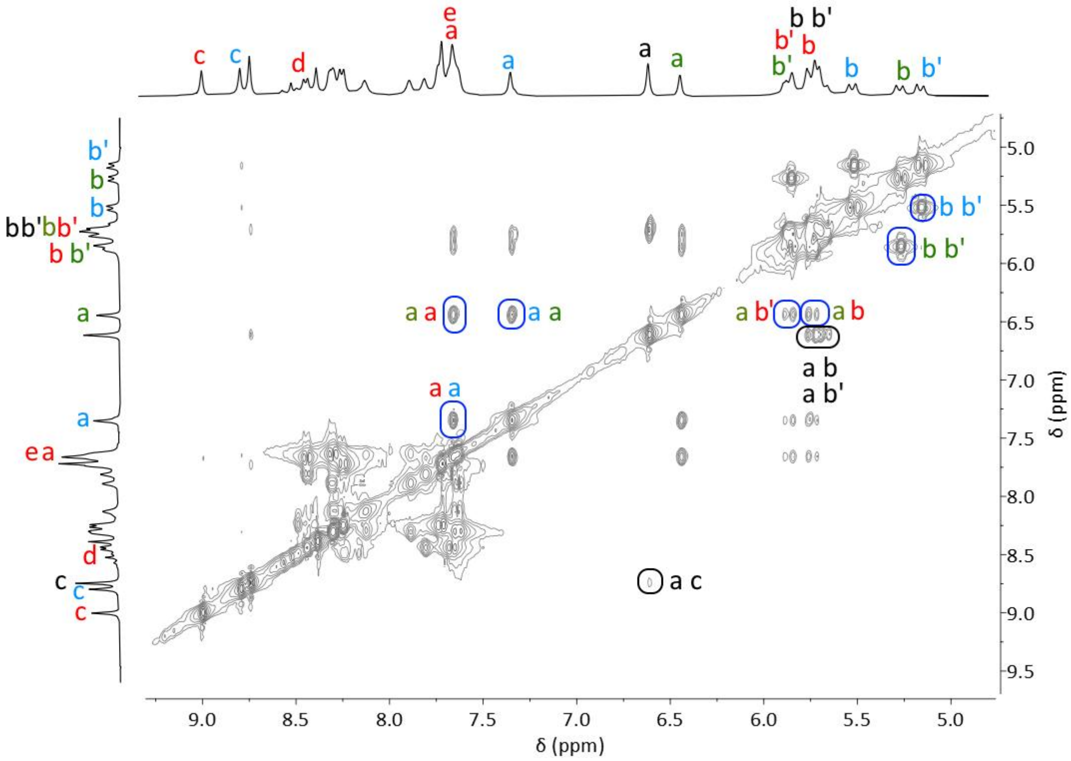

Figure S1.24 Partial ^{1}H TOCSY 2D NMR spectrum (400 MHz, [D$_3$]nitromethane, 298 K) of the mixture of the two **[Fe$_4$(L)$_4$](BF$_4$)$_8$** species. Colours: ***S$_4$*-[Fe$_4$(L)$_4$](BF$_4$)$_8$**: Arm 1, Arm 2, Arm3, ***D$_2$*-[Fe$_4$(L)$_4$](BF$_4$)$_8$**: black.

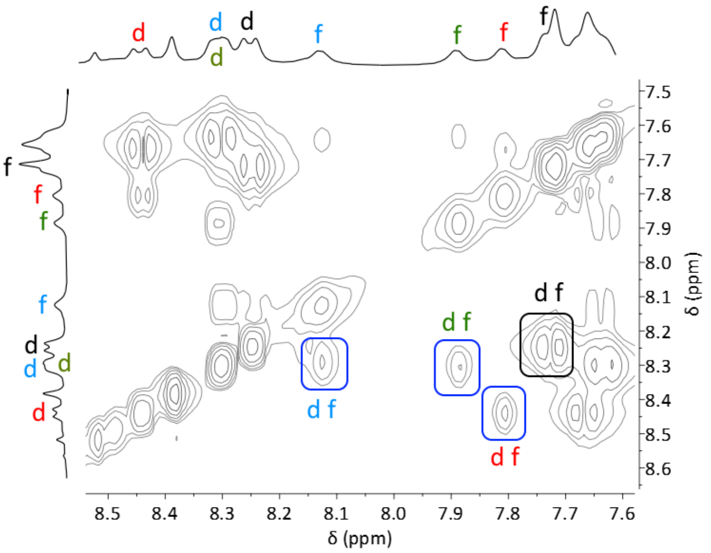

Figure S1.25 Partial ^{1}H TOCSY 2D NMR spectrum (400 MHz, [D$_3$]nitromethane, 298 K) of the mixture of the two **[Fe$_4$(L)$_4$](BF$_4$)$_8$** species. Colours: ***S$_4$*-[Fe$_4$(L)$_4$](BF$_4$)$_8$**: Arm 1, Arm 2, Arm3, ***D$_2$*-[Fe$_4$(L)$_4$](BF$_4$)$_8$**: black.

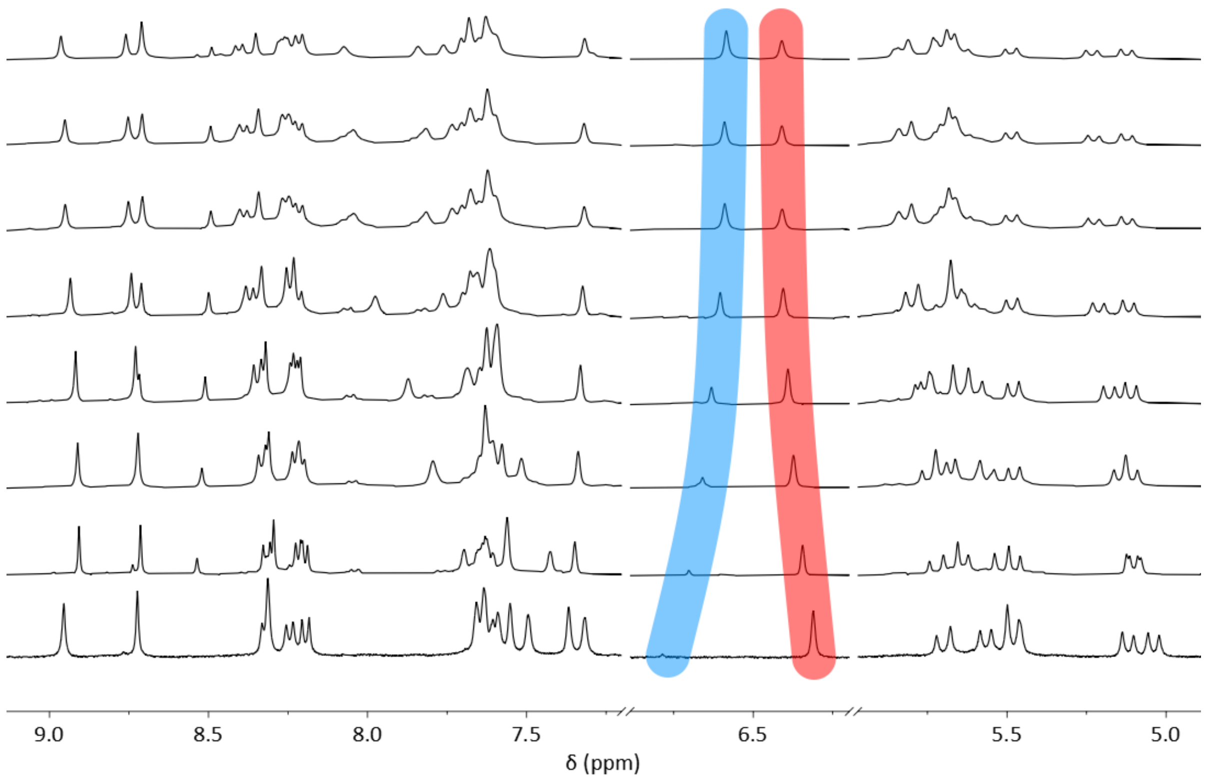

Figure S1.26 Partial ^{1}H NMR stacked spectra (400 MHz, 298 K) of the mixture of the two **[Fe$_4$(L)$_4$](BF$_4$)$_8$** species in different ratios of [D$_3$]nitromethane/[D$_3$]acetonitrile: a) 1.00/0, b) 0.95/0.05, c) 0.90/0.10, d) 0.80/0.20, e) 0.65/0.35, f) 0.50/0.50, g) 0.25/0.75, and h) 0/1.00. *Asterisk denotes common impurity in [D$_3$]nitromethane.

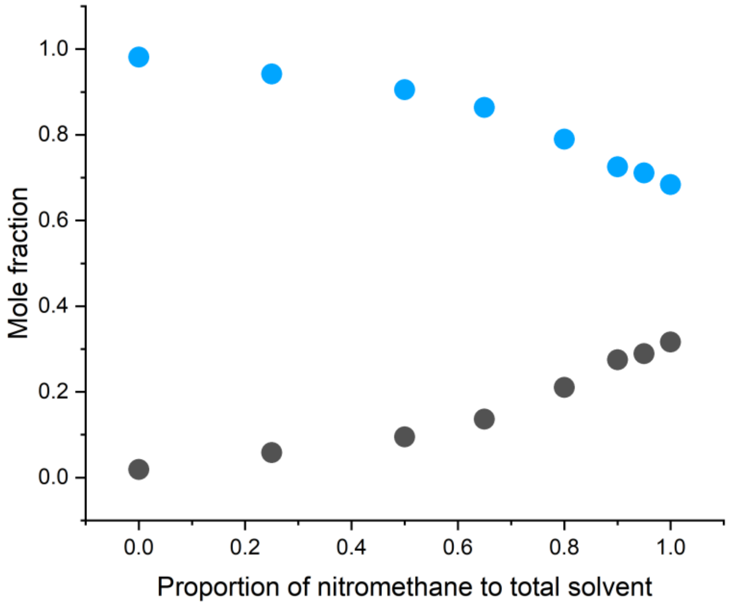

Figure S1.27 Ratio of $\boldsymbol{S_4}$**-[Fe$_4$(L)$_4$](BF$_4$)$_8$** (●) to apparent $\boldsymbol{D_2}$**-[Fe$_4$(L)$_4$](BF$_4$)$_8$** (●) at different ratios of [D$_3$]nitromethane to [D$_3$]acetonitrile.

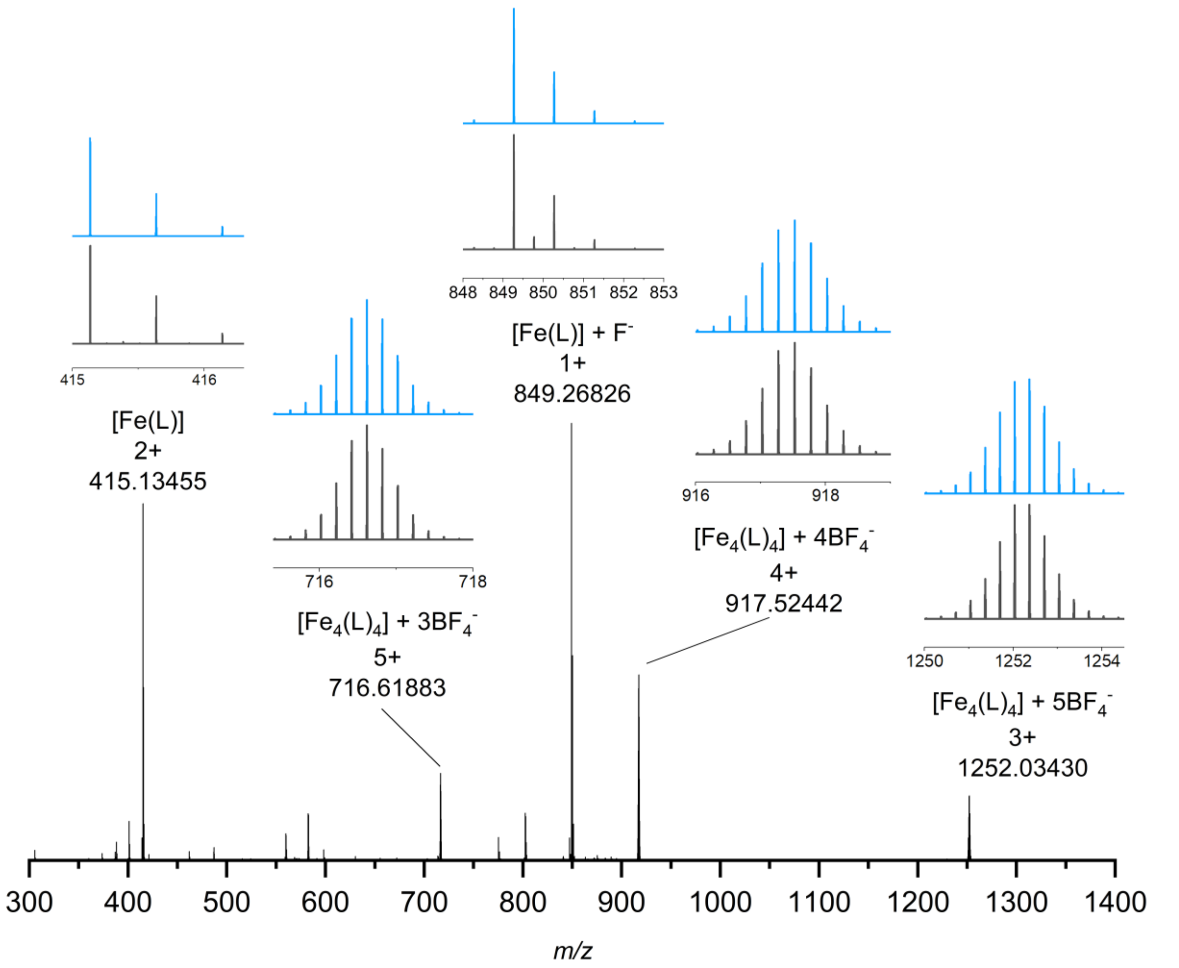

Figure S1.28 Partial high concentration Nanospray MS mass spectrum (nitromethane) of **[Fe$_4$(L)$_4$](BF$_4$)$_8$**. Note the spectrum is essentially identical to that obtained in acetonitrile.

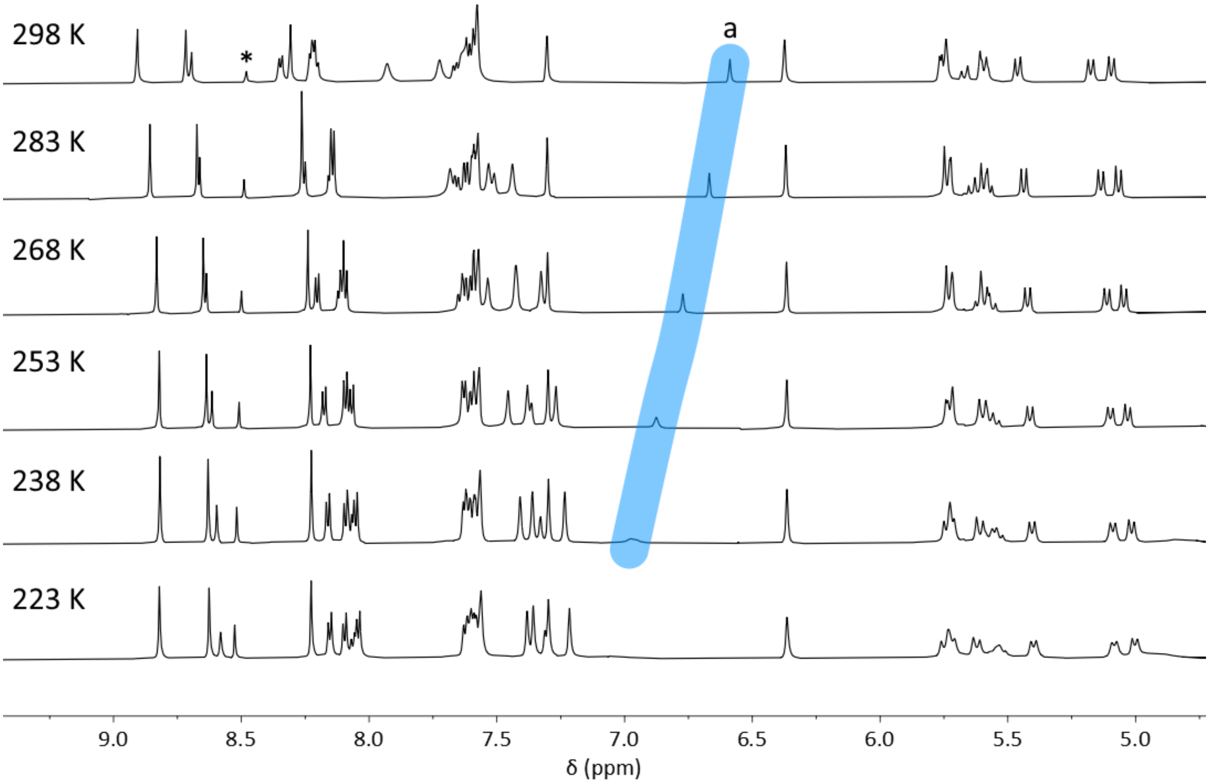

Figure S1.29 Stacked ^{1}H NMR spectra (400 MHz, 1:3 $[D_3]$acetonitrile/$[D_3]$nitromethane) of the two **$[Fe_4(L)_4](BF_4)_8$** species at various temperatures from 298 K to 223 K, showing coalescence of peak H(**a**) at approximately 223 K.

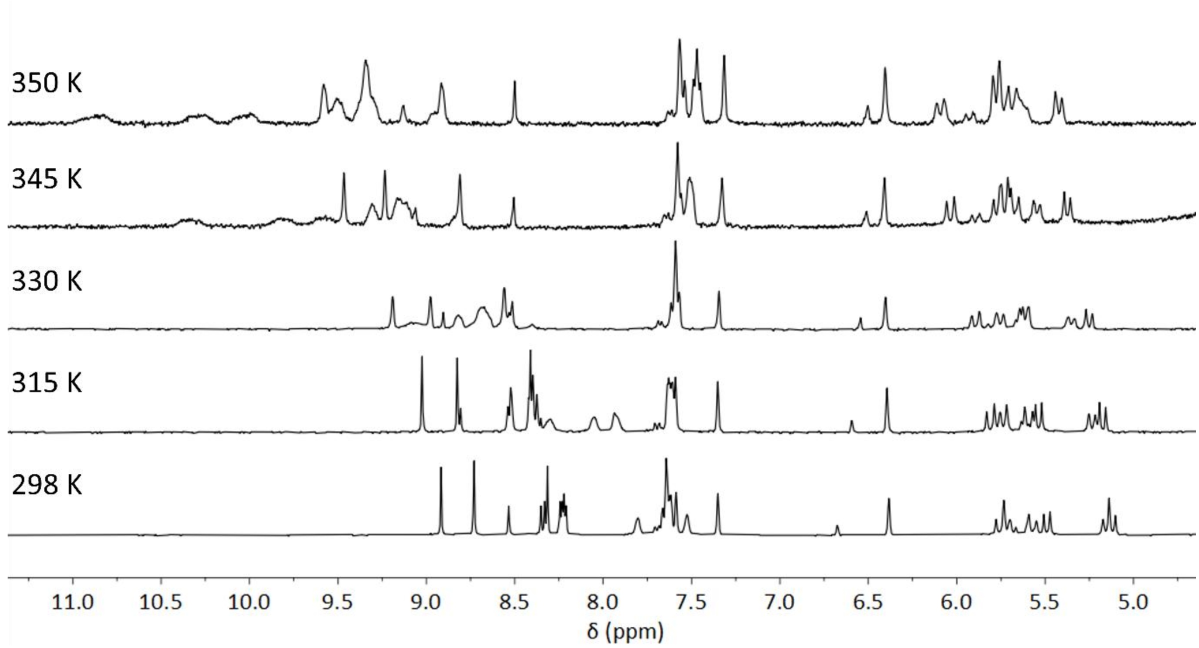

Figure S1.30 Stacked ^{1}H NMR spectra (400 MHz, 1:1 $[D_3]$acetonitrile/$[D_3]$nitromethane) of the two **$[Fe_4(L)_4](BF_4)_8$** species at various temperatures from 298 K to 360 K.

1.4.3. Complexations in [D$_6$]acetone

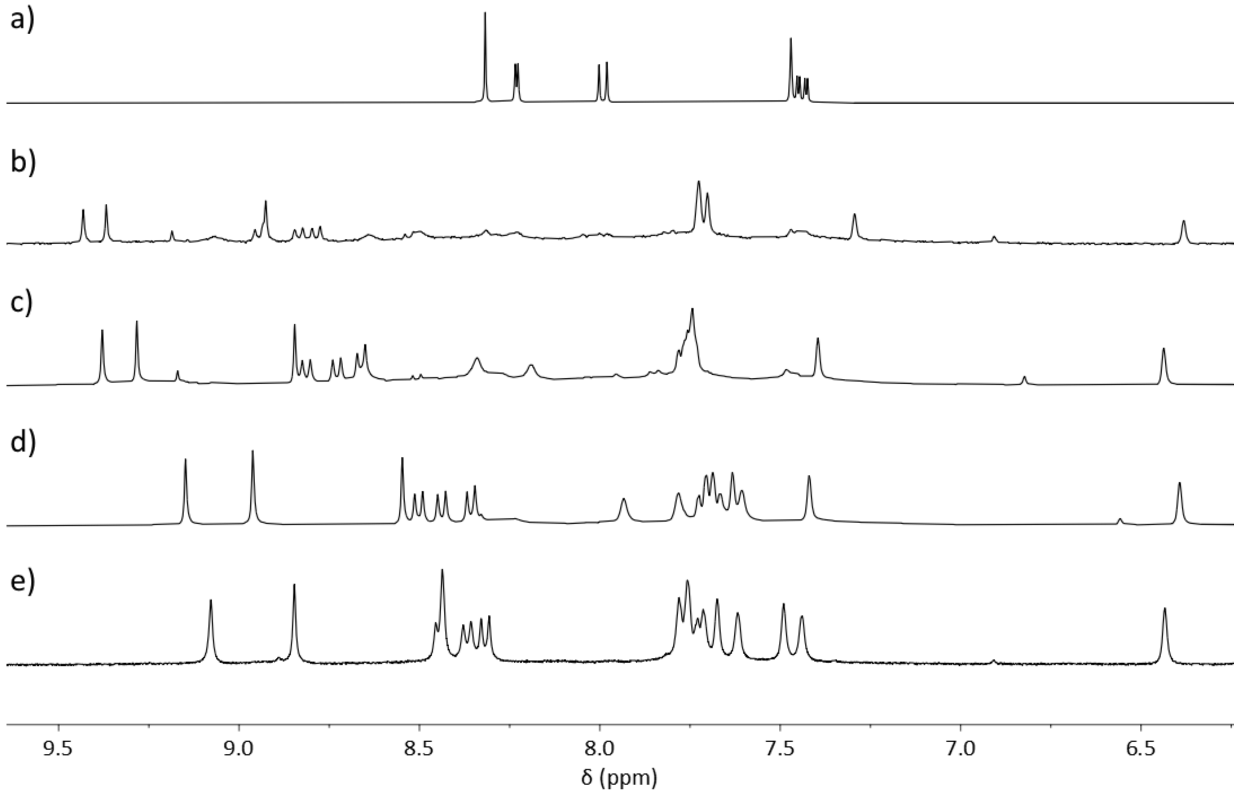

Figure S1.31 Partial ^{1}H NMR stacked spectra (400 MHz, 298 K) of a) **L** in [D$_6$]acetone, and the stoichiometric mixing of **L** and [Fe(H$_2$O)$_6$](BF$_4$)$_2$ in different ratios of [D$_6$]acetone/[D$_3$]acetonitrile: b) 1.00/0, c) 0.90/0.10, d) 0.50/0.50, e) 0/1.00.

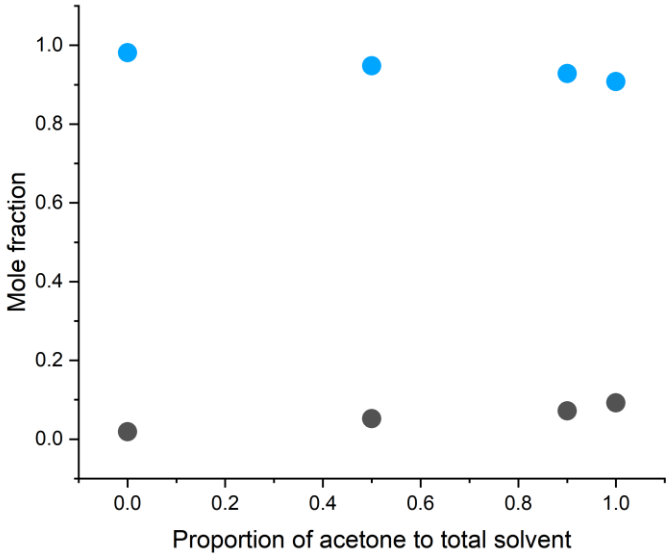

Figure S1.32 Ratio of **S_4-[Fe$_4$(L)$_4$](BF$_4$)$_8$** (●) to apparent ***D_2*-[Fe$_4$(L)$_4$](BF$_4$)$_8$** (●) at different ratios of [D$_6$]acetone to [D$_3$]acetonitrile.

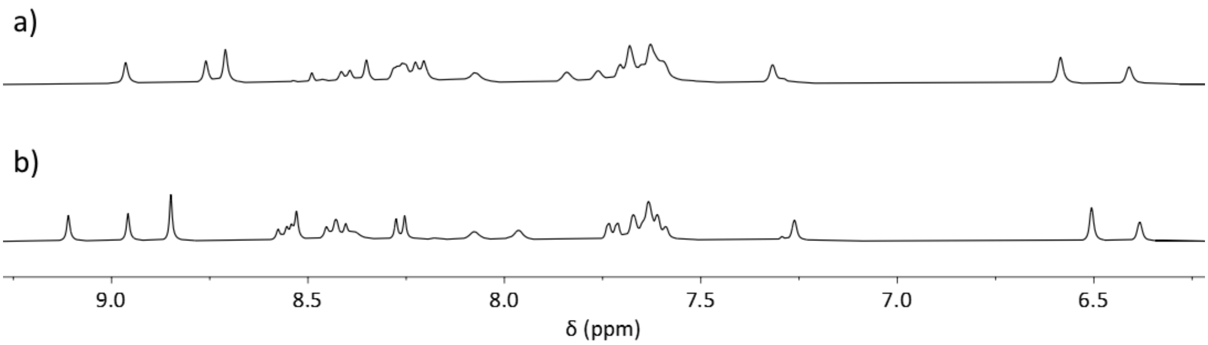

Figure S1.33 Partial ^{1}H NMR stacked spectra (400 MHz, 298 K) of a) the mixture of ***S_4*-[Fe$_4$(L)$_4$](BF$_4$)$_8$** and ***D_2*-[Fe$_4$(L)$_4$](BF$_4$)$_8$** in [D$_3$]nitromethane, and b) the same sample diluted with [D$_6$]acetone to a 50/50 ratio and heated at 48 °C overnight. There was a slight increase in the predominance of ***D_2*-[Fe$_4$(L)$_4$](BF$_4$)$_8$**, from 1:2.16 to 1:1.65 D_2:S_4.

1.4.4. Complexations in [D$_6$]DMSO

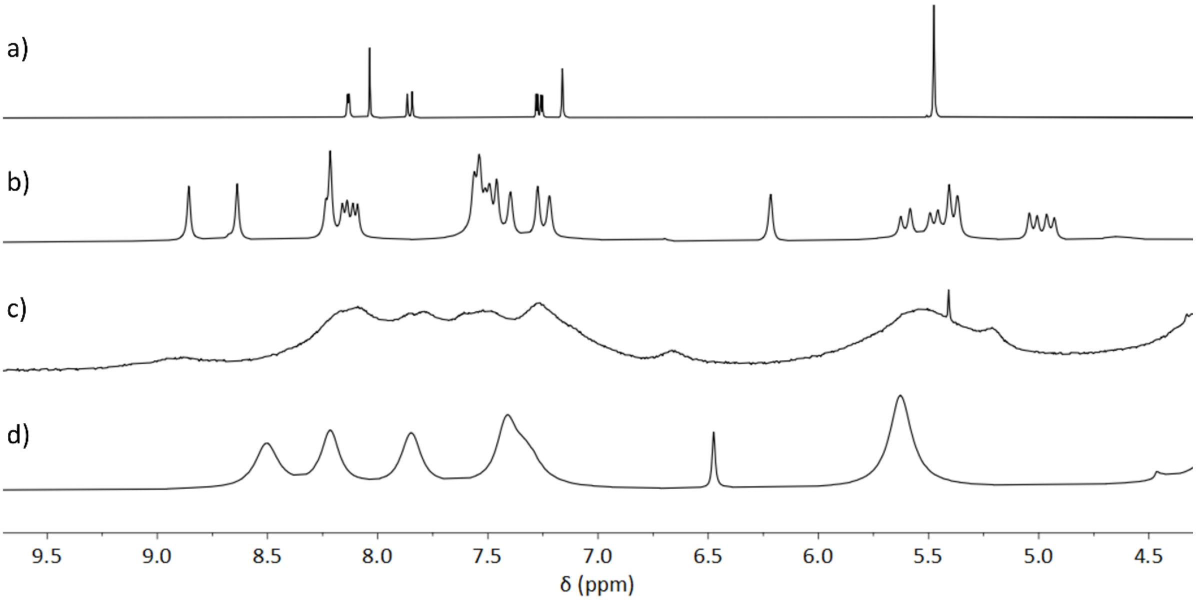

Figure S1.34 Partial ^{1}H NMR stacked spectra (400 MHz, 298 K) of a) **L** in [D$_3$]acetonitrile, and the stoichiometric mixing of **L** and $[Fe(H_2O)_6](BF_4)_2$ in different ratios of [D$_3$]acetonitrile/[D$_6$]DMSO: b) 1.00/0, c) 0.80/0.20, d) 0/1.00.

1.4.5. Complexations in D_2O

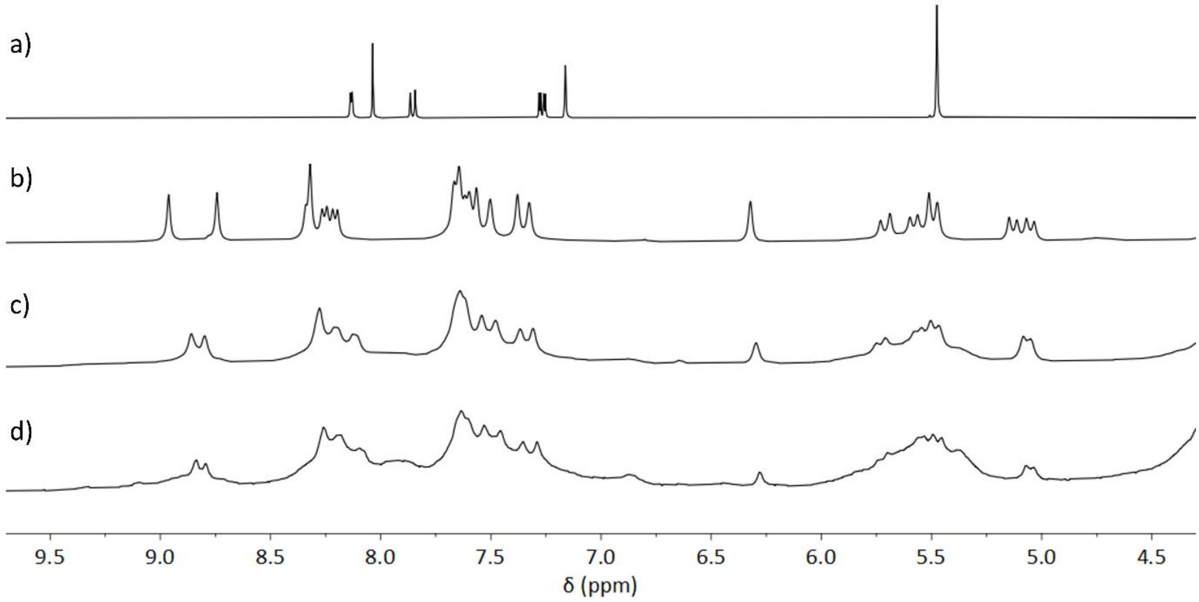

Figure S1.35 Partial ^1H NMR stacked spectra (400 MHz, 298 K) of a) **L** in [D_3]acetonitrile, and the stoichiometric mixing of **L** and $[Fe(H_2O)_6](BF_4)_2$ in different ratios of [D_3]acetonitrile/D_2O: b) 1.00/0, c) 0.80/0.20, d) 0.70/0.30.

1.4.6. Complexations in CD_3OD

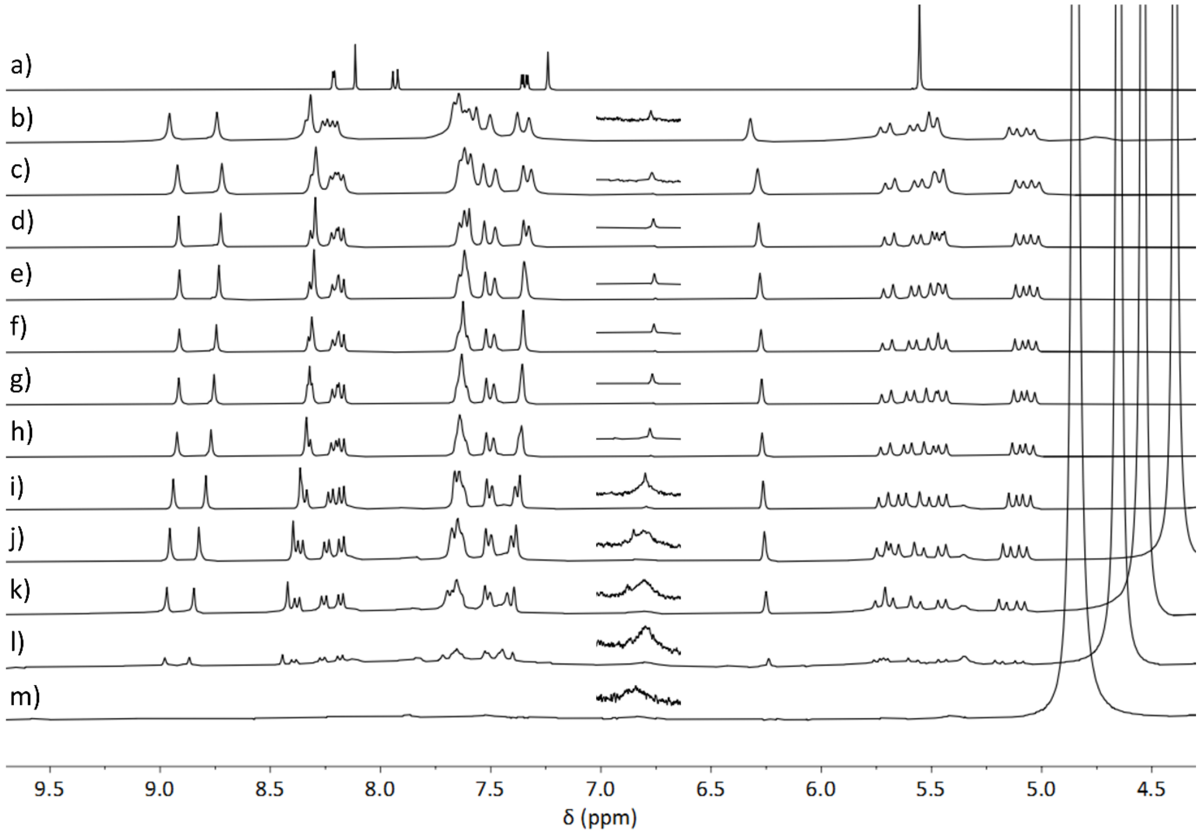

Figure S1.36 Partial ^1H NMR stacked spectra (400 MHz, 298 K) of a) **L** in [D_3]acetonitrile, and the stoichiometric mixing of **L** and $[Fe(H_2O)_6](BF_4)_2$ in different ratios of [D_3]acetonitrile/CD_3OD: b) 1.00/0, c) 0.95/0.05, d) 0.90/0.10, e) 0.80/0.20, f) 0.65/0.35, g) 0.50/0.50, h) 0.35/0.65, i) 0.25/0.75, j) 0.20/0.80, k) 0.10/0.90, l) 0.05/0.95, m) 0/1.00. Inserts at 6.8-6.7 ppm show small fraction of methylene protons relating to $\mathbf{H\cdot(BF_4)_8}$.

2. NMR studies of guest binding

2.1. Procedure for ^{1}H NMR titration experiments

^{1}H NMR binding studies were conducted on a 400 MHz spectrometer at 298 K in CD_3OD:[D_3]acetonitrile (1:1). Initial host concentrations were 2.0 mM (spectrum 1) while solutions of chloride and bromide as their tetrabutylammonium (TBA) salt (100 mM) were added to **M·(BF$_4$)$_8$** in aliquots. Due to solubility issues, azide and nitrite (as their sodium salts) were added as 50 mM solutions. The samples were thoroughly shaken between guest additions, with spectra being recorded at 0, 0.2, 0.4, 0.6, 0.8, 1.0, 1.2, 1.4, 1.6, 1.8, 2.0, 2.5, 3.0, 4.0, 5.0, 7.0 and 10 equivalents of anion. Data were fitted using the *Bindfit* program hosted at Supramolecular.org.[112,113]

2.2. Anion binding data, NMR spectra and isotherms

The following graphs show the data and fitted isotherms used to determine association constants. A global fit was used where possible to determine the association constants, with the resonance movement of peaks H_c (Arm 1), H_c (Arm 2), H_c (Arm 3) and H_b (Arm 1) (Figure S2.1) being recorded and subsequently graphed. All resonances that moved significantly with guest addition related to the acidic triazole protons (H_c), indicating that the anions bound to the exterior of the cage, rather than the internal hydrophobic cavity. There is some movement of the methylene proton resonance H_b, however we attribute this predominantly to secondary inductive electron withdrawing effects caused by interactions between H_c and the anionic guest. While the obtained data for anions Cl^- and Br^- *can* be fitted to a 1:1 H:G binding isotherm, we acknowledge that there are effectively 12 triazole binding sites, and so it should be assumed that a 1:1 H:G binding isotherm is presumably a simplification of the associations between **M·(BF$_4$)$_8$** and the anionic guest. Titrations between N_3^- and NO_2^- and the cage were hindered by decomposition and precipitation, respectively, and as such no further binding analysis was conducted on these guests.

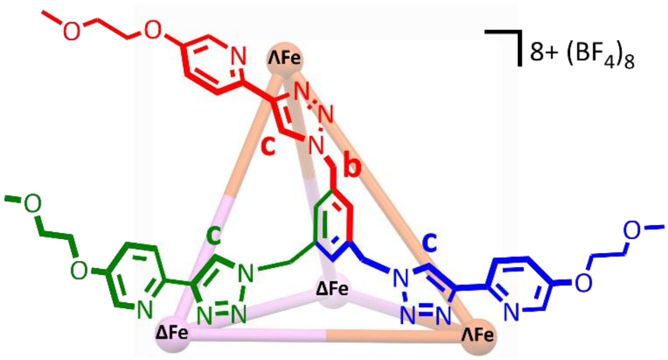

Figure S2.1 Structure of **M·(BF$_4$)$_8$** with triazole C–H resonances H_c, H_c, and H_c, and methylene resonance H_b labelled.

The stacked ^{1}H NMR spectra (Figures S2.2-S2.7) show the titration data obtained from the binding studies between M·(BF$_4$)$_8$ and guests azide and nitrite (as the sodium salt), and chloride and bromide (as the TBA salt). Unless otherwise noted, the stacked ^{1}H NMR spectra each contain 17 spectra corresponding to the equivalents added, with spectrum 1 (the bottom spectrum) corresponding to 0 equivalents of anion while spectrum 17 (the top spectrum) corresponds to 10 equivalents of anion. Full binding data including all fitting parameters are available at the corresponding URLs.

2.3. N_3^- binding to M·(BF4)8

Addition of N_3^- resulted in no observable changes in cage proton resonances, until notable decomposition of the cage structure at 1.0 equivalents of anion. Due to decomposition of **M·(BF$_4$)$_8$** with increased additions of N_3^-, this titration was not continued past 1.5 equivalents of anion.

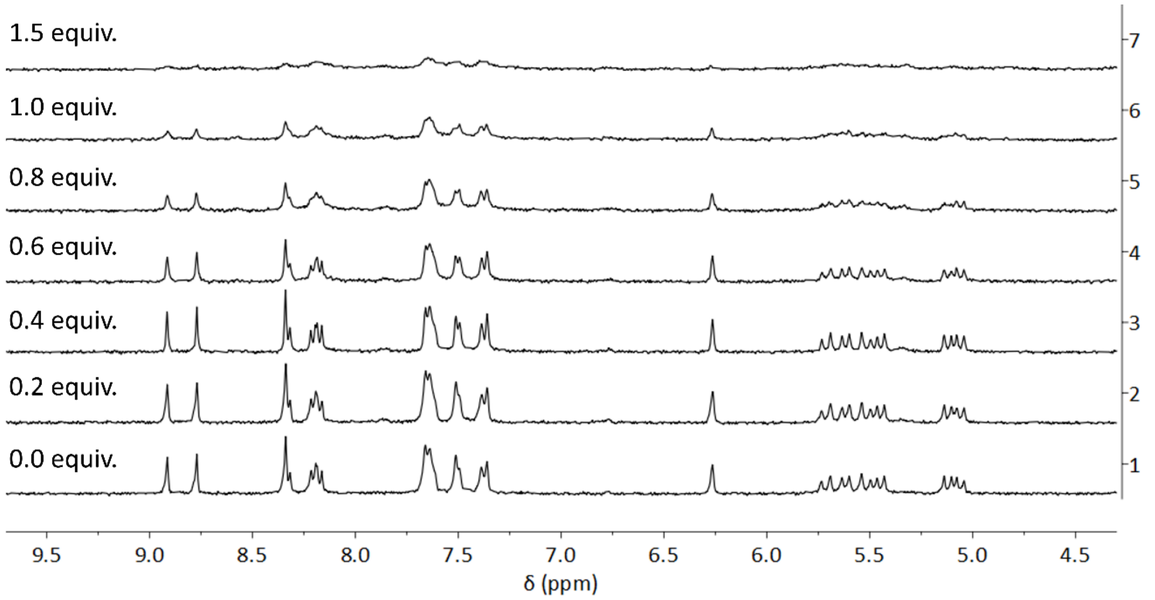

Figure S2.2 Partial ^{1}H NMR spectra of **M·(BF$_4$)$_8$** upon the addition of increasing equivalents of Na·N_3 (0 to 1.5 equivalents) (298 K, 400 MHz, 1:1 [D$_3$]acetonitrile:CD_3OD).

2.4. NO_2^- binding to M·(BF4)8

Addition of NO_2^- resulted in minimal observable changes in cage proton resonances. Due to precipitation of **M·(BF$_4$)$_8$** with increased additions of NO_2^-, this titration was not continued past 3.0 equivalents of anion.

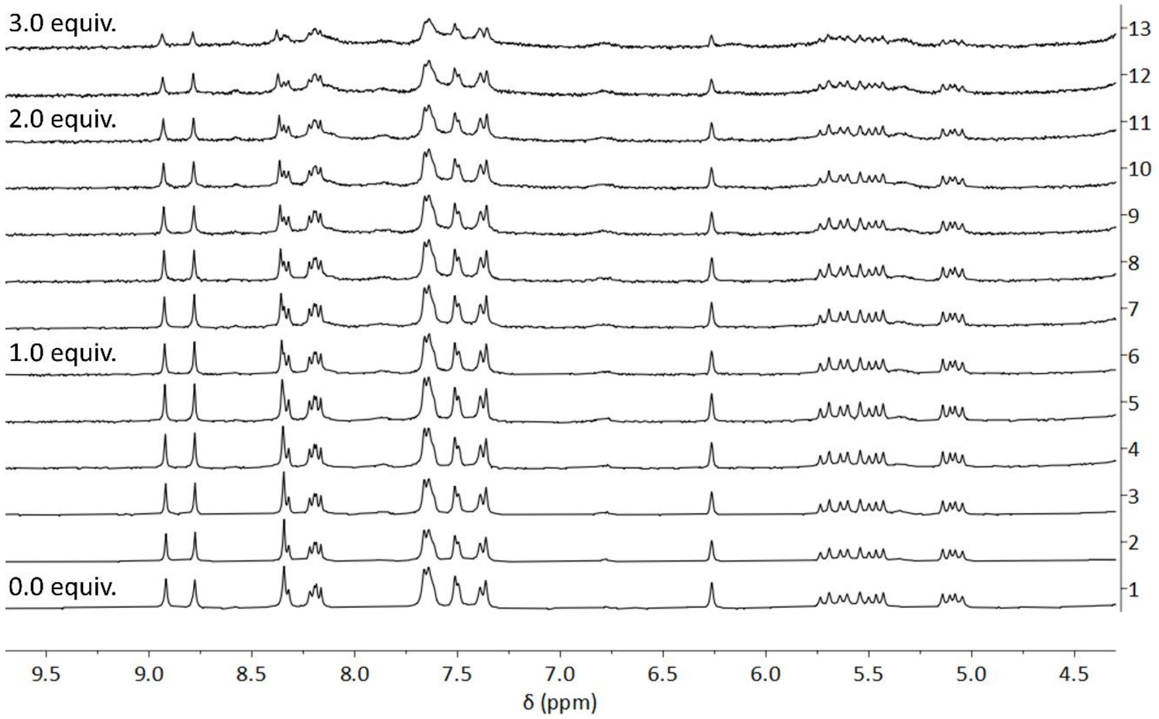

Figure S2.3 Partial ^{1}H NMR spectra of **M·(BF$_4$)$_8$** upon the addition of increasing equivalents of Na·NO_2 (0 to 3.0 equivalents) (298 K, 400 MHz, 1:1 [D$_3$]acetonitrile:CD_3OD).

2.5. Cl^- binding to M·$(BF_4)_8$

Addition of Cl^- resulted in small observable changes in C–H resonances H_c, H_c, and H_c, indicating that despite the smaller size of this anion, it fails to bind within the interior of the cage, binding weakly instead to the exterior triazole protons. The data were fitted to a 1:1 binding isotherm with K = 68 ± 1 M^{-1}.

Bindfit data: http://app.supramolecular.org/bindfit/view/580202a6-4ad6-4e2a-90f9-092b918cd527

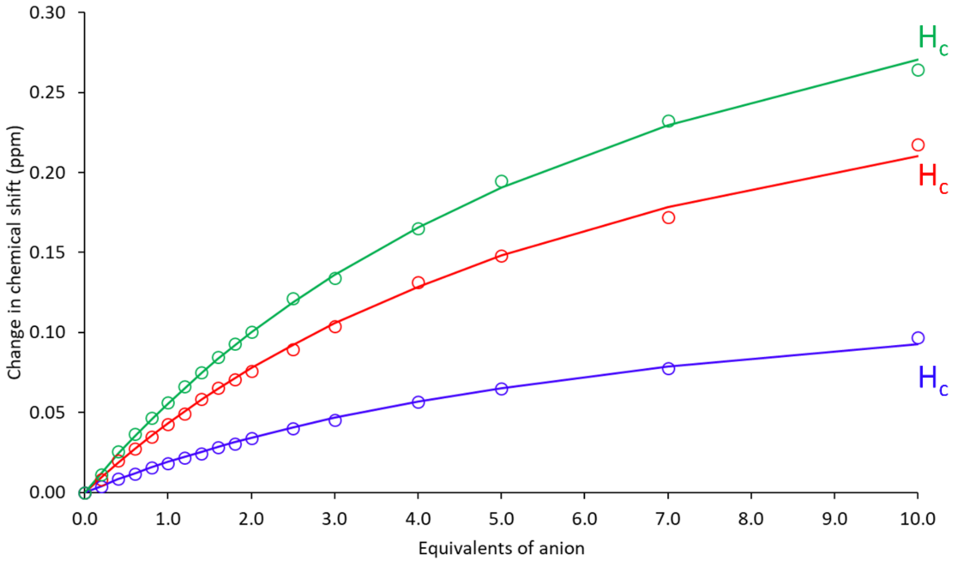

Figure S2.4 Movement of triazole C–H resonances of **M·$(BF_4)_8$** upon the addition of TBA·Cl in $[D_3]$acetonitrile:CD_3OD (1:1). Dots represent observed data points and line represents 1:1 binding isotherm calculated using *Bindfit*.[112,113]

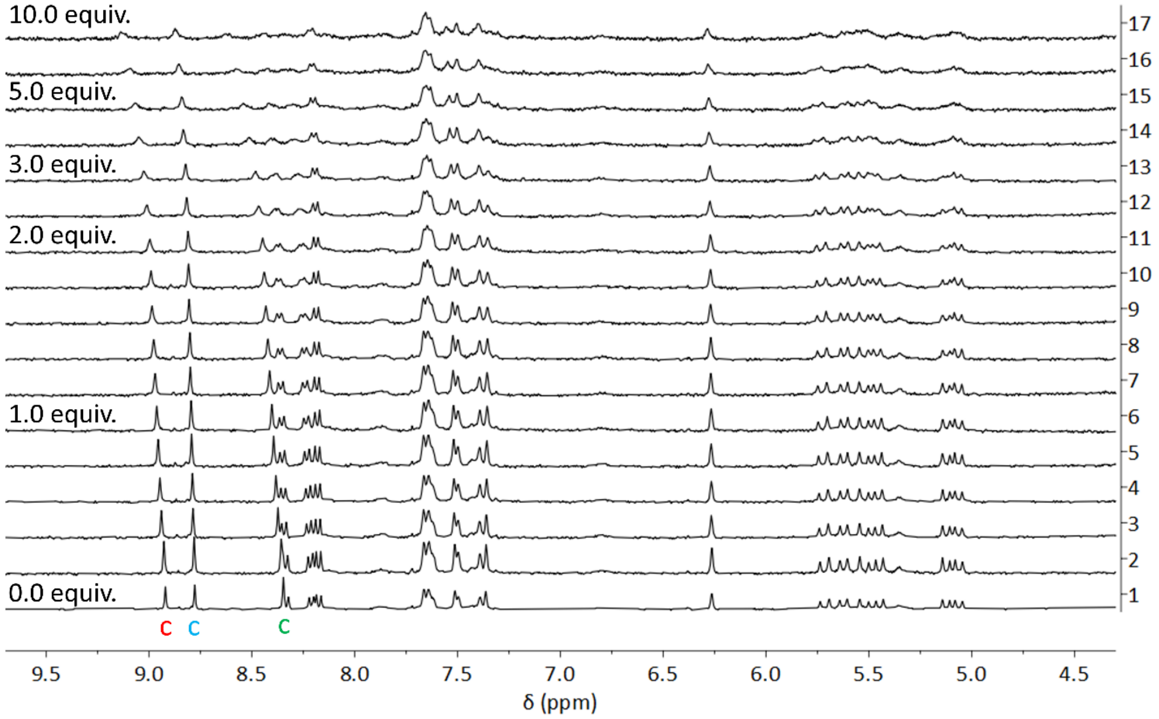

Figure S2.5 Partial ^1H NMR spectra of **M·$(BF_4)_8$** upon the addition of increasing equivalents of TBA·Cl (0 to 10.0 equivalents) (298 K, 400 MHz, 1:1 $[D_3]$acetonitrile:CD_3OD).

2.6. Br⁻ binding to M·(BF$_4$)$_8$

2.6.1. Br⁻ binding to M·(BF$_4$)$_8$ at 298 K

Addition of Br⁻ resulted in moderate downfield shifts in the triazole C–H resonances H_c, H_c, and H_c, as well as the methylene resonance H_b, indicating that the bromide anion also fails to bind within the interior of the cage, binding instead to the exterior triazole protons. The data were fitted to a 1:1 binding isotherm with K = 138 ± 1 M^{-1}.

Bindfit data: http://app.supramolecular.org/bindfit/view/fb0272cd-b83b-4cb0-aaf8-f42ed3130426

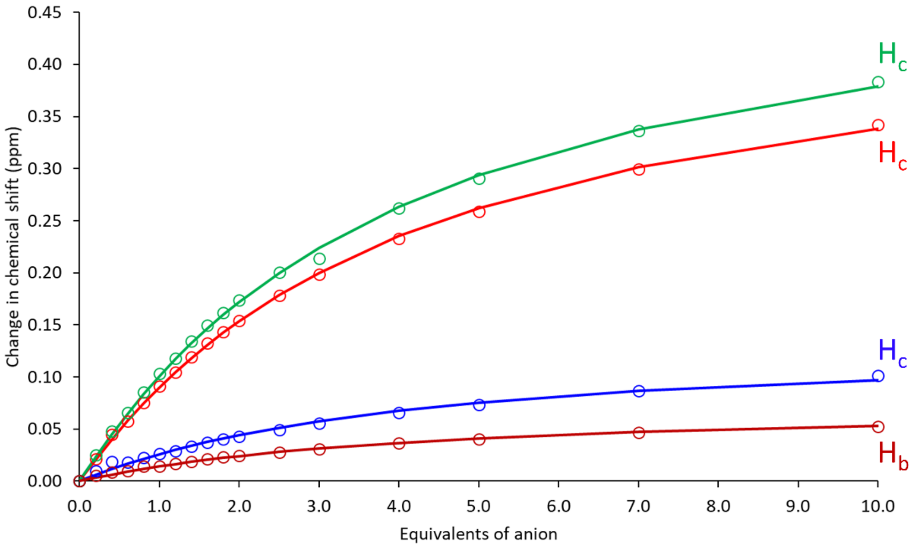

Figure S2.6 Movement of triazole and methylene C–H resonances of M·(BF$_4$)$_8$ upon the addition of TBA·Br in [D$_3$]acetonitrile:CD$_3$OD (1:1). Dots represent observed data points and line represents 1:1 binding isotherm calculated using *Bindfit*.[112,113]

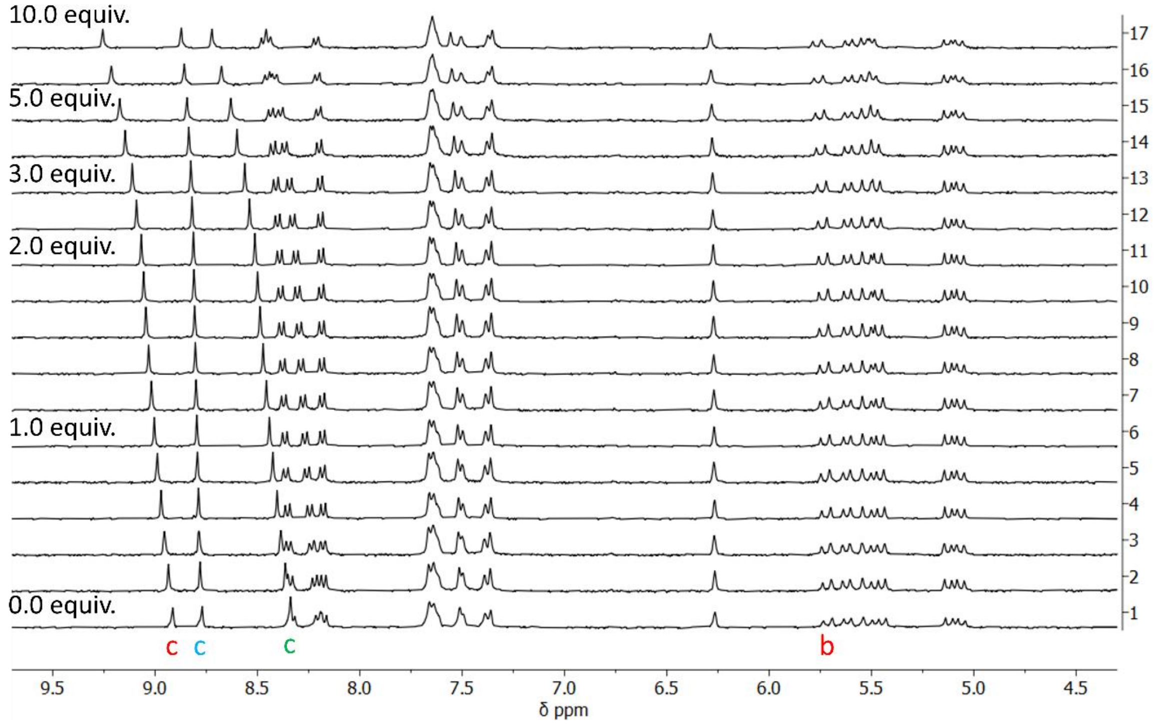

Figure S2.7 Partial ^{1}H NMR spectra of **M·(BF$_4$)$_8$** upon the addition of increasing equivalents of TBA·Br (0 to 10.0 equivalents) (298 K, 400 MHz, 1:1 [D$_3$]acetonitrile:CD$_3$OD).

2.6.2. Br^- binding to **M·$(BF_4)_8$** at 333 K

Considering literature studies[30] where the reported tetrahedra rearranged to more adequately encapsulate the various anions after heating, we heated **M·$(BF_4)_8$** in the presence of TBA·Br (10 equiv) initially at 50 °C for 12 hrs, and then at 70 °C for 8 hrs. After heating, spectra (collected at 298 K) showed no change in cage symmetry nor any further encapsulation of the anion, and only upon collecting the ^{1}H NMR at 333 K was a broad spectrum obtained, showing coalescence of all environments. We suggest that this is **M·$(BF_4)_8$** in an averaged conformation as similar systems have observed,[47] though it is interesting that both heat and an anionic guest is required to force the cage into this averaged state. Without the presence of Br^-, there is no significant change in isomer symmetry (Figure S1.30).

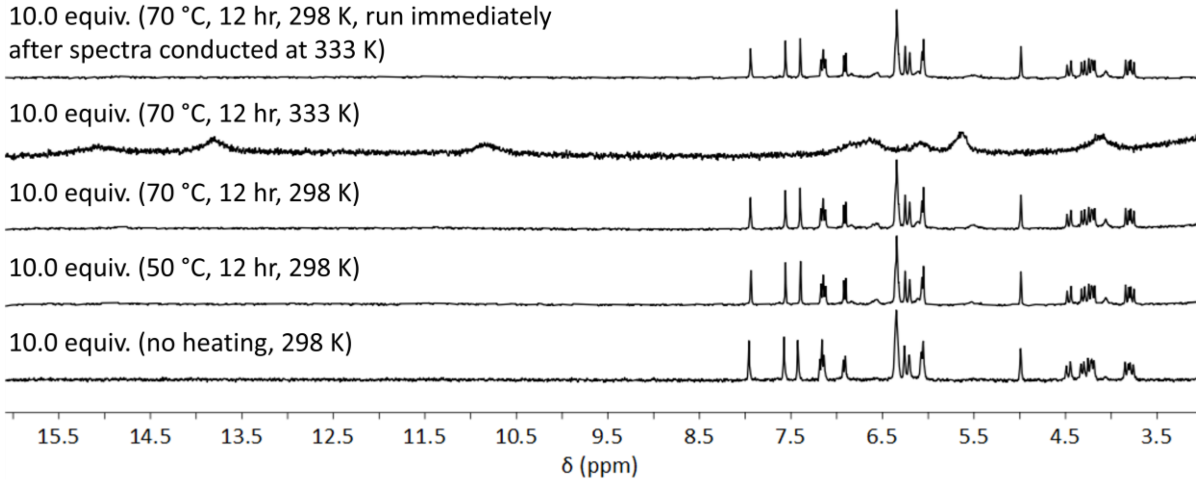

Figure S2.8 Partial ^{1}H NMR spectra of **M·$(BF_4)_8$** with TBA·Br (10.0 equivalents) with various heating conditions (298 K or 333 K, 400 MHz, 1:1 [D_3]acetonitrile:CD_3OD).

3. Crystallography

3.1. Structure of [S_4-[$Fe_4(L)_4$⊂NCMe]Na·$(BF_4)_6$·MeCN]$^{3+}$

Crystals of **[S_4-[$Fe_4(L)_4$⊂NCMe]Na·$(BF_4)_6$·MeCN]$^{3+}$** were obtained by vapour diffusion of diethyl ether into a solution of $Fe_4(L)_4](BF_4)_8$ in acetonitrile.

Diffraction data were collected on an Oxford Diffraction SuperNova instrument with a HyPix detector using Cu radiation at 150 K. Raw frame data (including data reduction, interframe scaling, unit cell refinement and absorption corrections) were processed using CrysAlis Pro.[114] The structure was solved using SHELXT[115] and refined using full-matrix least-squares on F^2 with SHELXL[116] within the Olex2 1.5 suite.[77] Data were weak at high angle owing to solvent lose issues and is ascribed to the large solvent/anion regions in the structure. Data were collected only to 1.18 Å based on diffraction limit estimates during screening in order to achieve usable lower angle data by collecting fewer frames in the still long 48 hr collection. This led to various restraints being introduced as well as a solvent mask, as implemented in the Olex2 package.

The initial solution allowed a quite well developed refinement that clearly established the features of the cage structure, along with some MeCN solvent molecules and many of the BF_4^- anions. However, portions of the structure geometry proved unacceptable and Olex2 FragmentDB tools[117,118] were implemented to give a stable refinement with acceptable geometry. The standard benzene fragment was introduced in the hub of the ligand and for the trischelate Fe^{II} complex fragment a well defined $Fe(L)_3^{2+}$ unit from a related pyridyltriazole complex was imported.[119]

Standard geometry and ADP restraints were used, with very limited geometry (DFIX) and ADP (SIMU) restraints being placed on some of the polyether chains to afford stable, acceptable refinement models. It was apparent that one of the well ordered polyether chains was associated with a nearby atom and models were developed based on H-bonded water and chelation of a metal to the O-atoms of the ether functional groups. Water was ruled out on the basis of geometry considerations (O···O and O···F^- distances regardless of the possible directionality of the H atoms) and the final refinement includes an adventitious Na^+ cation. No ADP restraints were added to the heavier atoms (Fe^{II}) due to the stronger contribution of those atoms to the diffraction pattern.

The solvent mask regions included some BF_4^- anions and, presumably, MeCN solvent molecules for all but the two well ordered MeCN molecules that were refined inside the cage and adjacent to the cavity. For the encapsulated MeCN, no restraints were required, while the MeCN adjacent to the cage required minimal ADP restraints (ISOR). Additionally, 6 of the 12 polyether chains of the cage were handled with the solvent mask, truncated beyond the aryl ether O atom in the cases were chain modelling could not be achieved. We were reluctant to extend models for the chains where evidence based restrained systems were not possible. We note that the connectivity and molecular architecture of the cage itself can be determined unambiguously and that these give important information about the tetrahedron despite the relatively low quality of the data.

Full crystallographic data in CIF format are provided as Supporting Information (CCDC Number: 2504227) and selected crystallographic data are provided in Table S1.

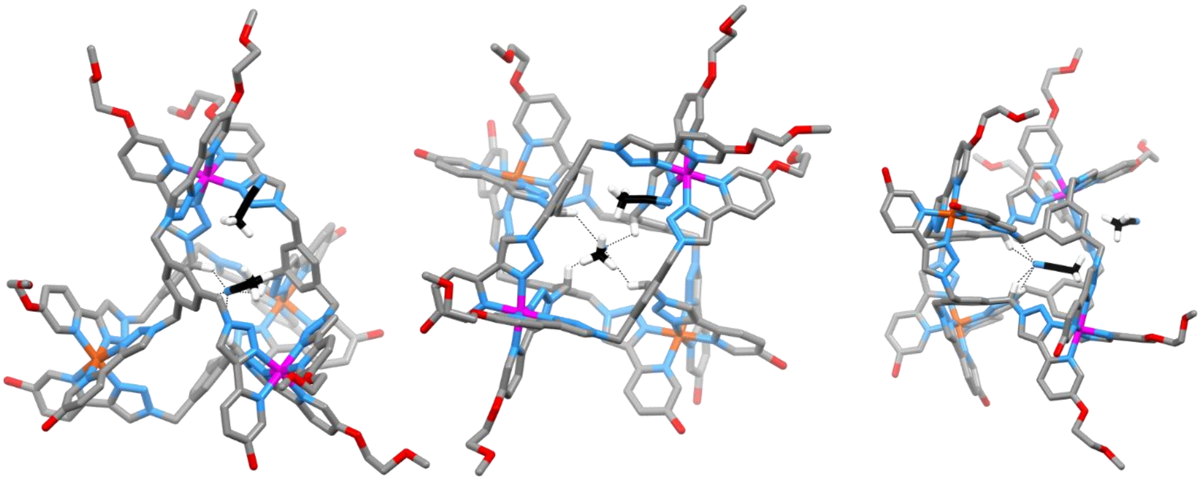

Figure S3.1 Different views of the single X-ray crystal structure of **[*S₄*-[Fe_4(L)$_4$⊂NCMe]Na·(BF_4)$_6$·MeCN]$^{3+}$**, dotted lines indicate a close contact shorter that the van der Waals' radii of H and N. Colours: cage carbon: grey, solvent carbon: black, nitrogen: blue, oxygen: red, Λ−Fe^{II}: orange, Δ−Fe^{II}: pink. Most hydrogen atoms, anions and cations are omitted for clarity. Solvent mask was used in the refinement, implemented within the Olex2 package.[77]

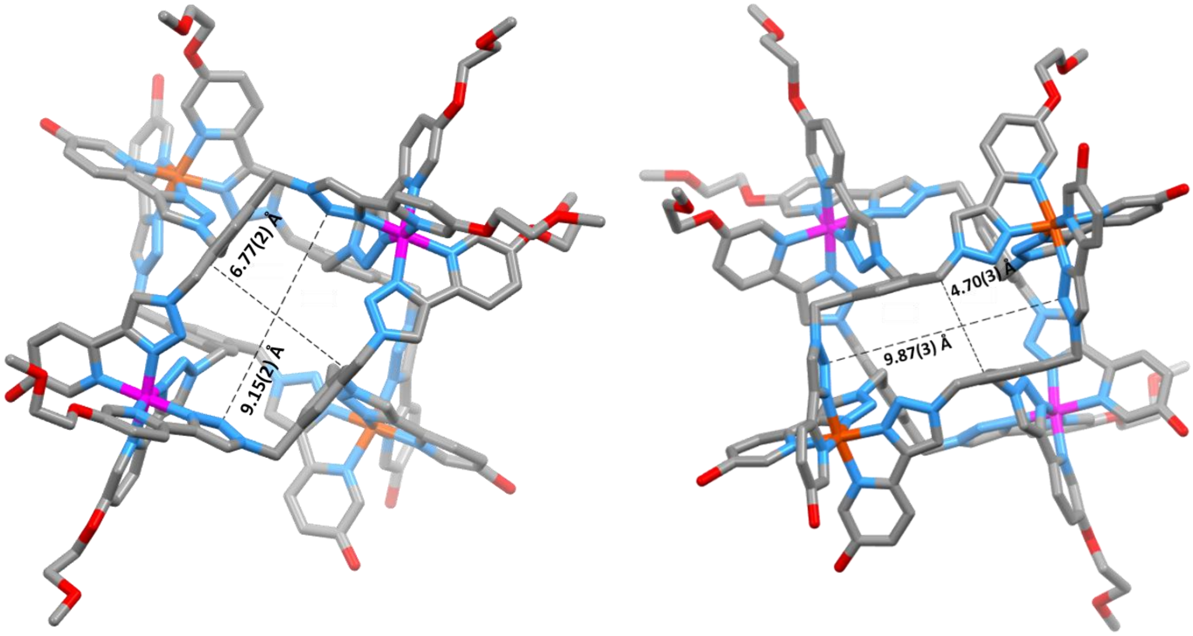

Figure S3.2 Cavity distances within single X-ray crystal structure of **[*S₄*-[Fe_4(L)$_4$⊂NCMe]Na·(BF_4)$_6$·MeCN]$^{3+}$** (left: enlarged cavity; right: collapsed cavity). Colours: carbon: grey, nitrogen: blue, oxygen: red, Λ−Fe^{II}: orange, Δ−Fe^{II}: pink. Hydrogen atoms, solvent, anions and cations are omitted for clarity. Solvent mask was used in the refinement, implemented within the Olex2 package.[77]

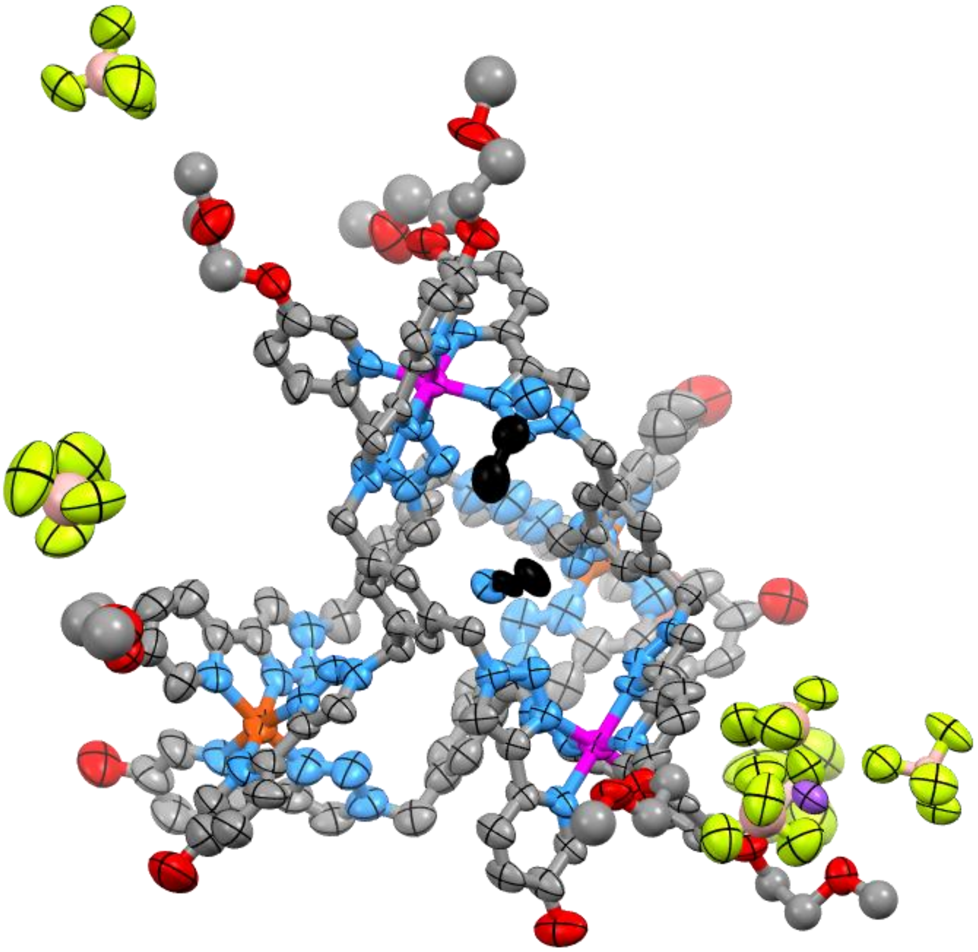

Figure S3.3 Thermal ellipsoid plot showing the asymmetric unit of **[*S₄*-[Fe₄(L)₄⊂NCMe]Na·(BF₄)₆·MeCN]³⁺**. Ellipsoids shown at 50% probability. Colours: cage carbon: grey, solvent carbon: black, boron: pale pink, nitrogen: blue, oxygen: red, fluorine: yellow, sodium: purple, Λ−Fe^{II}: orange, Δ−Fe^{II}: pink. Hydrogen atoms are omitted for clarity. Solvent mask was used in the refinement, implemented within the Olex2 package.[77]

Table S1. Selected crystallographic data.

**Compound**	**[S_4-[$Fe_4(L)_4$⊂NCMe]Na·$(BF_4)_6$·MeCN]$^{3+}$**
Radiation type	Cu (λ = 1.54184 Å)
Temperature (K)	150
Formula	$C_{142}H_{132}B_6F_{24}Fe_4N_{50}NaO_{18}$·*polyether chain ends ·anions ·solvents*[a]
Formula weight	3594.22
a (Å)	18.2937(6)
b (Å)	19.0684(6)
c (Å)	65.4409(17)
α (°)	90
β (°)	96.165(3)
γ (°)	90
Unit cell volume (Å^3)	22695.9(12)
Crystal system	monoclinic
Space group	$P\,1\,2_1/n\,1$
Z	4
Reflections (all)	53143
Reflections (unique)	13885
R_{int}	0.054
R_1 [$I > 2\sigma(I)$]	0.127
wR_2 (all data)	0.398
CCDC number	2504227

[a] Solvent mask used.[77]

4. Symmetry elements

As discussed, in acetonitrile the major tetrahedral diastereomer isolated has *meso* S_4 symmetry (ΔΔΛΛ) (Figure S4.1a), causing the ligands to have a reduced symmetry (C_1) with (*P*)/(*M*) helicity (Figure S4.1b). The ligands with (*P*) helicity link the Λ-Fe^{II} metal centers (Figure S4.1bi), and the ligands with (*M*) helicity link the Δ-Fe^{II} metal centers (Figure S4.1bii).

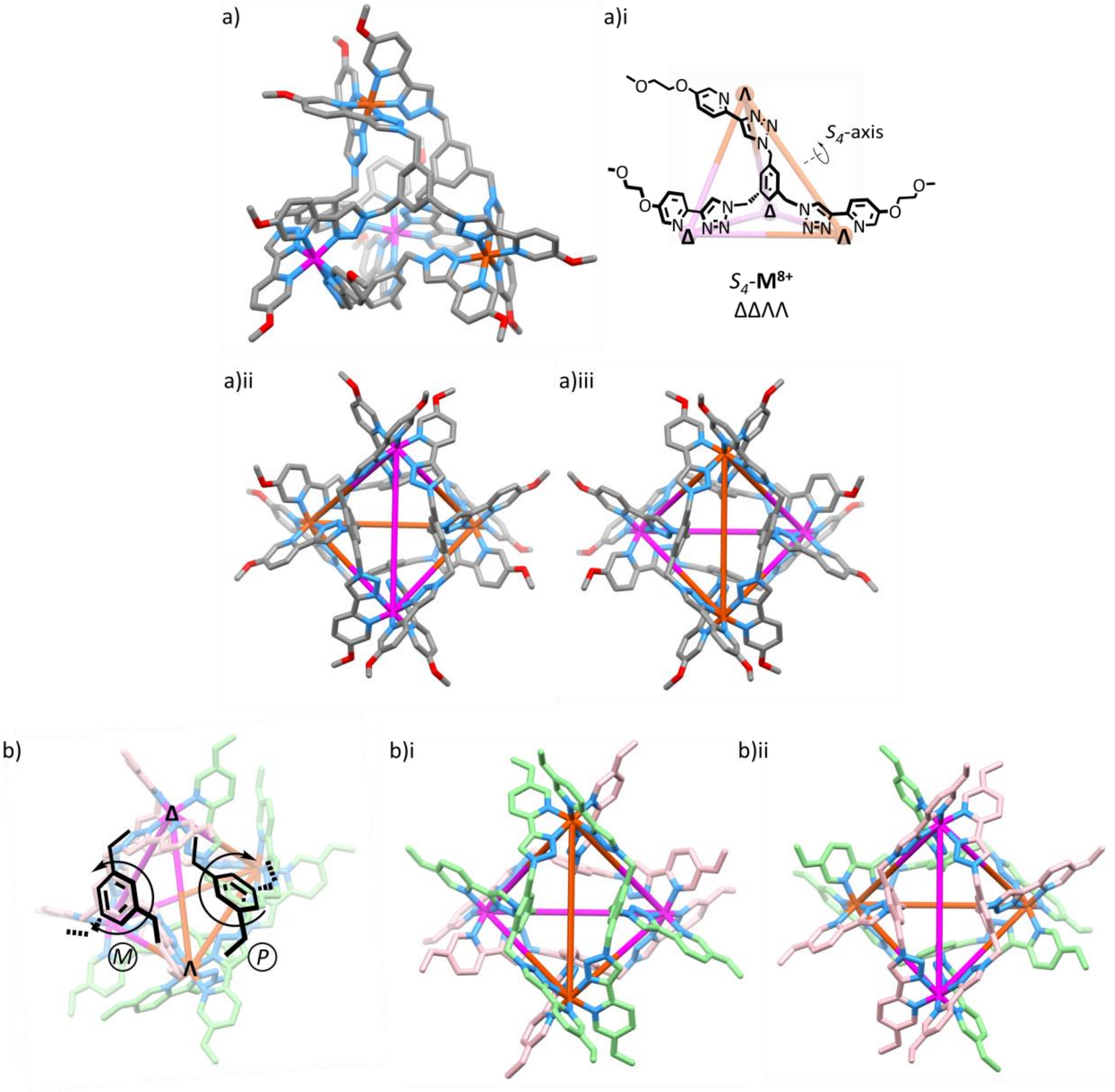

Figure S4.1 Different views of r^{2}SCAN-3c/CPCM(MeCN)-optimised $\boldsymbol{S_4}$–**M**$^{8+}$ cage, showing relevant symmetry operations that result in the reported achiral cage: a) $\boldsymbol{S_4}$–**M**$^{8+}$; a)i) structure of $\boldsymbol{S_4}$–**M**$^{8+}$ showing all identified symmetry elements; a)ii) structure of $\boldsymbol{S_4}$–**M**$^{8+}$ viewed down ΔΔ (Fe^{II}: pink) edges; a)iii) structure of $\boldsymbol{S_4}$–**M**$^{8+}$ viewed down ΛΛ (Fe^{II}: orange) edges; b) structure of $\boldsymbol{S_4}$–**M**$^{8+}$ showing (*P*)/(*M*) helicity down ΔΛ edge; b)i) structure of $\boldsymbol{S_4}$–**M**$^{8+}$ showing (*M*) helical ligands (light pink) along ΔΔ (Fe^{II}: pink) edges; b)ii) structure of $\boldsymbol{S_4}$–**M**$^{8+}$ showing (*P*) helical ligands (light green) along ΛΛ (Fe^{II}: orange) edges. Colours: carbon: grey, nitrogen: blue, oxygen: red, Δ-Fe^{II}: pink; Λ-Fe^{II}: orange. Hydrogen atoms are omitted for clarity.

Upon addition of nitromethane a small fraction of the homochiral *T* symmetry tetrahedron forms (ΔΔΔΔ or ΛΛΛΛ; Figure S4.1a), which given the flexibility of the ligands could exist in equilibrium as the homochiral D_2 conformer (ΔΔΔΔ or ΛΛΛΛ; Figure S4.1b), with the ligands having (*P*) helicity.

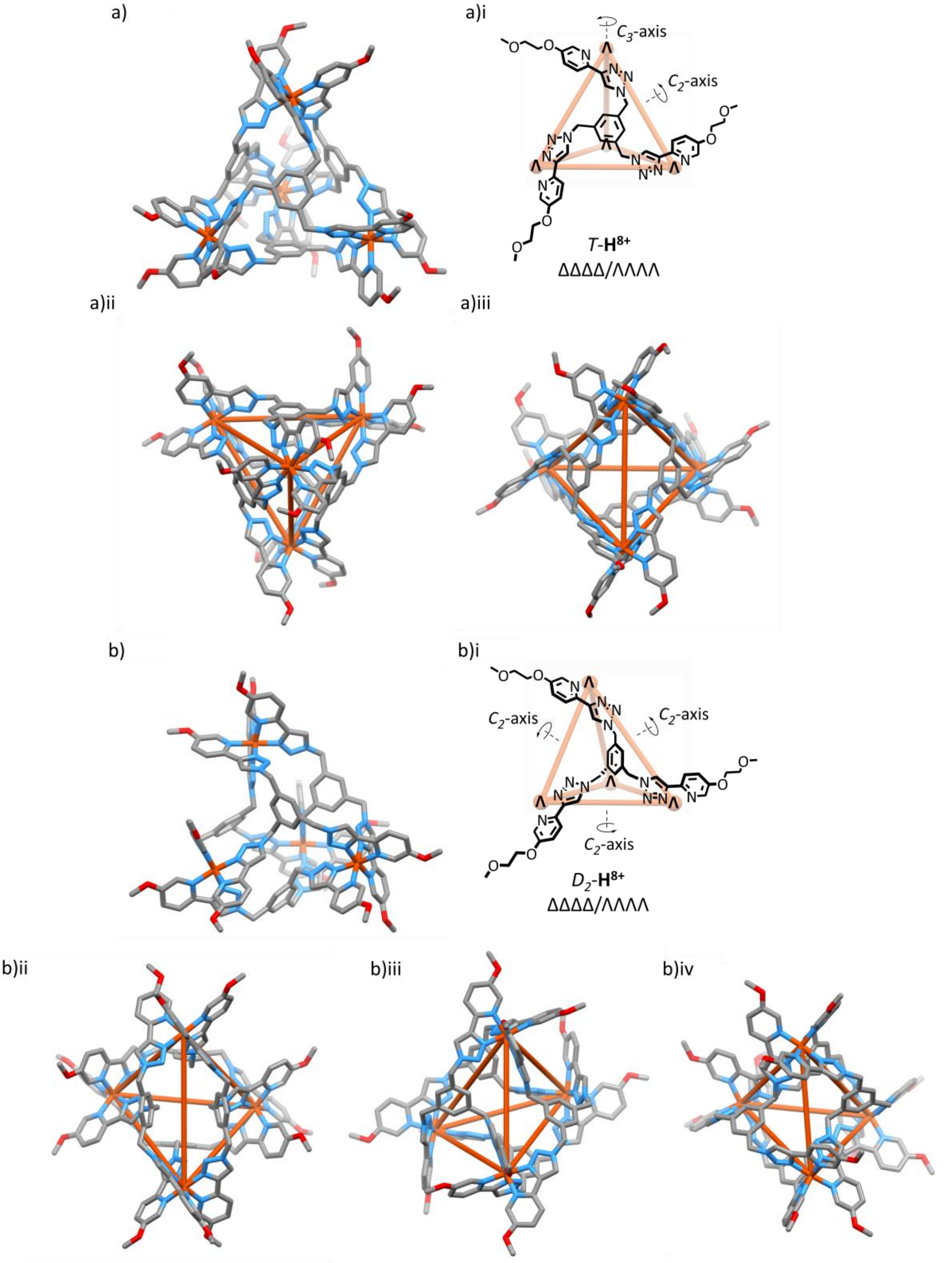

Figure S4.2 Different views of r^{2}SCAN-3c/CPCM(MeCN)-optimised reported tetrahedra, showing relevant symmetry operations that result in the homochiral cages: a) ***T*–H^{8+}**; a)i) structure of ***T*–H^{8+}** showing all identified symmetry elements; a)ii) structure of ***T*–H^{8+}** showing C_3 symmetry; a)iii) structure of ***T*–H^{8+}** showing C_2 symmetry; b) ***D*$_2$–H^{8+}**; b)i) structure of ***D*$_2$–H^{8+}** showing all identified symmetry elements; b)ii)-d)iv) structure of ***D*$_2$–H^{8+}** showing C_2 symmetry axis. Colours: carbon: grey, nitrogen: blue, oxygen: red; Fe^{II}: orange. Hydrogen atoms are omitted for clarity.

5. Calculations

5.1. General

5.1.1. Initial structure optimisation

The initial idealised representation of the cages without counter anions (**M**$^{8+}$ or ***D*$_2$–H**$^{8+}$ or ***T*–H**$^{8+}$) and solvents (MeCN and $MeNO_2$) were constructed using Spartan.[120] These structures were then optimised using the quantum mechanical extended tight binding method GFN1-xTB 6.7.1,[80] as implemented in the *xtb* code. Optimisations were performed using GFN 1, an "extreme" optimization [extreme: Econv (energy convergence) = 5×10^{-8} Eh; Gconv (gradient convergence) = 5×10^{-5} Eh·α^{-1}; accuracy (for integral cutoffs and SCF criteria) = 0.01] and the solvent acetonitrile was represented with an implicit solvation model (ALPB, as implemented in the xTB code).[121] Lebedev grid level used was "normal" with 230 grid points (as implemented in the xTB code).[122] These optimised structures were then used as the input for the molecular dynamic simulations and solvent docking calculations (see below).

5.1.2. Molecular dynamic simulations

Molecular dynamic (MD) simulations were employed in order to probe the dynamic equilibrium between the theorized ***D*$_2$–H**$^{8+}$ or ***T*–H**$^{8+}$ conformations. All GFN1-xTB MD calculations were performed with initial "loose" optimization [loose: *E*conv (energy convergence) = 5×10^{-5} *E*h; *G*conv (gradient convergence) = 4×10^{-3} *E*h·α^{-1}; accuracy (for integral cutoffs and SCF criteria) = 2.00] and the solvent acetonitrile was represented with an implicit solvation model (ALPB, as implemented in the xTB code).[121] Lebedev grid level used was "normal" with 230 grid points (as implemented in the xTB code).[122] The simulations were conducted in the NVT ensemble with the system temperature being maintained at 1000 K. All bonds were constrained using the SHAKE algorithm.[123] Each simulation was run for 800 ps, with trajectory output every 500 fs, with a propagation time step of 4 fs.

Considering that ***D*$_2$–H**$^{8+}$ has a more condensed structure compared to ***T*–H**$^{8+}$ (Table S2), we analysed the change in Fe^{II}–Fe^{II} distances (Å) along with the relative change in energy (ΔE) to approximate when the cage moves from the ***T*–H**$^{8+}$ conformer to the ***D*$_2$–H**$^{8+}$ conformer (and vice versa). For this analysis, the first 90 frames were removed from the data set so as to remove the initial loose optimization and ensure that the system was at equilibrium.

5.1.3. Solvent docking calculations

Solvent docking calculations (automated Interaction Site Screening (aISS)[78]) were performed with **M**$^{8+}$ and ***D*$_2$–H**$^{8+}$ using the xTB program version 6.7.1,[124] with the interaction site screening and genetic optimization done with the intermolecular force field xTB-IFF,[79] followed by geometry optimizations with GFN1-xTB.[80] An additional pocket search of fragment A was employed to search for adequate cavities for the explicit solvent guest, while the general implicit solvent acetonitrile was represented with an implicit solvation model (ALPB, as implemented in the xTB code).[121]

To generate structures with either one or two explicit solvent guests (**M**$^{8+}$**⊂solvent**$_x$ or ***D*$_2$–H**$^{8+}$**⊂solvent**$_x$), initially the docking between cage Fragment A (**M**$^{8+}$ or ***D*$_2$–H**$^{8+}$) and solvent Fragment B (MeCN and $MeNO_2$) was calculated using the aISS//GFN1-xTB algorithm, generating the most favourable complexes of **M**$^{8+}$**⊂solvent** or ***D*$_2$–H**$^{8+}$**⊂solvent**. To calculate the complex with a second explicit solvent guest, the most favourable complex (**M**$^{8+}$**⊂solvent** or ***D*$_2$–H**$^{8+}$**⊂solvent**) was used as the new cage Fragment A, while a further solvent guest (Fragment B, MeCN and $MeNO_2$

respectively) was included in the calculation to generate favourable complexes of **M^{8+}⊂solvent$_2$** or ***D_2*–H^{8+}⊂solvent$_2$**.

5.2. Dynamic equilibrium of *D_2*–H^{8+} and *T*–H^{8+}

To determine whether ***T*–H^{8+}** could collapse to a *D_2* isomer we analysed molecular dynamic simulations, starting from both the proposed ***T*–H^{8+}** structure and the ***D_2*–H^{8+}** structure (Figure S4.1). Interestingly, despite the energetic differences between the cages, there appears to be no dominant isomer, the simulations indicating that ***T*–H^{8+}** can collapse to the *D_2* isomer, but that ***D_2*–H^{8+}** can also enlarge to the higher symmetry ***T*–H^{8+}**. These cages had distinct average intramolecular distances between the Fe^{II} metal ion vertices (Table S2), and as such we used this as an indicator to approximate when the assembly is more similar to the ***T*–H^{8+}** isomer or the ***D_2*–H^{8+}** isomer (Figure S4.2 bottom). These approximations also coincide with small changes in the energetic landscape (Figures S4.2 top), though this is much less apparent. It should be noted that these systems are highly fluctional, and the progression from one isomer to the other is a dynamic process. As such, we have identified moments in the simulation where the *majority* of Fe^{II}–Fe^{II} distances more closely resemble the ***T*–H^{8+}** isomer (red boxes, Figure S2.4 bottom) and the ***D_2*–H^{8+}** isomer (blue boxes, Figure S4.2 bottom).

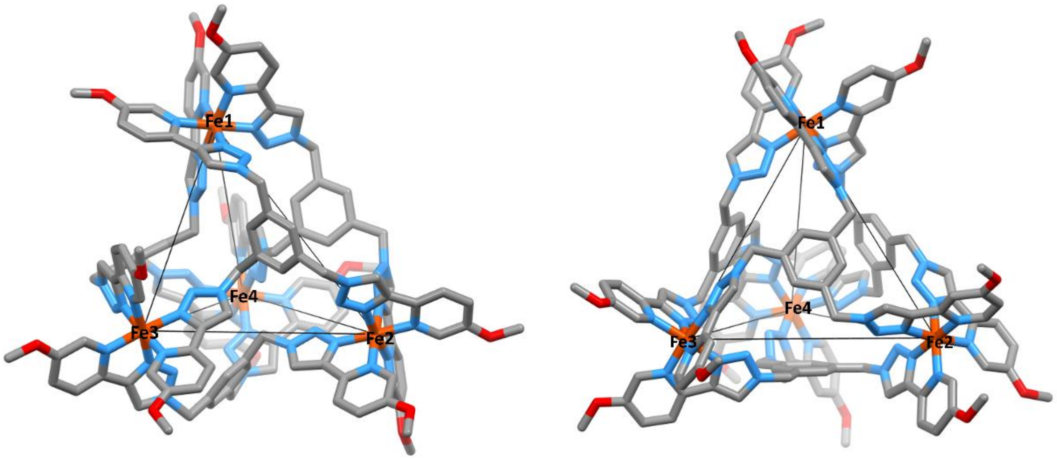

Figure S5.1 xtb-GFN1 optimised initial structures of ***D_2*–H^{8+}** (left) and ***T*–H^{8+}** (right). Labelling corresponds to labels in Table S2 and time trace graphs (Figure S4.2). Colours: carbon: grey, nitrogen: blue, oxygen: red, Fe^{II}: orange. Hydrogen atoms are omitted for clarity.

Table S2. Comparison of Fe^{II}–Fe^{II} distances (Å) between xtb-GFN1 optimised structures ***D_2*–H^{8+}** and ***T*–H^{8+}**.

**Fe^{II}–Fe^{II} distances (Å)**	***D_2*–H^{8+}**	***T*–H^{8+}**
**Fe1–Fe2**	11.974	11.730
**Fe1–Fe3**	10.206	11.709
**Fe1–Fe4**	10.795	11.729
**Fe2–Fe4**	10.200	11.727
**Fe2–Fe3**	10.771	11.724
**Fe3–Fe4**	11.140	11.711
**Average Fe–Fe**	10.848	11.722

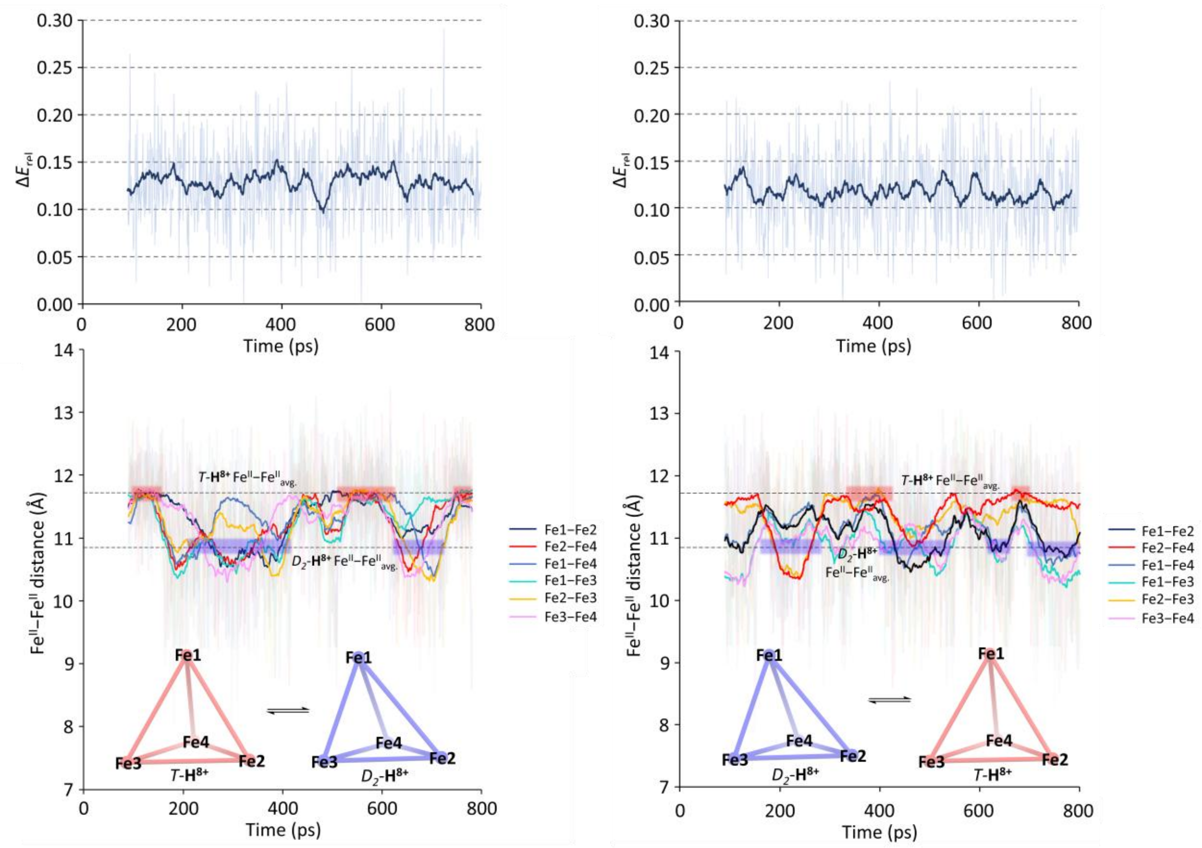

Figure S5.2 MD simulation time traces of $\mathbf{H^{8+}}$ starting from initial geometry ***T*–H**$^{8+}$ (left) and ***D*$_2$–H**$^{8+}$ (right). *Top graph:* relative change in energy (ΔE_{rel}), with pale data showing full energy change, while bold lines show rolling average (intervals of 25 ps). *Bottom graph:* change in intramolecular Fe^{II}–Fe^{II} distance (Å). Pale lines show full data, bold trend lines show rolling average for each Fe^{II}–Fe^{II} distance (intervals of 50 ps). Dotted lines at 10.8 Å and 11.7 Å represent the average Fe^{II}–Fe^{II} distance of ***D*$_2$-H**$^{8+}$ and ***T*-H**$^{8+}$ calculated from initial starting geometries. Transparent red and blue boxes along these dotted lines indicate when $\mathbf{H^{8+}}$ is predominantly a ***T*-H**$^{8+}$ or ***D*$_2$-H**$^{8+}$ isomer, respectively. Labelling corresponds to labels in Figure S4.1 and Table S2.

5.3. Docking studies for M^{8+}⊂solvent$_x$ and *D*$_2$–H^{8+}⊂solvent$_x$

The following primarily analyses assemblies of **M^{8+}⊂solvent$_x$** and ***D*$_2$–H^{8+}⊂solvent$_x$** which could conceivably exist within solution. As these structures are further optimised at higher levels of theory (DFT, Section 4.4), the respective interaction energies for each structure was not thoroughly interrogated. As such, the following section discusses the structural aspects of the complexes and whether one or two solvent guests can be encapsulated within the cage with no significant consideration of the obtained interaction energies.

5.3.1. Docking between M^{8+} and $MeCN_x$

5.3.1.1. Docking between $M^{8+}\subset$NCMe

The most favourable geometry between **M^{8+}** and MeCN (Figure S4.3) showed the solvent guest MeCN within the cavity of **M^{8+}**, with the solvent guest appearing to receive six C–H_b···NCMe hydrogen bonds (H_b···NCMe distances: 2.35 – 2.76 Å, 82 – 96% of the sum of van der Waals radii[76]).

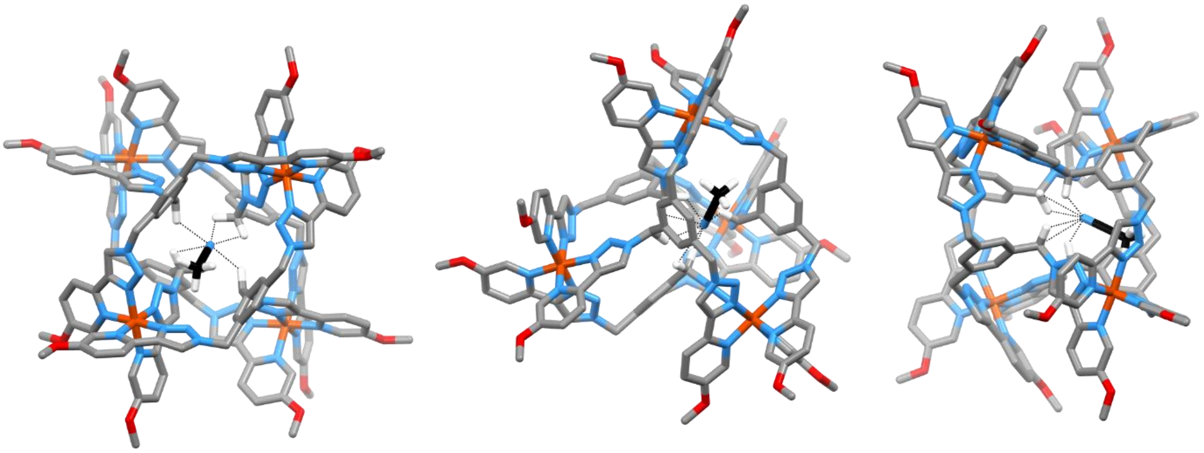

Figure S5.3 Different views of the most favourable structure of **$M^{8+}\subset$NCMe** found with the aISS//GFN1-FF algorithm, dotted lines indicate a close contact shorter that the van der Waals' radii of H and N. Colours: cage carbon: grey, solvent carbon: black, nitrogen: blue, oxygen: red, Λ/Δ –Fe^{II}: orange. Most hydrogen atoms are omitted for clarity.

5.3.1.2. Docking between [$M^{8+}\subset$NCMe]MeCN

The most favourable geometry between **$M^{8+}\subset$NCMe** and MeCN (Figure S4.4) showed the second solvent guest MeCN on the exterior of **$M^{8+}\subset$NCMe**, with no obvious interactions occurring. There appears to be no significant change in the interior solvent bonding mode, with the encapsulated MeCN still receiving six C–H_b···NCMe hydrogen bonds (H_b···NCMe distances: 2.37 – 2.77 Å, 83 – 97% of the sum of van der Waals radii[76]).

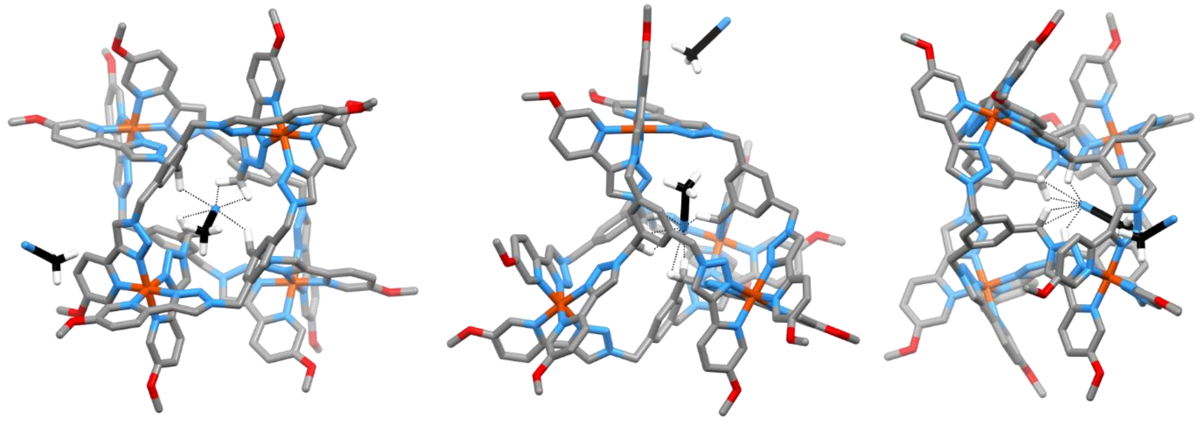

Figure S5.4 Different views of the most favourable structure of **[$M^{8+}\subset$NCMe]MeCN** found with the aISS//GFN1-FF algorithm. Colours: cage carbon: grey, solvent carbon: black, nitrogen: blue, oxygen: red, Λ/Δ –Fe^{II}: orange. Most hydrogen atoms are omitted for clarity.

5.3.2. Docking between M^{8+} and $(MeNO_2)_x$

5.3.2.1. Docking between $M^{8+}\subset O_2NMe$

The most favourable geometry between $\mathbf{M^{8+}}$ and $MeNO_2$ (Figure S4.5) showed the solvent guest $MeNO_2$ within the cavity of $\mathbf{M^{8+}}$, with the solvent guest appearing to receive two C–H_b···O_2NMe hydrogen bonds (H_b··· O_2NMe distances: 2.39 – 2.43 Å, 89 – 90% of the sum of van der Waals radii[76]).

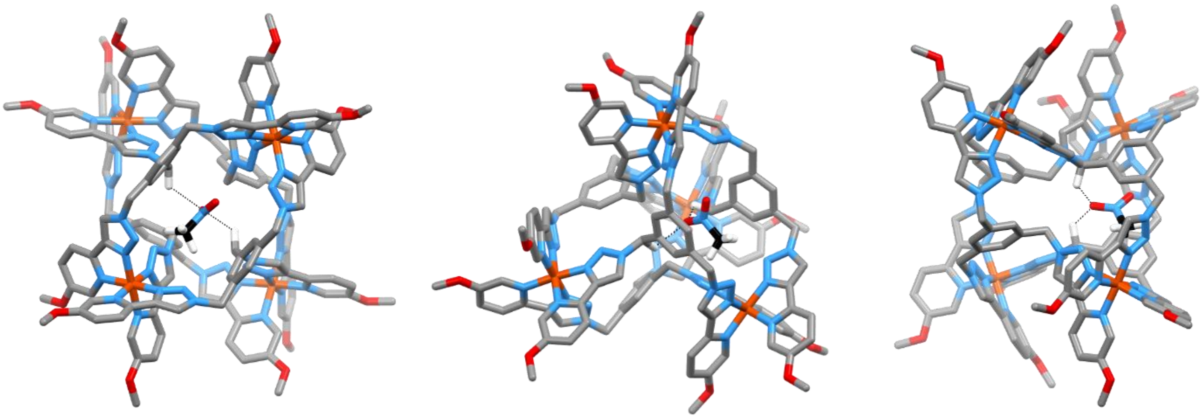

Figure S5.5 Different views of the most favourable structure of $\mathbf{M^{8+}\subset O_2NMe}$ found with the aISS//GFN1-FF algorithm, dotted lines indicate a close contact shorter that the van der Waals' radii of H and O. Colours: cage carbon: grey, solvent carbon: black, nitrogen: blue, oxygen: red, Λ/Δ −Fe^{II}: orange. Most hydrogen atoms are omitted for clarity.

5.3.2.2. Docking between $[M^{8+}\subset O_2NMe]MeNO_2$

The most favourable geometry between $\mathbf{M^{8+}\subset O_2NMe}$ and $MeNO_2$ (Figure S4.6) showed the second solvent guest $MeNO_2$ on the exterior of $\mathbf{M^{8+}\subset O_2NMe}$, with the second solvent appearing to receive an exterior C–H_c···O_2NMe hydrogen bond (H_c···O_2NMe distance: 2.44 Å, 90% of the sum of van der Waals radii[76]). The initial solvent guest continues to receive two C–H_b···O_2NMe hydrogen bonds (H_b··· O_2NMe distances: 2.40 – 2.47 Å, 89 – 91% of the sum of van der Waals radii[76]), consistent with $\mathbf{M^{8+}\subset O_2NMe}$.

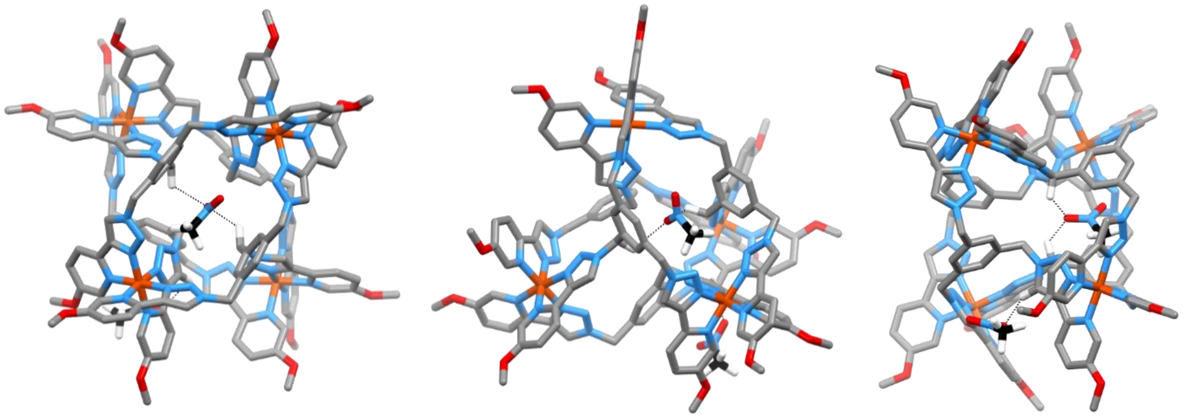

Figure S5.6 Different views of the most favourable structure of $\mathbf{[M^{8+}\subset O_2NMe]MeNO_2}$ found with the aISS//GFN1-FF algorithm, dotted lines indicate a close contact shorter that the van der Waals' radii of H and O. Colours: cage carbon: grey, solvent carbon: black, nitrogen: blue, oxygen: red, Λ/Δ −Fe^{II}: orange. Most hydrogen atoms are omitted for clarity.

5.3.3. Docking between D_2–H^{8+} and MeCN$_x$

5.3.3.1. Docking between D_2–H^{8+}⊂NCMe

The most favourable geometry between ***D***D_2**–H^{8+}** and MeCN (Figure S4.7) showed the solvent guest MeCN within the cavity of **D_2–H^{8+}**, with the solvent guest appearing to receive seven C–H$_b$···NCMe hydrogen bonds (H$_b$···NCMe distances: 2.54 – 2.79 Å, 89 – 97% of the sum of van der Waals radii[76]).

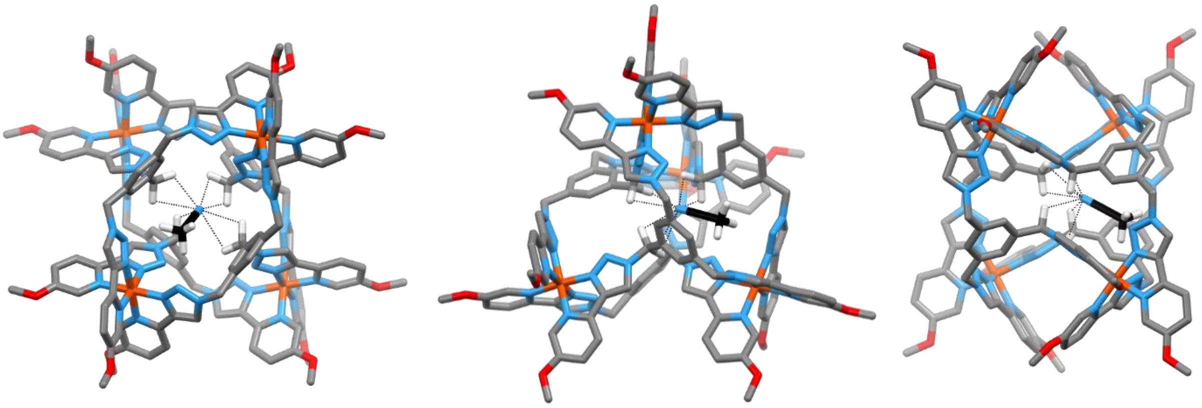

Figure S5.7 Different views of the most favourable structure of **D_2–H^{8+}⊂NCMe** found with the aISS//GFN1-FF algorithm, dotted lines indicate a close contact shorter that the van der Waals' radii of H and N. Colours: cage carbon: grey, solvent carbon: black, nitrogen: blue, oxygen: red, Λ/Δ –Fe^{II}: orange. Most hydrogen atoms are omitted for clarity.

5.3.3.2. Docking between D_2–H^{8+}⊂NCMe$_2$

The most favourable geometry between **D_2–H^{8+}⊂NCMe** and MeCN (Figure S4.8) showed the second solvent guest MeCN within the second cavity of **D_2–H^{8+}⊂NCMe**. In order to accommodate the second solvent, the cavity enlarges slightly (increase of ~6%, compared to **D_2–H^{8+}⊂NCMe**), leading to both solvent guests appearing to only receive two C–H$_b$···NCMe hydrogen bonds (H$_b$···NCMe distances: 2.41 – 2.68 Å, 84 – 94% of the sum of van der Waals radii[76]).

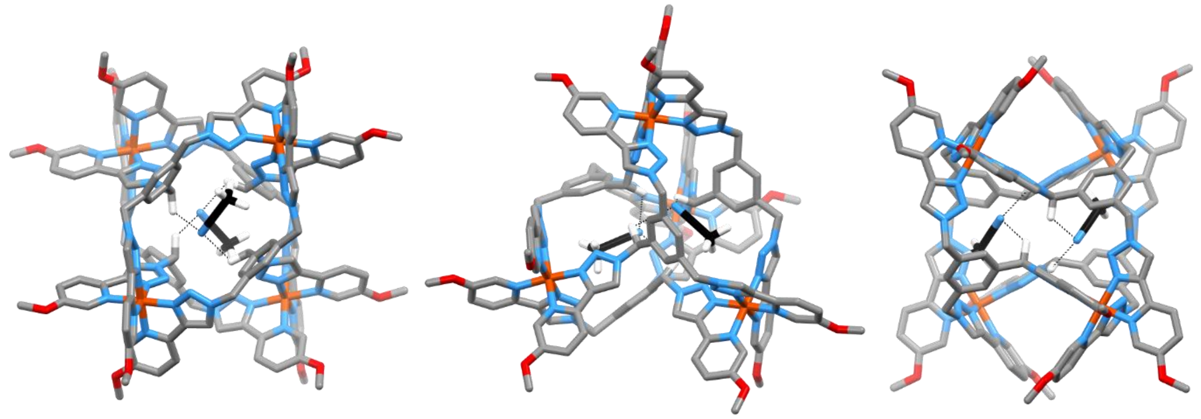

Figure S5.8 Different views of the most favourable structure of **D_2–H^{8+}⊂NCMe$_2$** found with the aISS//GFN1-FF algorithm, dotted lines indicate a close contact shorter that the van der Waals' radii of H and N. Colours: cage carbon: grey, solvent carbon: black, nitrogen: blue, oxygen: red, Λ/Δ –Fe^{II}: orange. Most hydrogen atoms are omitted for clarity.

5.3.4. Docking between D_2–H^{8+} and $(MeNO_2)_x$

5.3.4.1. Docking between D_2–H^{8+}⊂O_2NMe

The most favourable geometry between D_2–H^{8+} and $MeNO_2$ (Figure S4.9) showed the solvent guest $MeNO_2$ within the cavity of D_2–H^{8+}, with the solvent guest appearing to be receive three C–H_b···O_2NMe hydrogen bonds (H_b···O_2NMe distances: 2.53 – 2.70 Å, 94 – 100% of the sum of van der Waals radii[76]).

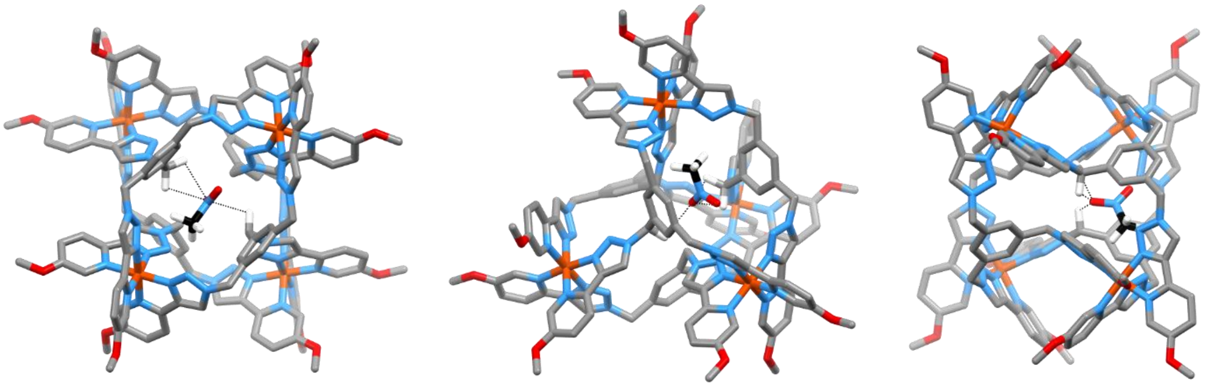

Figure S5.9 Different views of the most favourable structure of **D_2–H^{8+}⊂O_2NMe** found with the aISS//GFN1-FF algorithm, dotted lines indicate a close contact shorter that the van der Waals' radii of H and O. Colours: cage carbon: grey, solvent carbon: black, nitrogen: blue, oxygen: red, Λ/Δ –Fe^{II}: orange. Most hydrogen atoms are omitted for clarity.

5.3.4.2. Docking geometries between [D_2–H^{8+}⊂O_2NMe]$MeNO_2$

The most favourable geometry between **D_2–H^{8+}⊂O_2NMe** and $MeNO_2$ (Figure S4.10) showed the second solvent guest $MeNO_2$ on the exterior of **D_2–H^{8+}⊂O_2NMe**, with the second solvent appearing to be hydrogen bonding to the encapsulated solvent (CH_3···O_2NMe distances: 2.28 – 2.61 Å, 84 – 97% of the sum of van der Waals radii[76]). In contrast to **D_2–H^{8+}⊂O_2NMe**, the encapsulated solvent guest is only weakly bound to the interior of the cage with one C–H_b···O_2NMe hydrogen bond (H_b···O_2NMe distance: 2.54 Å, 94% of the sum of van der Waals radii[76]).

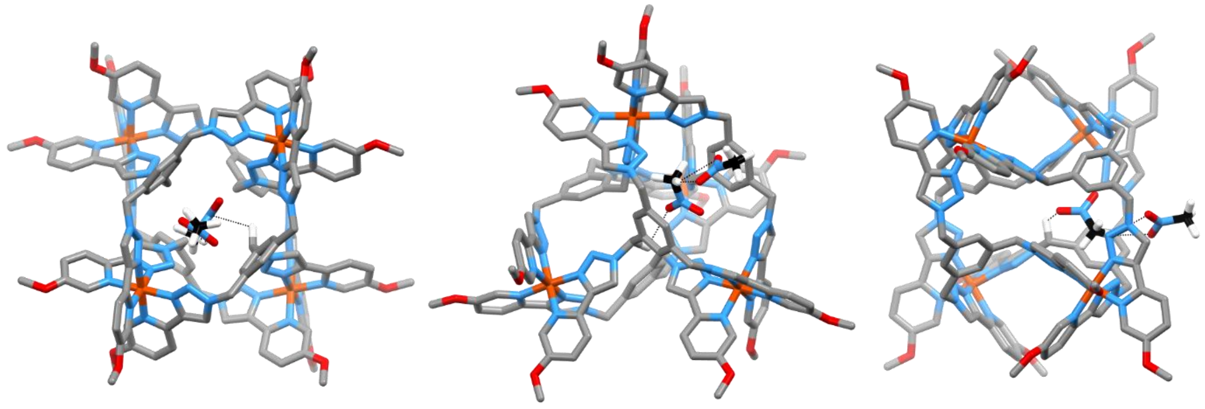

Figure S5.10 Different views of the most favourable structure of **[D_2–H^{8+}⊂O_2NMe]$MeNO_2$** found with the aISS//GFN1-FF algorithm, dotted lines indicate a close contact shorter that the van der Waals' radii of H and O. Colours: cage carbon: grey, solvent carbon: black, nitrogen: blue, oxygen: red, Λ/Δ –Fe^{II}: orange. Most hydrogen atoms are omitted for clarity.

5.4. Tetrahedra energies and solvent interaction energy

5.4.1. General

Density functional theory (DFT) calculations were performed using the ORCA program version 6.0.1.[125] Structures were fully optimized using the r^2SCAN-3c functional.[81–82, 126] Structures were

optimised with tight convergence criteria on both the geometry and self-consistent field (SCF) cycles. The conductor-like polarizable continuum model (CPCM) was used to treat the structures in an acetonitrile (MeCN) environment.

Starting structures were taken from aISS//GFN1-xTB algorithm outputted geometries (see Section 4.3). As it was apparent that only one solvent guest could be accommodated in $\mathbf{M^{8+}}$, only the geometries of $\mathbf{M^{8+}{\subset}NCMe}$, $\mathbf{M^{8+}{\subset}O_2NMe}$, $\boldsymbol{D_2}$**–**$\mathbf{H^{8+}{\subset}NCMe}$ and $\boldsymbol{D_2}$**–**$\mathbf{H^{8+}{\subset}O_2NMe}$ were analysed at higher levels of theory (DFT). To determine interaction energies and compare overall isomer favourability, the single point energies of the separate components of each complex (ie $\mathbf{M^{8+}}$, $\boldsymbol{D_2}$**–**$\mathbf{H^{8+}}$, $\boldsymbol{T}$**–**$\mathbf{H^{8+}}$, MeCN and $MeNO_2$) were also calculated using the method as detailed above. Interaction energies between the host and the solvent guest were calculated by the equation:

$$E_{int} = (E_{\mathrm{cage8+\subset solvent}}) - (E_{\mathrm{cage8+}}) - (E_{\mathrm{solvent}})$$

Table S3 Calculated E_{tot} (E_h) and ΔE_{tot} (kJ/mol) of optimised tetrahedra. ΔE_{tot} calculated against $\mathbf{M^{8+}}$ and $\mathbf{M^{8+}{\subset}solvent}$ in all cases, respectively.

Tetrahedra	E_{tot} (E_h)	ΔE_{tot} (kJ/mol)
$\mathbf{M^{8+}}$	−13680.39	0
$\boldsymbol{D_2}$**–**$\mathbf{H^{8+}}$	−13680.37	39.69
$\boldsymbol{T}$**–**$\mathbf{H^{8+}}$	−13680.41	−48.99
$\mathbf{M^{8+}{\subset}NCMe}$	−13813.05	0
$\boldsymbol{D_2}$**–**$\mathbf{H^{8+}{\subset}NCMe}$	−13813.04	46.32
$\mathbf{M^{8+}{\subset}O_2NMe}$	−13925.37	0
$\boldsymbol{D_2}$**–**$\mathbf{H^{8+}{\subset}O_2NMe}$	−13925.35	55.77
**MeCN**	−132.65	–
$\mathbf{MeNO_2}$	−244.96	–

5.4.2. Structural comparison between $M^{8+}\subset NCMe$ and crystal structure [*S₄*-[Fe₄(L)₄⊂NCMe]Na·(BF₄)₆·MeCN]³⁺

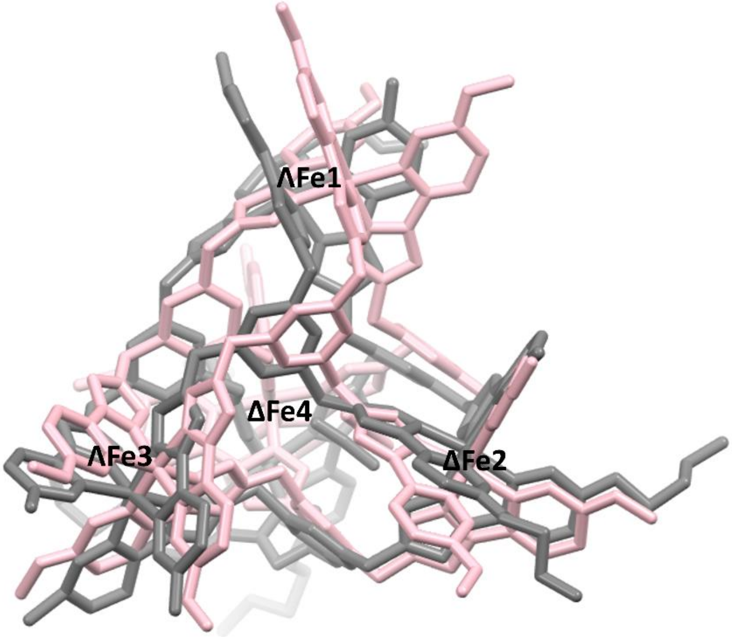

Figure S5.11 Overlayed structures of r^{2}SCAN-3c/CPCM(MeCN)-optimised **$M^{8+}\subset NCMe$** (pink) and crystal structure **[*S_4*-$[Fe_4(L)_4\subset NCMe]Na\cdot(BF_4)_6\cdot MeCN]^{3+}$** (grey). Hydrogen atoms, non-encapsulated solvent molecules, anions and cations are omitted for clarity.

Table S4 Comparison of Fe^{II}–Fe^{II} distances (Å) between optimised structure **$M^{8+}\subset NCMe$** and crystal structure **[*S_4*-$[Fe_4(L)_4\subset NCMe]Na\cdot(BF_4)_6\cdot MeCN]^{3+}$**

Fe^{II}–Fe^{II} distances (Å)	$M^{8+}\subset NCMe$	[*S_4*-$[Fe_4(L)_4\subset NCMe]Na\cdot(BF_4)_6\cdot MeCN]^{3+}$
**ΛFe1–ΔFe2**	10.794	10.978
**ΛFe1–ΛFe3**	12.159	12.056
**ΛFe1–ΔFe4**	10.788	11.473
**ΔFe2–ΔFe4**	11.291	11.611
**ΔFe2–ΛFe3**	10.783	11.713
**ΛFe3–ΔFe4**	10.794	10.907

5.4.3. Interaction between M^{8+}⊂NCMe

Optimised geometry of **M^{8+}⊂NCMe** (Figure S4.12, interaction energy: –49.8 kJ/mol) showed the solvent guest MeCN within the cavity of **M^{8+}**, with the solvent guest appearing to be receive one long C–H_b···NCMe hydrogen bonds (H_b···NCMe distances: 2.83 Å, 99% of the sum of van der Waals radii[76]).

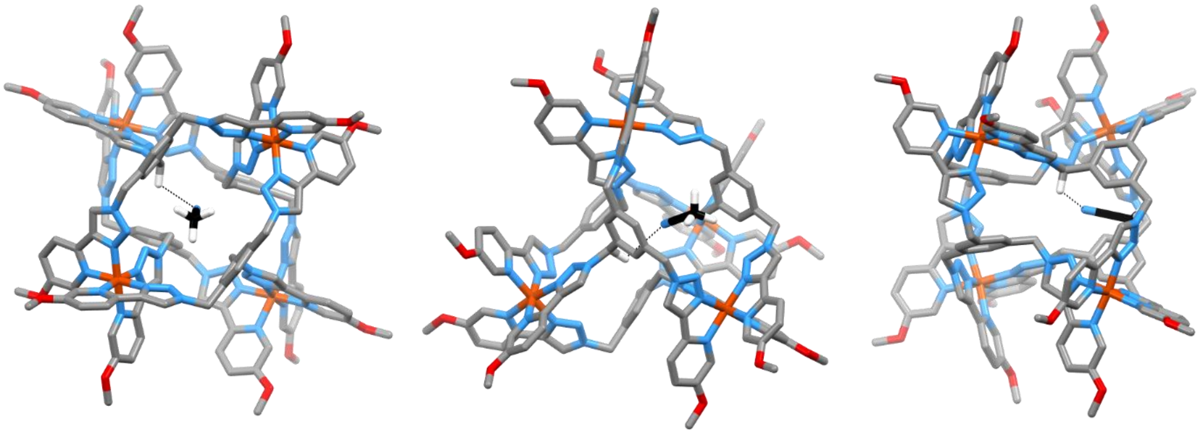

Figure S5.12 Different views of the r^{2}SCAN-3c/CPCM(MeCN)-optimised structure of **M^{8+}⊂NCMe**, dotted lines indicate a close contact shorter that the van der Waals' radii of H and N. Colours: cage carbon: grey, solvent carbon: black, nitrogen: blue, oxygen: red, Λ/Δ –Fe^{II}: orange. Most hydrogen atoms are omitted for clarity.

5.4.4. Interaction between M^{8+}⊂O_2NMe

Optimised geometry of **M^{8+}⊂O_2NMe** (Figure S4.13, interaction energy: –56.3 kJ/mol) showed the solvent guest $MeNO_2$ within the cavity of **M^{8+}**, though it does not appear to interact with the cage interior protons.

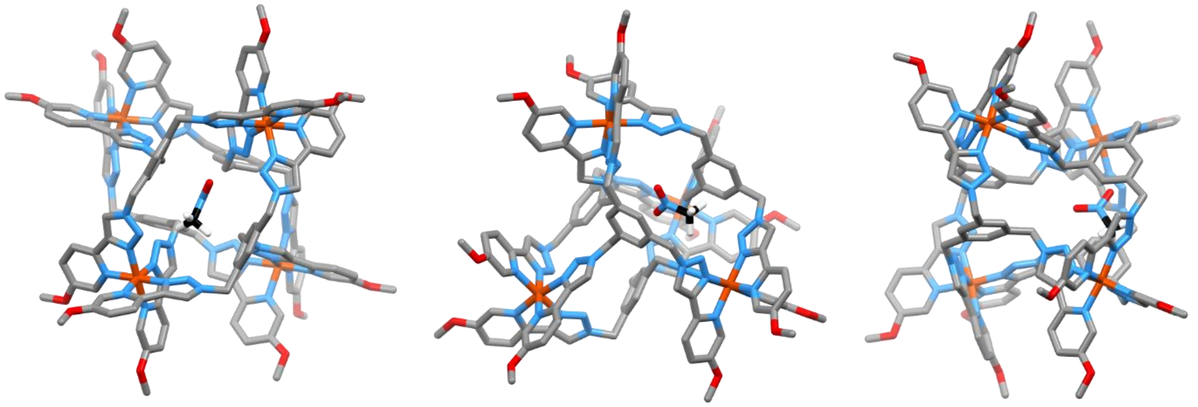

Figure S5.13 Different views of the r^{2}SCAN-3c/CPCM(MeCN)-optimised structure of **M^{8+}⊂O_2NMe**. Colours: cage carbon: grey, solvent carbon: black, nitrogen: blue, oxygen: red, Λ/Δ –Fe^{II}: orange. Most hydrogen atoms are omitted for clarity.

5.4.5. Interaction between $\boldsymbol{D_2}$–H^{8+}⊂NCMe

Optimised geometry of $\boldsymbol{D_2}$**–H^{8+}⊂NCMe** (Figure S4.14, interaction energy: –43.3 kJ/mol) showed the solvent guest MeCN within the cavity of $\boldsymbol{D_2}$**–H^{8+}**, though it does not appear to interact with the cage interior protons.

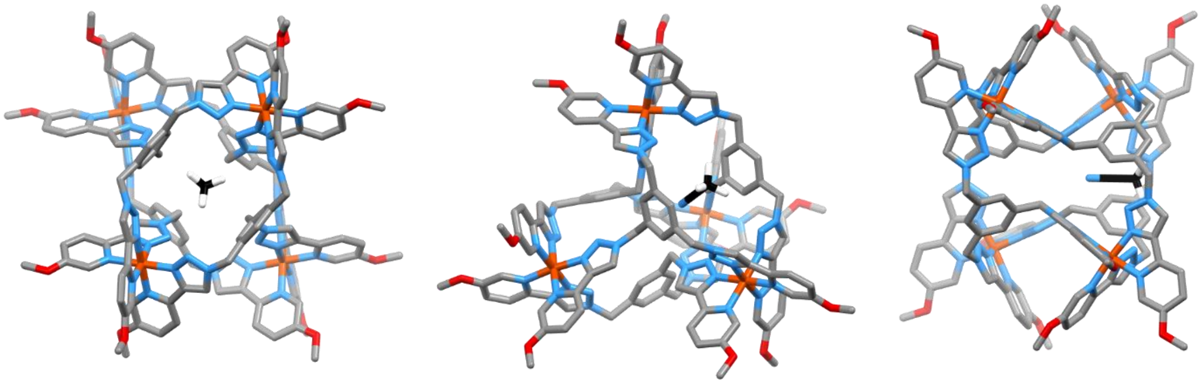

Figure S5.14 Different views of the r^{2}SCAN-3c/CPCM(MeCN)-optimised structure of $\boldsymbol{D_2}$**–H^{8+}⊂NCMe**. Colours: cage carbon: grey, solvent carbon: black, nitrogen: blue, oxygen: red, Λ/Δ –Fe^{II}: orange. Most hydrogen atoms are omitted for clarity.

5.4.6. Interaction between $\boldsymbol{D_2}$–H^{8+}⊂O_2NMe

Optimised geometry of $\boldsymbol{D_2}$**–H^{8+}⊂O_2NMe** (Figure S4.15, interaction energy: –40.4 kJ/mol) showed the solvent guest $MeNO_2$ within the cavity of $\boldsymbol{D_2}$**–H^{8+}**, though it does not appear to interact with the cage interior protons.

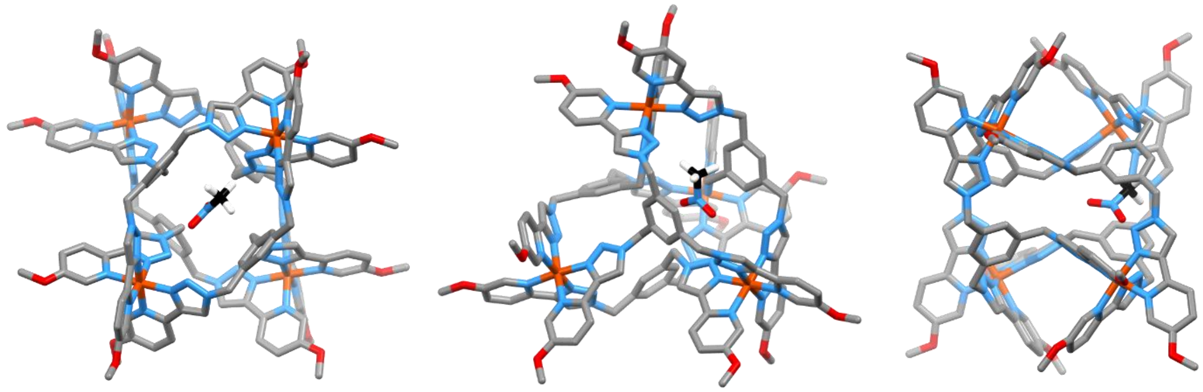

Figure S5.15 Different views of the r^{2}SCAN-3c/CPCM(MeCN)-optimised structure of $\boldsymbol{D_2}$**–H^{8+}⊂O_2NMe**. Colours: cage carbon: grey, solvent carbon: black, nitrogen: blue, oxygen: red, Λ/Δ –Fe^{II}: orange. Most hydrogen atoms are omitted for clarity.

5.5. Cavity calculations

5.5.1. General

To determine the cavity volume and solvent accessible surface areas of $\mathbf{M^{8+}}$ and $\boldsymbol{D_2}$**–**$\mathbf{H^{8+}}$, MoloVol 1.1.1 calculations[103] based on the r^2SCAN-3c/CPCM(MeCN)-optimised structures of $\mathbf{M^{8+}}$, $\boldsymbol{D_2}$**–**$\mathbf{H^{8+}}$, $\mathbf{M^{8+}{\subset}NCMe}$, $\mathbf{M^{8+}{\subset}O_2NMe}$, $\boldsymbol{D_2}$**–**$\mathbf{H^{8+}{\subset}NCMe}$ and $\boldsymbol{D_2}$**–**$\mathbf{H^{8+}{\subset}O_2NMe}$ were performed using both the single-probe and two-probe mode. To determine the cavity volume changes upon solvent encapsulation, the solvent guest from each relevant r^2SCAN-3c/CPCM(MeCN)-optimised structure ($\mathbf{M^{8+}{\subset}NCMe}$, $\mathbf{M^{8+}{\subset}O_2NMe}$, $\boldsymbol{D_2}$**–**$\mathbf{H^{8+}{\subset}NCMe}$ and $\boldsymbol{D_2}$**–**$\mathbf{H^{8+}{\subset}O_2NMe}$) was deleted from the geometry coordinates. There were no further changes to these geometries, resulting in tetrahedral structures with effectively 'ghost' solvent guests (denoted as eg. $\mathbf{M^{8+}{\subset}NCMe^{ghost}}$).

The program parameters were as follows: small probe radius: 1.2 Å; large probe radius: 3.0 Å^3; grid resolution: 0.2 Å; optimisation depth: 4. The single probe method was used to calculate probe accessible surface area, while the two probe method was used to calculate interior cavity volumes. Graphics were generated with UCSF ChimeraX 1.6.1. For the single and two-probe graphics, surface level were set to 3 and 1.5 respectively (showing molecular surface (both probes excluded, similar to the Connolly surface)).

5.5.2. Cavity volume and surface area of tetrahedra

5.5.2.1. Tetrahedra interior cavity volume

Analysis of the solvent accessible interior cavities within the tetrahedra $\mathbf{M^{8+}}$, $\mathbf{D_2}$**–**$\mathbf{H^{8+}}$, $\mathbf{M^{8+}{\subset}NCMe^{ghost}}$, $\mathbf{M^{8+}{\subset}O_2NMe^{ghost}}$, $\boldsymbol{D_2}$**–**$\mathbf{H^{8+}{\subset}NCMe^{ghost}}$ and $\boldsymbol{D_2}$**–**$\mathbf{H^{8+}{\subset}O_2NMe^{ghost}}$ indicates that $\boldsymbol{D_2}$**–**$\mathbf{H^{8+}}$, $\boldsymbol{D_2}$**–**$\mathbf{H^{8+}{\subset}NCMe^{ghost}}$ and $\boldsymbol{D_2}$**–**$\mathbf{H^{8+}{\subset}O_2NMe^{ghost}}$ maintain consistent cavity sizes of ~77 Å^3 (green cavity) and ~41 Å^3 (orange cavity) (Table S5), with minimal change seen in either cavity with inclusion of a solvent guest. In contrast, there are substantial changes in interior cavity size between $\mathbf{M^{8+}}$, $\mathbf{M^{8+}{\subset}NCMe^{ghost}}$ and $\mathbf{M^{8+}{\subset}O_2NMe^{ghost}}$, with the initial cavities within $\mathbf{M^{8+}}$ being mostly equal (~51 Å^3). Upon encapsulation of MeCN and $MeNO_2$, one cavity (green) within $\mathbf{M^{8+}}$ expands to 85 Å^3 and 89 Å^3, respectively, in order to accommodate the solvent guest, while the second cavity (yellow), collapses and is inaccessible to solvent (and the small probe). See Sections S4.5.3 to S4.5.13 for relevant graphics.

5.5.2.1. Tetrahedra surface area

Analysis of the probe excluded surface area (*ie* Connolly surface) indicates that overall, $\mathbf{M^{8+}}$ and its solvent analogues have a lower solvent accessible surface area, compared to $\boldsymbol{D_2}$**–**$\mathbf{H^{8+}}$ and its solvent analogues.

Table S5 Interior cavity occupied volumes (Å^3) of reported tetrahedra.

Tetrahedra	Interior cavity occupied volume (Å^3)			Connolly surface area (Å^2)
	Green cavity	Yellow cavity	Total interior cavity occupied volume (Å^3)	
M^{8+}	48.2	54.6	103	1988.68
$M^{8+}\subset NCMe^{ghost}$	85.3	0	85.3	1937.44
$M^{8+}\subset O_2NMe^{ghost}$	89.1	0	89.1	1940.30
$M^{8+}\subset NCMe$	0	0	0	1877.71
$M^{8+}\subset O_2NMe$	0	0	0	1881.13
D_2–H^{8+}	78.9	41.9	121	1981.10
D_2–$H^{8+}\subset NCMe^{ghost}$	76.4	39.8	116	1966.86
D_2–$H^{8+}\subset O_2NMe^{ghost}$	76.1	41.2	117	1962.87
D_2–$H^{8+}\subset NCMe$	0	39.8	39.8	1905.20
D_2–$H^{8+}\subset O_2NMe$	0	41.2	41.2	1906.48
T–H^{8+}	-	-	196.2	1992.14

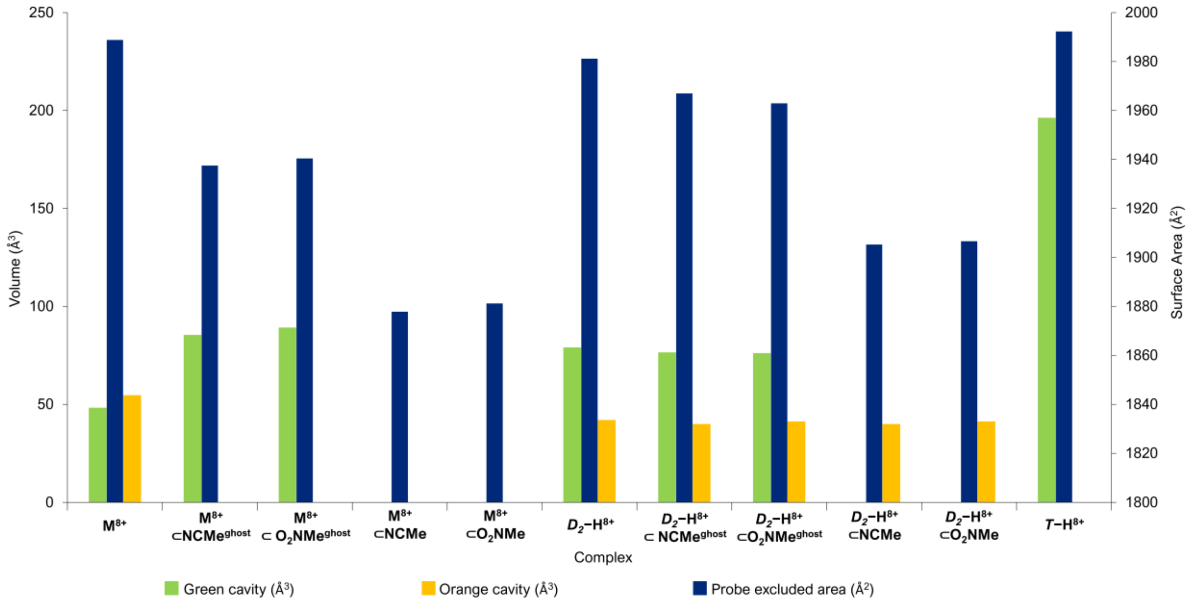

Figure S5.16 Graph comparing the interior cavity occupied volumes (Å^3) and the Connolly surface area (Å^2) of reported tetrahedra.

5.5.3. Interior cavity volume and probe excluded surface area of M^{8+}

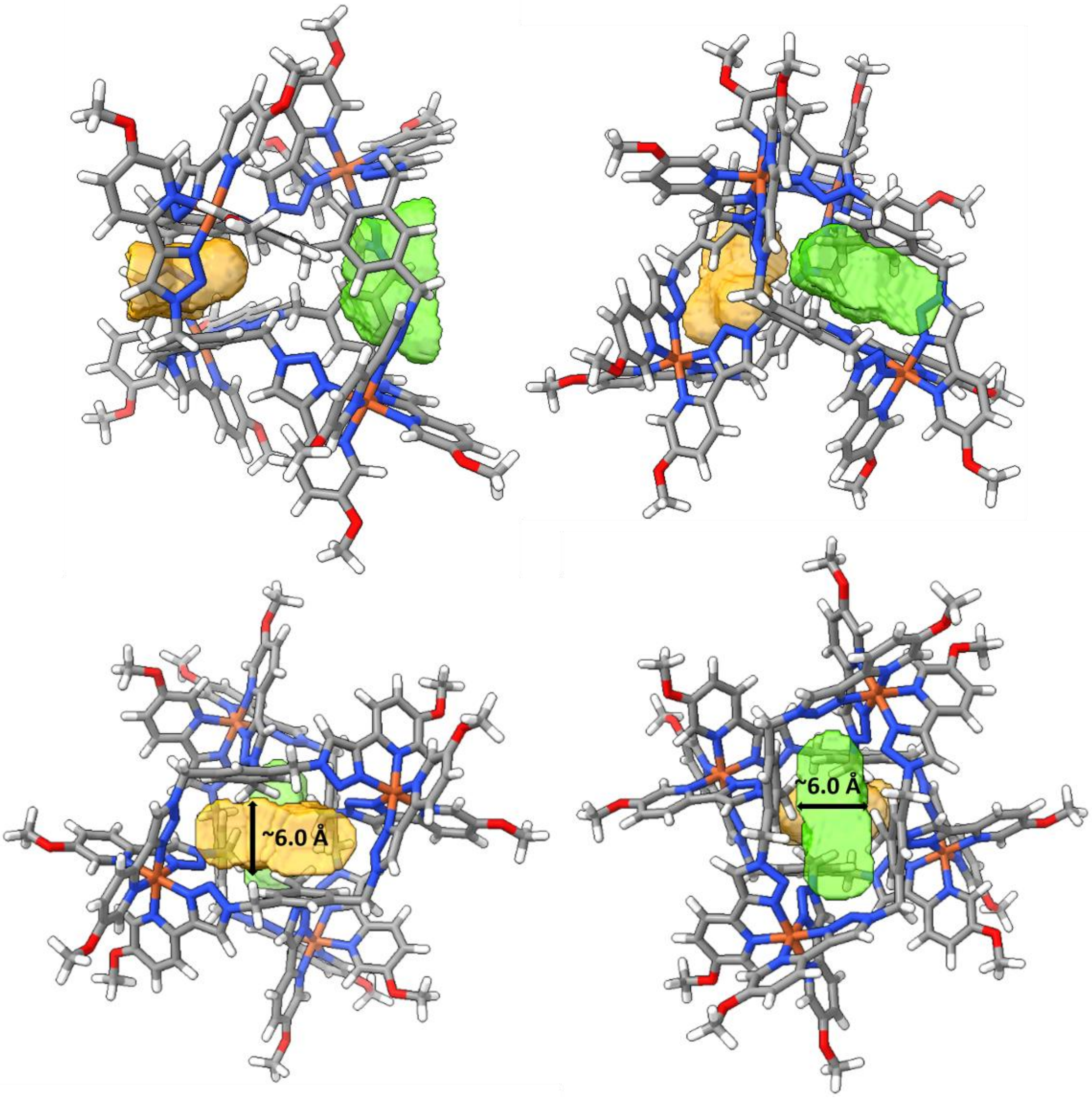

Figure S5.17 Different views of the MoloVol-calculated interior cavity volumes of $\mathbf{M^{8+}}$ with approximate pocket size distance (Å) as shown. The tetrahedron is stick representation, while the internal cavities are shown as a green area (48.2 Å^3) and yellow area (54.6 Å^3).

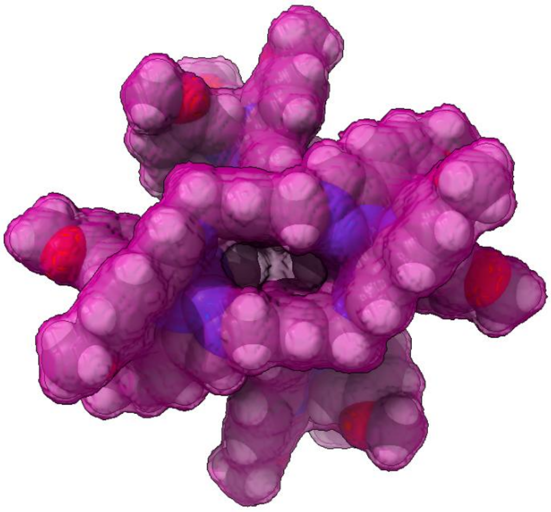

Figure S5.18 MoloVol-calculated probe excluded surface area of $\mathbf{M^{8+}}$. The tetrahedron is space-filling representation, while the probe excluded surface area is a pink surface (1988.68 Å^2).

5.5.4. Interior cavity volume and probe excluded surface area of M^{8+}⊂NCMe*ghost*

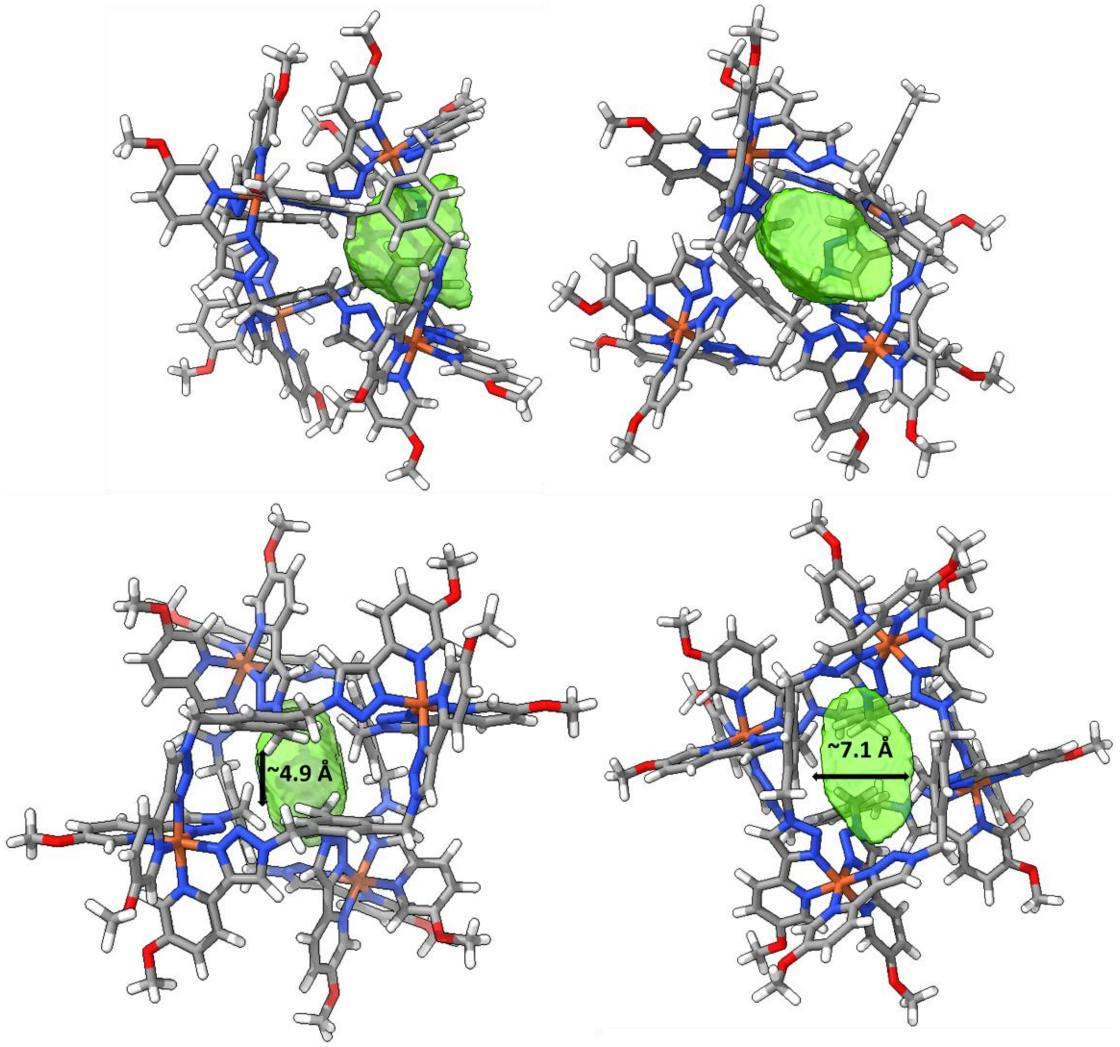

Figure S5.19 Different views of the MoloVol-calculated interior cavity volumes of **M^{8+}⊂NCMe*ghost*** with approximate pocket size distance (Å) as shown. The tetrahedron is stick representation, while the internal cavity is shown as a green area (85.3 Å^3).

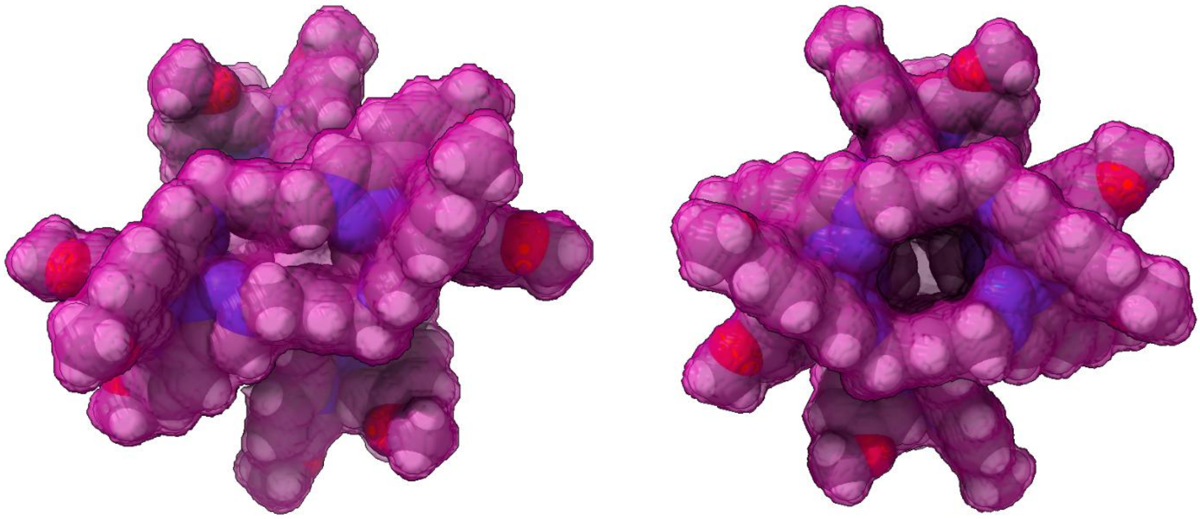

Figure S5.20 MoloVol-calculated probe excluded surface area of **M^{8+}⊂NCMe*ghost***. The tetrahedron is space-filling representation, while the probe excluded surface area is a pink surface (1937.44 Å^2).

5.5.5. Interior cavity volume and probe excluded surface area of $\mathbf{M^{8+}{\subset}O_2NMe}^{ghost}$

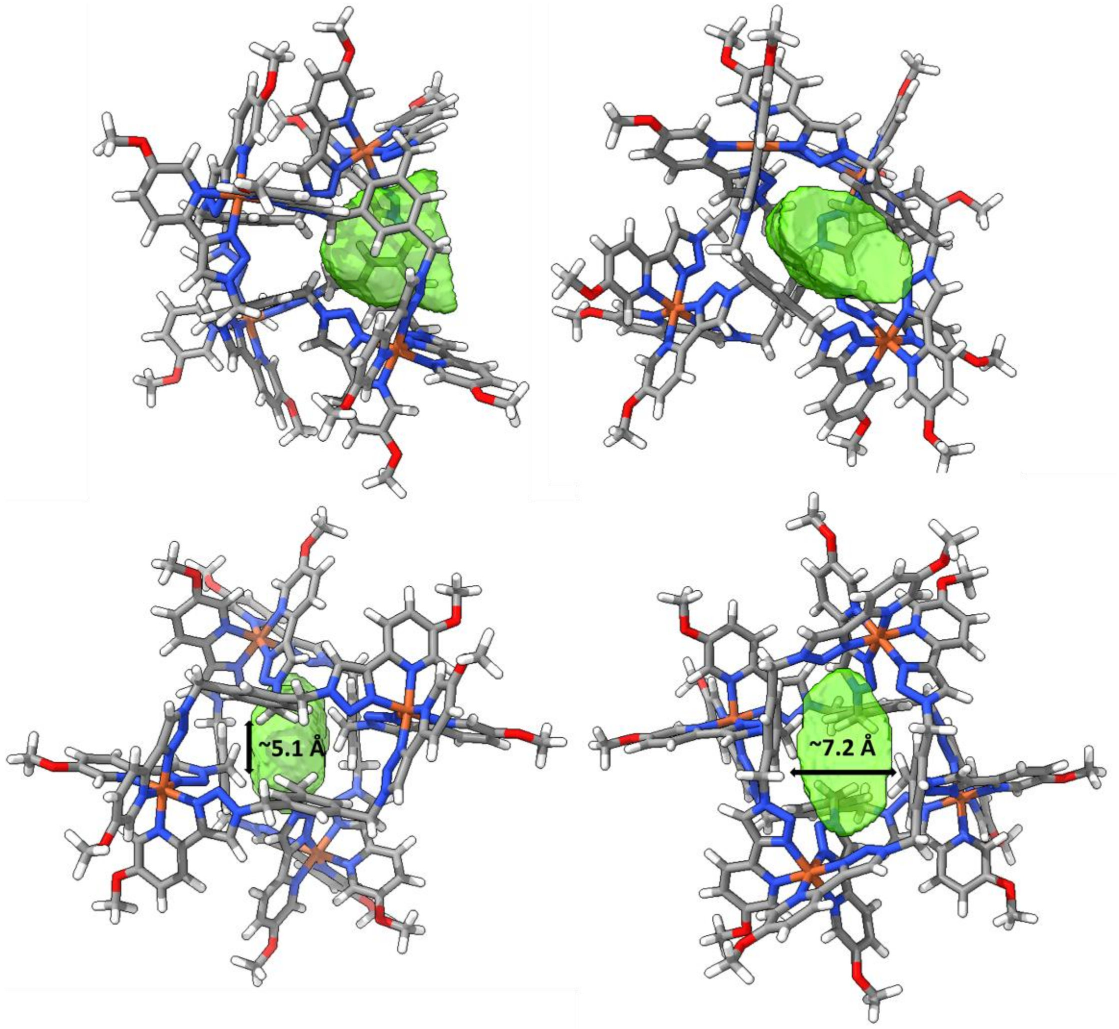

Figure S5.21 Different views of the MoloVol-calculated interior cavity volumes of $\mathbf{M^{8+}{\subset}O_2NMe}^{ghost}$ with approximate pocket size distance (Å) as shown. The tetrahedron is stick representation, while the internal cavity is shown as a green area (89.1 $Å^3$).

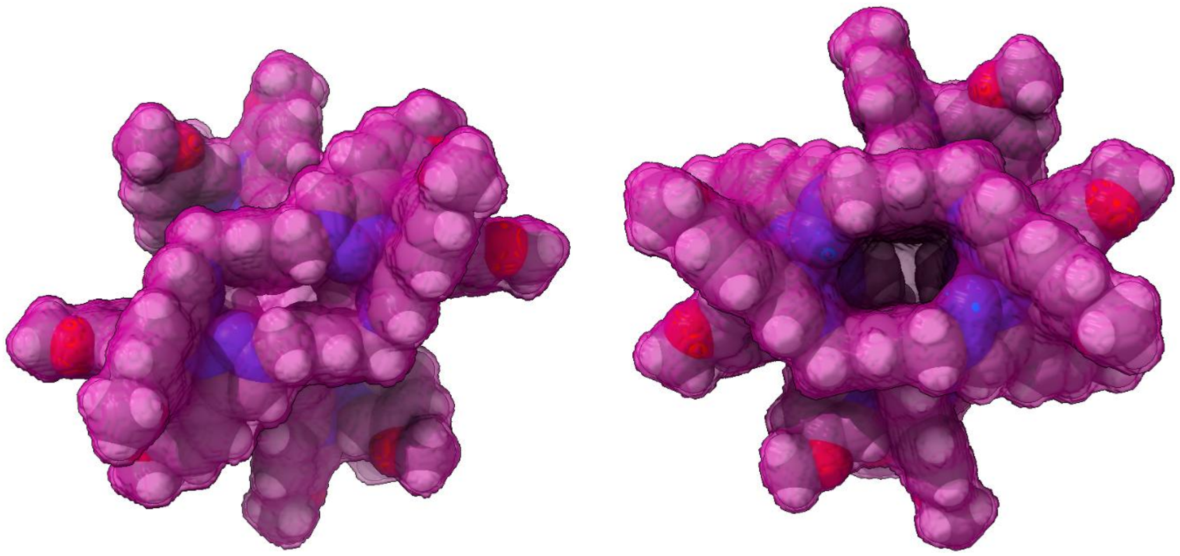

Figure S5.22 MoloVol-calculated probe excluded surface area of $\mathbf{M^{8+}{\subset}O_2NMe}^{ghost}$. The tetrahedron is space-filling representation, while the probe excluded surface area is a pink surface (1940.30 $Å^2$).

5.5.6. Probe excluded surface area of $M^{8+} \subset NCMe$

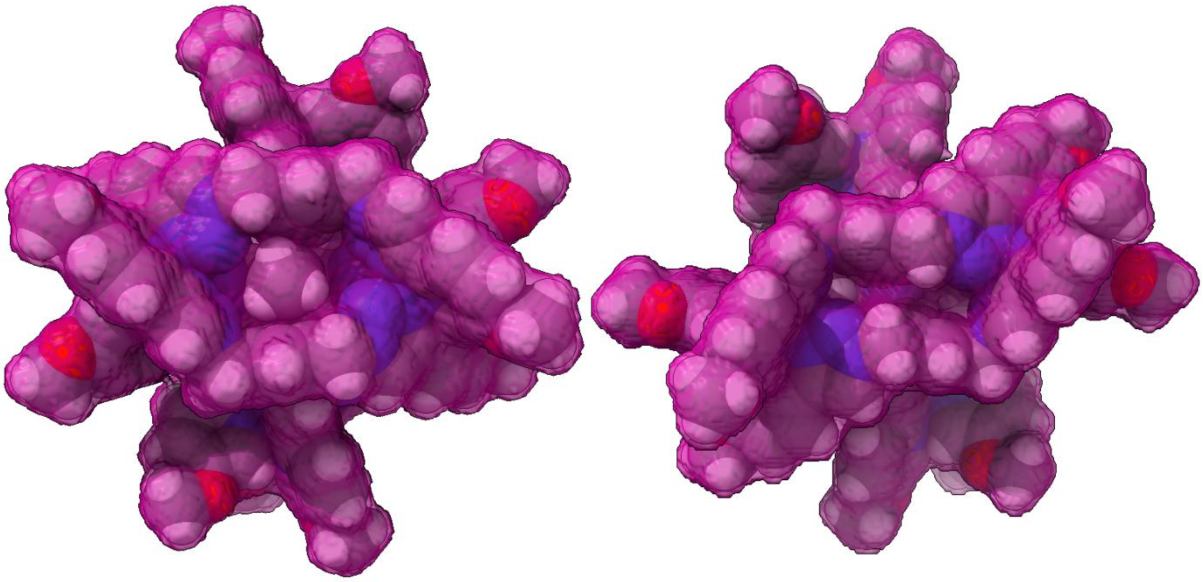

Figure S5.23 MoloVol-calculated probe excluded surface area of **$M^{8+} \subset$NCMe**. The tetrahedron is space-filling representation, while the probe excluded surface area is a pink surface (1877.71 Å^2).

5.5.7. Probe excluded surface area of $M^{8+} \subset O_2NMe$

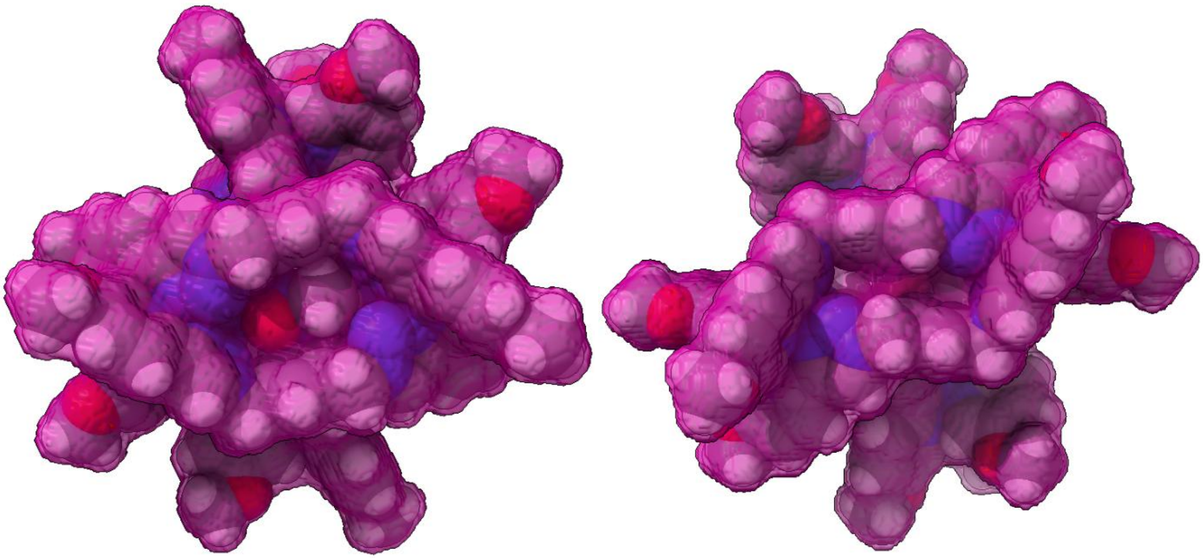

Figure S5.24 MoloVol-calculated probe excluded surface area of **$M^{8+} \subset O_2NMe$**. The tetrahedron is space-filling representation, while the probe excluded surface area is a pink surface (1881.13 Å^2).

5.5.8. Interior cavity volume and probe excluded surface area of D_2–H^{8+}

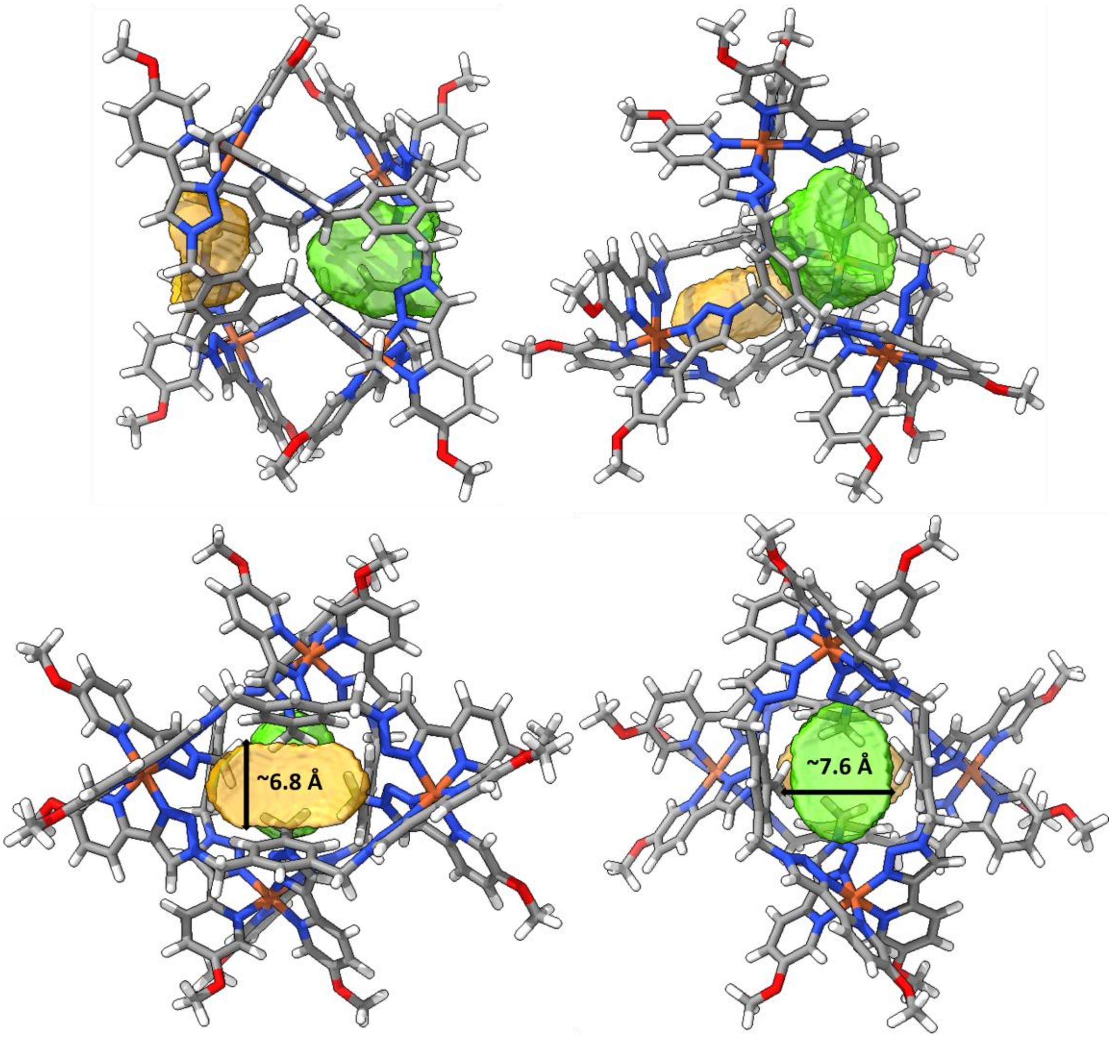

Figure S5.25 Different views of the MoloVol-calculated interior cavity volumes of $\boldsymbol{D_2}$–$\mathbf{H^{8+}}$ with approximate pocket size distance (Å) as shown. The tetrahedron is stick representation, while the internal cavities are shown as a green area (78.9 $Å^3$) and yellow area (41.9 $Å^3$).

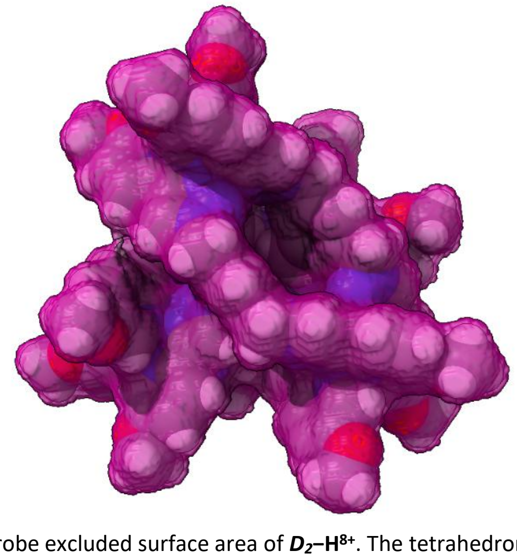

Figure S5.26 MoloVol-calculated probe excluded surface area of $\boldsymbol{D_2}$–$\mathbf{H^{8+}}$. The tetrahedron is space-filling representation, while the probe excluded surface area is a pink surface (1981.10 $Å^2$).

5.5.9. Interior cavity volume and probe excluded surface area of D_2–H^{8+}⊂NCMe^{ghost}

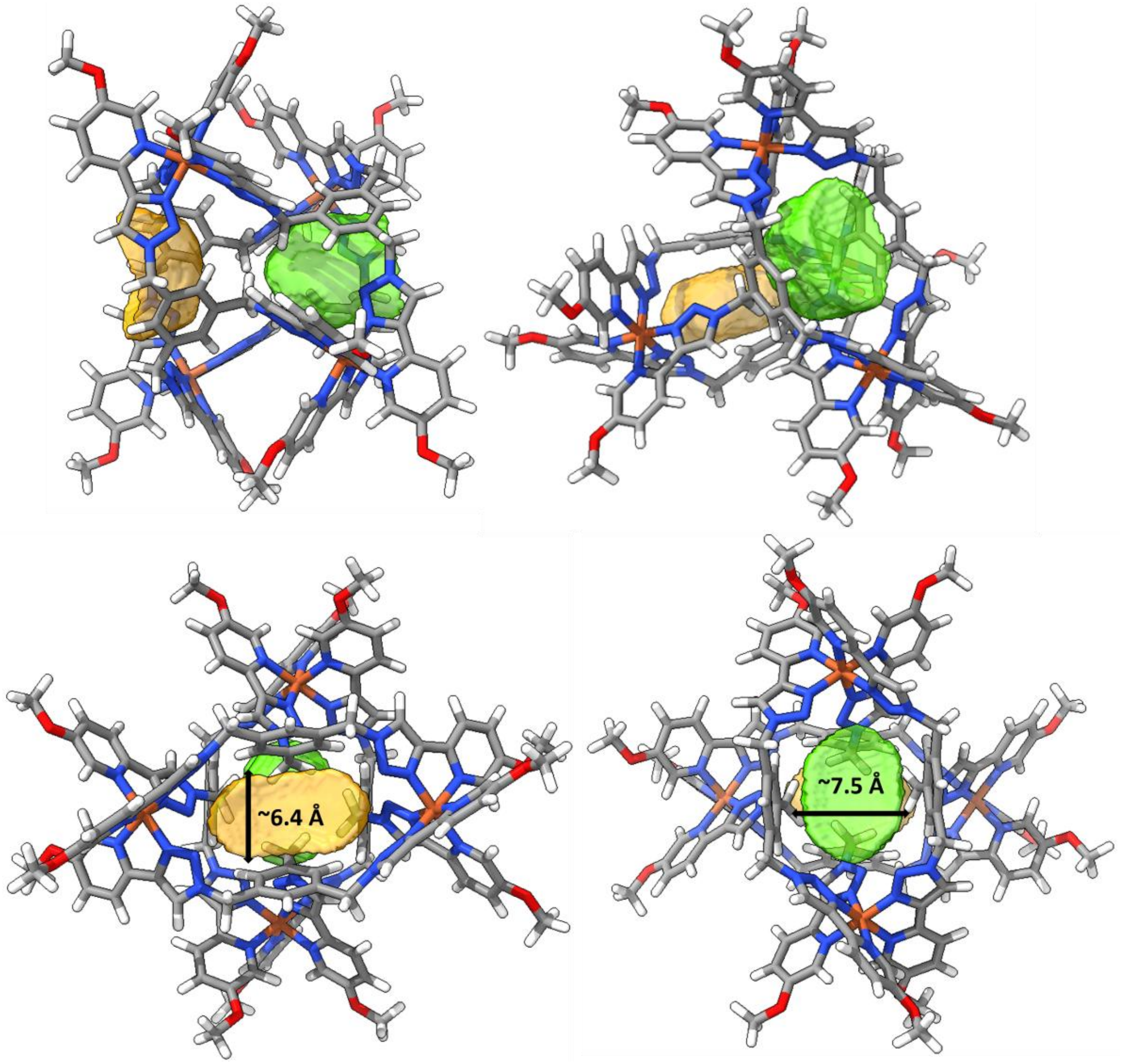

Figure S5.27 Different views of the MoloVol-calculated interior cavity volumes of D_2–H^{8+}⊂NCMe^{ghost} with approximate pocket size distance (Å) as shown. The tetrahedron is stick representation, while the internal cavities are shown as a green area (76.4 Å^3) and yellow area (39.8 Å^3).

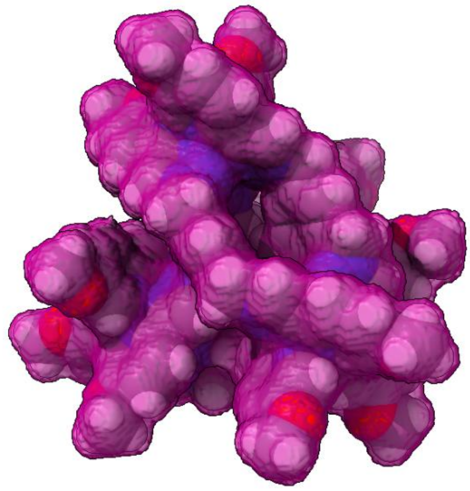

Figure S5.28 MoloVol-calculated probe excluded surface area of D_2–H^{8+}⊂NCMe^{ghost}. The tetrahedron is space-filling representation, while the probe excluded surface area is a pink surface (1966.86 Å^2).

5.5.10. Interior cavity volume and probe excluded surface area of $\boldsymbol{D_2}$–$H^{8+}\subset O_2NMe^{ghost}$

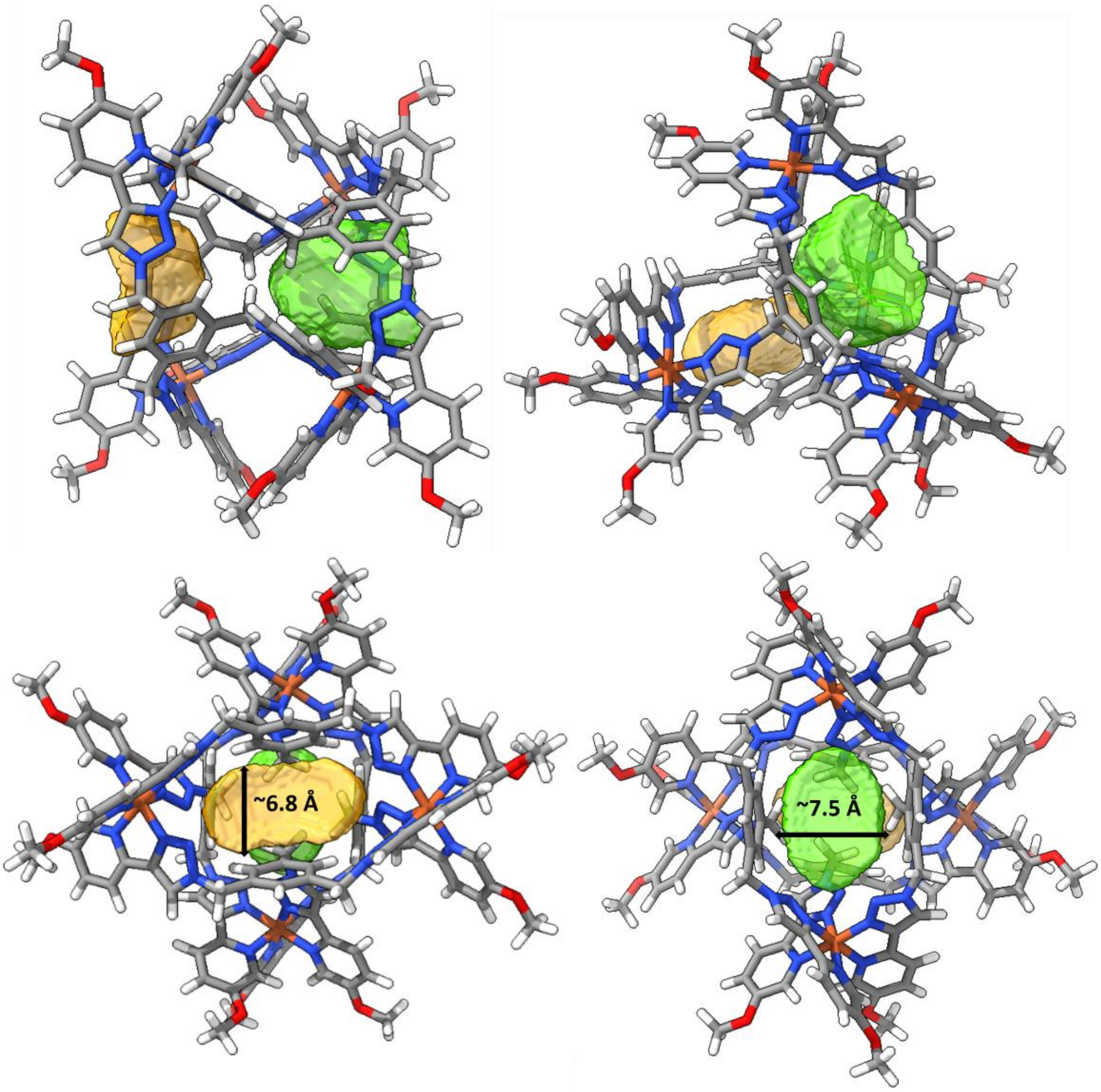

Figure S5.29 Different views of the MoloVol-calculated interior cavity volumes of $\boldsymbol{D_2}$–$H^{8+}\subset O_2NMe^{ghost}$ with approximate pocket size distance (Å) as shown. The tetrahedron is stick representation, while the internal cavities are shown as a green area (76.1 $Å^3$) and yellow area (41.2 $Å^3$).

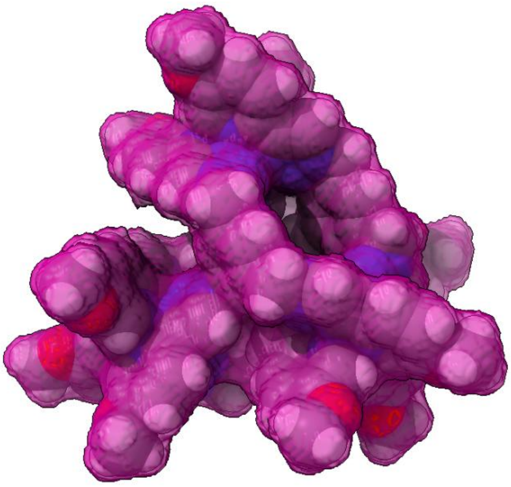

Figure S5.30 MoloVol-calculated probe excluded surface area of $\boldsymbol{D_2}$–$H^{8+}\subset O_2NMe^{ghost}$. The tetrahedron is space-filling representation, while the probe excluded surface area is a pink surface (1962.87 $Å^2$).

5.5.11. Interior cavity volume and probe excluded surface area of D_2–H^{8+}⊂NCMe

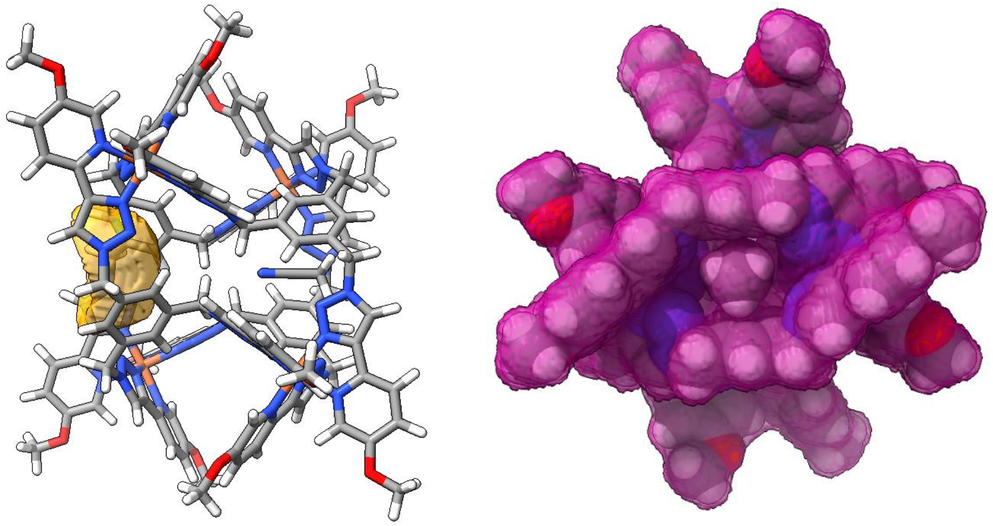

Figure S5.31 Different views of the MoloVol-calculated a) interior cavity volume (stick representation with unoccupied cavity shown as a yellow area (39.8 Å^3)) and b) probe excluded surface area of ***D*₂–H^8+^⊂NCMe** (space-filling representation, while the probe excluded surface area is a pink surface (1905.20 Å^2)).

5.5.12. Interior cavity volume and probe excluded surface area of *D2*–H^{8+}⊂O_2NMe

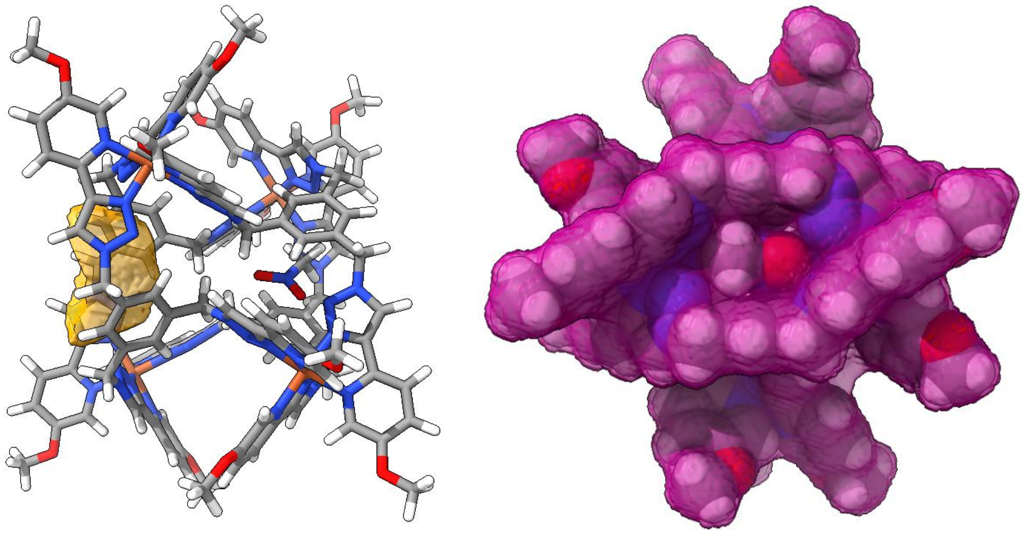

Figure S5.32 Different views of the MoloVol-calculated a) interior cavity volume (stick representation with unoccupied cavity shown as a yellow area (41.2 Å^3)) and b) probe excluded surface area of **D_2–H^{8+}⊂O_2NMe (**space-filling representation, while the probe excluded surface area is a pink surface (1906.48 Å^2)).

5.5.13. Interior cavity volume and probe excluded surface area of *T*–H^{8+}

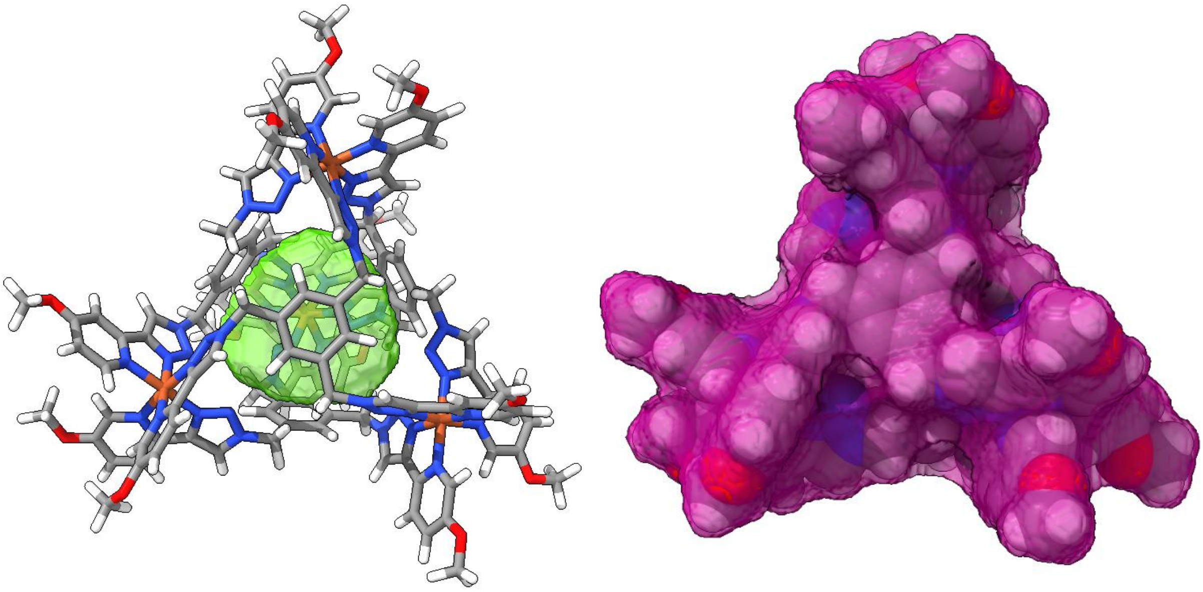

Figure S5.33 Different views of the MoloVol-calculated a) interior cavity volume (stick representation with unoccupied cavity shown as a green area (196.2 Å^3)) and b) probe excluded surface area of ***T*–H^{8+}** (space-filling representation, while the probe excluded surface area is a pink surface (1905.20 Å^2)).

6. References

Note: reference numbering is contiguous with the main manuscript

[30] J. K. Clegg, J. Cremers, A. J. Hogben, B. Breiner, M. M. J. Smulders, J. D. Thoburn, J. R. Nitschke, *Chem. Sci.* **2013**, *4*, 68–76.
[47] R. W. Saalfrank, H. Maid, A. Scheurer, R. Puchta, W. Bauer, *Eur. J. Inorg. Chem.* **2010**, *2010*, 2903–2906.
[76] S. Alvarez, *Dalton Trans.* **2013**, *42*, 8617–8636.
[77] O. V. Dolomanov, L. J. Bourhis, R. J. Gildea, J. A. K. Howard, H. Puschmann, *J. Appl. Crystallogr.* **2009**, *42*, 339–341.
[78] C. Plett, S. Grimme, *Angew. Chem. Int. Ed.* **2023**, *62*, e202214477.
[79] S. Grimme, C. Bannwarth, E. Caldeweyher, J. Pisarek, A. Hansen, *J. Chem. Phys.* **2017**, *147*, 161708.
[80] S. Grimme, C. Bannwarth, P. Shushkov, *J. Chem. Theory Comput.* **2017**, *13*, 1989–2009.
[81] S. Grimme, A. Hansen, S. Ehlert, J.-M. Mewes, *J. Chem. Phys.* **2021**, *154*, 064103.
[82] F. Weigend, R. Ahlrichs, *Phys. Chem. Chem. Phys.* **2005**, *7*, 3297–3305.
[103] J. B. Maglic, R. Lavendomme *J Appl Cryst* **2022**, *55*, 1033–1044.
[105] R. Hooper, L. J. Lyons, M. K. Mapes, D. Schumacher, D. A. Moline, R. West, *Macromolecules* **2001**, *34*, 931–936.
[106] H. E. Gottlieb, V. Kotlyar, A. Nudelman, *J. Org. Chem.* **1997**, *62*, 7512–7515.
[107] K. Stott, J. Stonehouse, J. Keeler, T.-L. Hwang, A. J. Shaka, *J. Am. Chem. Soc.* **1995**, *117*, 4199–4200.
[108] J. Jeener, B. H. Meier, P. Bachmann, R. R. Ernst, *J. Chem. Phys.* **1979**, *71*, 4546–4553.
[109] R. Wagner, S. Berger, *J. Magn. Reson. A* **1996**, *123*, 119–121.
[110] A. Jerschow, N. Müller, *J. Magn. Reson. A* **1996**, *123*, 222–225.
[111] A. Jerschow, N. Müller, *J. Magn. Reson.* **1997**, *125*, 372–375.
[112] http://app.supramolecular.org/bindfit/.
[113] P. Thordarson, *Chem. Soc. Rev.* **2011**, *40*, 1305–1323.
[114] CrysAlis PRO, **2011**.
[115] G. Sheldrick, *Acta Crystallogr. Sect. A* **2015**, *71*, 3–8.
[116] G. Sheldrick, *Acta Crystallogr. Sect. C* **2015**, *71*, 3–8.
[117] D. Kratzert, J. J. Holstein, I. Krossing *J Appl Cryst* **2015**, *48*, 933–938.
[118] D. Kratzert, I. Krossing, *J Appl Cryst* **2018**, *51*, 928–934.
[119] R. A. S. Vasdev, D. Preston, S. Ø. Scottwell, H. J. L. Brooks, J. D. Crowley, M. P. Schramm, *Molecules* **2016**, *21*, DOI 10.3390/molecules21111548.
[120] Spartan'24 v. 1.0.0., **2024**.
[121] G. Sigalov, A. Fenley, A. Onufriev, *J. Chem. Phys.* **2006**, *124*, 124902.
[122] V. I. Lebedev, *Sib. Math. J.* **1977**, *18*, 99–107.
[123] J.-P. Ryckaert, G. Ciccotti, H. J. C. Berendsen, *J. Comput. Phys.* **1977**, *23*, 327–341.
[124] C. Bannwarth, S. Ehlert, S. Grimme, *J. Chem. Theory Comput.* **2019**, *15*, 1652–1671.
[125] F. Neese, *WIREs Comput. Mol. Sci.* **2022**, *12*, e1606.
[126] M. Bursch, J.-M. Mewes, A. Hansen, S. Grimme, *Angew. Chem. Int. Ed.* **2022**, *61*, e202205735.
